# Dielectric-dependent hybrid functional based on meta-GGA

**DOI:** 10.1038/s41467-026-75146-x

**Published:** 2026-07-04

**Authors:** Stefan Riemelmoser, Xun Xu, Alfredo Pasquarello

**Affiliations:** 1 Chaire de Simulation à l’Echelle Atomique (CSEA), Ecole Polytechnique Fédérale de Lausanne (EPFL), Lausanne, Switzerland; 2 Beijing Computational Science Research Center, Beijing, China

**Keywords:** Electronic structure, Electronic properties and materials, Density functional theory

## Abstract

Semilocal density functionals such as the Perdew-Burke-Ernzerhof (PBE) functional substantially underestimate experimental band gaps. Hybrid functionals address this band gap problem by admixing a fraction of Fock exchange to semilocal exchange. The optimal mixing parameter depends on the specific material and can be identified as the inverse dielectric constant (dielectric-dependent hybrid functional, DDH). Here, we show that dielectric constants obtained using the r^2^SCAN meta-GGA functional are significantly more accurate than dielectric constants obtained using the semilocal PBE functional. We propose the DD-r^2^SCANH functional, a dielectric-dependent hybrid functional based on r^2^SCAN. DD-r^2^SCANH can outperform the standard PBE-based DDH in terms of band gaps and other electronic structure properties. Particularly marked improvements are obtained for narrow-gap semiconductors such as Ge and InAs, where PBE wrongly predicts a metallic phase, but r^2^SCAN opens a band gap.

## Abstract (web summary)

A density functional is proposed that combines a modern treatment of semilocal interactions with an exact constraint for long-range dielectric screening. The method achieves gold-standard accuracy for energy levels while being fast and user-friendly.

## Introduction

Kohn–Sham density functional theory^1,2^ (DFT) has become the workhorse for first principles calculations in materials science and quantum chemistry. The success of DFT is founded on the efficacy of its semilocal approximations. After its inception in the 1960s, DFT was synonymous with the canonical local density approximation (LDA) for several decades^3^. Since the 1990s, the generalized gradient approximations (GGA) have revolutionized DFT and led to its widespread use^4,5^. In particular, the PBE functional (Perdew-Burke-Ernzerhof)^6^ has become a standard for solid-state applications. PBE is reasonably accurate and well-balanced for many materials and properties. The success of PBE stems from its elegant functional form based on exact constraints. By construction, PBE approaches the exact (unknown) exchange-correlation functional in important physical limits and fulfills 11 constraints in total. More recently, the r^2^SCAN meta-GGA functional has emerged as a successor to PBE^7^. r^2^SCAN is an improved version of the SCAN functional (strongly constrained and appropriately normed)^7–12^. r^2^SCAN avoids the numerical instabilities that plagued SCAN^7,10–12^, but keeps all 17 known exact constraints that a meta-GGA functional can fulfill. Thus, r^2^SCAN brings remarkable and consistent improvements over PBE at a modest overhead in computational cost, making r^2^SCAN a prime choice for high-throughput studies^13–15^.

The Hohenberg–Kohn theorems guarantee that Kohn–Sham DFT correctly predicts important ground state properties, including the electron density and the total energy, as well as other ground state properties that can be extracted from them (provided the exact exchange-correlation functional is used). Unfortunately, this guarantee does not apply to all electronic structure properties, and the band gap problem of Kohn–Sham DFT is the most notorious example^16^. In GW theory^17–19^, the fundamental gap can be calculated as the difference of quasiparticle excitation energies, *E*_g_ = CBM - VBM. However, when the band gap is calculated in this way using the Kohn–Sham eigenvalues, PBE and other semilocal functionals like LDA drastically underestimate experimental band gaps. This failure has two components: (i) the use of the approximate Kohn–Sham DFT functional instead of the exact one and (ii) the missing derivative discontinuity, associated with the use of a local exchange-correlation potential in Kohn–Sham DFT^20,21^. To address point (i), more accurate local exchange-correlation potentials can be constructed either via Kohn–Sham inversion^22,23^ or through the optimized effective potential method^10,24^. However, the band gaps obtained with these methods barely improve upon PBE. It is therefore crucial to address point (ii), the derivative discontinuity.

In generalized Kohn–Sham schemes (GKS), one incorporates the derivative discontinuity by adopting nonlocal exchange-correlation potentials^2,25,26^. Meta-GGA functionals achieve this nonlocality via dependence on the kinetic energy density^27–30^. The band gaps obtained by SCAN and r^2^SCAN systematically improve upon PBE band gaps, but remain underestimated with respect to experiment^10,14,31,32^. Hartree–Fock theory is also a GKS scheme, but drastically overestimates band gaps due to the lack of nonlocal correlation effects. Overall, this means that just the GKS scheme alone is not sufficient, and it is crucial to address both points (i) and (ii) simultaneously to achieve realistic band gaps^25,33,34^. Hybrid functionals include nonlocality by replacing a fraction of PBE exchange with Fock exchange. A global hybrid functional (PBEH) uses a single mixing parameter *α*,^35,36^

1 $${E}_{{{{\rm{xc}}}}}^{{{{\rm{PBEH}}}}}(\alpha )=\alpha {E}_{{{{\rm{x}}}}}+(1-\alpha ){E}_{{{{\rm{x}}}}}^{{{{\rm{PBE}}}}}+{E}_{{{{\rm{c}}}}}^{{{{\rm{PBE}}}}}.$$

Since Hartree–Fock (+PBE correlation) generally overestimates experimental band gaps, and PBE underestimates them, one can find a parameter *α* that reproduces the experimental band gap. Perdew, Ernzerhof, and Burke^36^ have suggested a universal value of *α* = 1/4 using arguments from adiabatic connection and perturbation theory (PBE0). However, the authors also realized that “an ideal hybrid would be sophisticated enough to optimize [*α*] for each system and property”^36^. Alkauskas et al.^37^ have shown that the optimal *α* can be identified as the inverse dielectric constant 1/*ε*_*∞*_, see also refs. ^38–43^. Further advances were made by Galli and coworkers^44–46^. They have addressed the issue of how to self-consistently calculate ${\varepsilon }_{\infty }^{-1}$ from first principles, and coined this approach dielectric-dependent hybrid functional (DDH)^44^. Furthermore, they generalized the DDH formalism to finite systems and strongly heterogeneous systems, where $\alpha={\varepsilon }_{\infty }^{-1}$ cannot be straightforwardly applied^46,47^. Importantly, they found that the band gaps predicted by DDH improve drastically upon PBE0^44^. However, DDH systematically underestimates semiconductor gaps^45^, and for narrow-gap semiconductors such as InAs, DDH can even wrongly predict a metallic phase^48,49^. Previous studies have attempted to improve upon the standard DDH via range-separation approaches^45,48,50–54^, we refer to Zhang et al.^55^ for a detailed review. While range-separation has overall improved the performance of the DDH, all these schemes involve some arbitrariness in the way that the Coulomb interaction is partitioned into long-range and short-range. Furthermore, some of the range-separated hybrid functionals also include semi-empirical parameters^45,48^.

An alternative way of improving hybrid functionals is to optimize the DFT base functional in Eq. (1). This idea dates back to the original work of Becke^35^, and hybrid functionals based on early versions of meta-GGA have been broadly used in quantum chemistry. More recently, hybrid functionals based on SCAN^56^ and r^2^SCAN^57,58^ have also been suggested. However, these functionals all employ a fixed *α* parameter, limiting accuracy.

Here, we propose to combine Becke’s idea with the DDH formalism. Namely, we replace the PBE part in Eq. (1) by r^2^SCAN, and use the optimal mixing parameter $\alpha={\varepsilon }_{\infty }^{-1}$. We show that PBE systematically overestimates the dielectric constants of semiconductors, and especially narrow-gap semiconductors. This overscreening is mirrored by the dielectric-dependent hybrid functional based on PBE (DD-PBEH) that underestimates semiconductor band gaps. We show that the dielectric constants obtained by r^2^SCAN improve significantly upon PBE dielectric constants. Finally, we demonstrate that the improvements in electronic structure are inherited by our dielectric-dependent hybrid functional based on r^2^SCAN (DD-r^2^SCANH). Comparing several DFT base functionals, r^2^SCAN is shown to be optimally compatible with DDH. DD-r^2^SCANH consistently outperforms DD-PBEH for various electronic structure properties. Notably, DD-r^2^SCANH can reach the accuracy of gold-standard GW methods^59^ for both band gaps and ionization potentials.

## Results

### Theoretical background: GKS theory

In the following, we briefly review the fundamentals of generalized Kohn–Sham theory for hybrid functionals^25,33,60^. We start by writing down the hybrid functional GKS equation^60^

2 $$\left(-\frac{1}{2}{\nabla }^{2}+{v}_{{{{\rm{ext}}}}}({{{\bf{r}}}})+{v}_{{{{\rm{H}}}}}({{{\bf{r}}}})+\alpha {\Sigma }_{{{{\rm{x}}}}}+{v}_{{{{\rm{R}}}},{{{\rm{xc}}}}}^{\alpha }({{{\bf{r}}}})\right){\phi }_{i}({{{\bf{r}}}})={\varepsilon }_{i}{\phi }_{i}({{{\bf{r}}}})$$

Here, the *ϕ*_*i*_ are single-particle electronic orbitals with corresponding eigenvalues *ε*_*i*_. The first term inside the brackets is the kinetic energy, *v*_ext_ is the external potential, *v*_H_ is the Hartree potential, and *Σ*_x_ is the nonlocal Fock operator

3 $${\Sigma }_{{{{\rm{x}}}}}({{{\bf{r}}}},{{{{\bf{r}}}}}^{{\prime} })=- {\sum }_{i}^{{{{\rm{occ}}}}}{\phi }_{i}({{{\bf{r}}}}){\phi }_{i}^{*}({{{{\bf{r}}}}}^{{\prime} })\frac{1}{| {{{\bf{r}}}}-{{{{\bf{r}}}}}^{{\prime} }| }.$$

The remainder potential ${v}_{{{{\rm{R,xc}}}}}^{\alpha }$ is an everything-else term that ensures that the density and total energy are exact. Importantly, the remainder potential depends on the choice of the mixing parameter *α*. For *α* = 0, Eq. (2) reduces to the standard KS equation, and ${v}_{{{{\rm{R,xc}}}}}^{\alpha=0}$ is equivalent to the usual KS exchange-correlation potential. For *α* = 1, one obtains the Hartree–Fock–Kohn–Sham equation^25^. A priori, all values of *α* give rise to valid GKS schemes. However, optimal *α* parameters exist in practical applications where the remainder potential has to be approximated. Recently, ref. ^60^ obtained the exact remainder potential for a series of atoms and ions using a GKS inversion procedure. They demonstrated that the spatial extension of ${v}_{{{{\rm{R}}}},{{{\rm{xc}}}}}^{\alpha }$ varies with *α* and is minimal for *α* = 1. This can be understood by the fact that ${v}_{{{{\rm{R}}}},{{{\rm{xc}}}}}^{\alpha }$ falls off as (1 − *α*)/*r* in the asymptotic long-range limit.

In practical applications, the remainder potential is approximated using DFT^60^,

4 $${v}_{{{{\rm{R}}}},{{{\rm{xc}}}}}^{\alpha }\approx (1-\alpha ){v}_{{{{\rm{x}}}}}^{{{{\rm{DFT}}}}}+{v}_{{{{\rm{c}}}}}^{{{{\rm{DFT}}}}}.$$

Note again that the approximate remainder potential generally does not equal the DFT exchange-correlation potential, since the exchange part is multiplied by a factor (1-*α*). When PBE is used as the DFT functional in Eq. (4), Eq. (2) corresponds to the hybrid functional in Eq. (1).

### Connection between DDH and GW

Next, we give a brief theoretical motivation for our DD-r^2^SCANH functional via comparison to GW theory. First, we generalize Eq. (1) by replacing PBE with a nonspecific DFT base functional,

5 $${E}_{{{{\rm{xc}}}}}^{{{{\rm{DFTH}}}}}(\alpha )=\alpha {E}_{{{{\rm{x}}}}}+(1-\alpha ){E}_{{{{\rm{x}}}}}^{{{{\rm{DFT}}}}}+{E}_{{{{\rm{c}}}}}^{{{{\rm{DFT}}}}}.$$

The DFT base functional can be any exchange-correlation functional from the first three rungs of Jacob’s Ladder (LDA, GGA, or meta-GGA). The corresponding GKS equation is obtained by combining Eqs. (2) and (4),

6 $$\left(-\frac{1}{2}{\nabla }^{2}+{v}_{{{{\rm{ext}}}}}({{{\bf{r}}}})+{v}_{{{{\rm{H}}}}}({{{\bf{r}}}})+\alpha {\Sigma }_{{{{\rm{x}}}}}+(1-\alpha ){v}_{{{{\rm{x}}}}}^{{{{\rm{DFT}}}}}({{{\bf{r}}}})+{v}_{{{{\rm{c}}}}}^{{{{\rm{DFT}}}}}({{{\bf{r}}}})\right){\phi }_{i}({{{\bf{r}}}})={\varepsilon }_{i}{\phi }_{i}({{{\bf{r}}}}).$$

Instead of the GKS equation, GW theory uses the quasiparticle equation^17–19^

7 $$\left(-\frac{1}{2}{\nabla }^{2}+{v}_{{{{\rm{ext}}}}}({{{\bf{r}}}})+{v}_{{{{\rm{H}}}}}({{{\bf{r}}}})+{\Sigma }^{\rm GW}(\omega={E}_{i})\right){\Phi }_{i}({{{\bf{r}}}})={E}_{i}{{{\Phi }}}_{i}({{{\bf{r}}}}),$$

where the Φ_*i*_ are the quasiparticle wave functions, and the *E*_*i*_ are the corresponding quasiparticle energies. The GW self-energy *Σ*^GW^ is not only nonlocal like *Σ*_x_, but also frequency-dependent and complex. The real part of *Σ*^GW^ can be rigorously partitioned into a screened exchange part *Σ*^SEX^ and a remainder *Σ*^COH^ (Coulomb hole),

8 $$\,{\mbox{Re}}\,{\Sigma }^{\rm GW}(\omega )={\Sigma }^{{{{\rm{SEX}}}}}(\omega )+{\Sigma }^{{{{\rm{COH}}}}}(\omega ).$$

We refer to the original references for the full expressions and further details^17–19^. Next, we drop the frequency dependence in the screened Coulomb interaction *W*, by setting its value at *ω* = 0. This yields the static COHSEX approximation ^17–19^

9 $${\Sigma }^{{{{\rm{SEX}}}}}({{{\bf{r}}}},{{{{\bf{r}}}}}^{{\prime} }) 	 \approx -{\sum }_{i}^{{{{\rm{occ}}}}}{\phi }_{i}({{{\bf{r}}}}){\phi }_{i}^{*}({{{{\bf{r}}}}}^{{\prime} })W({{{\bf{r}}}},{{{{\bf{r}}}}}^{{\prime} },\omega=0)\\ {\Sigma }^{{{{\rm{COH}}}}}({{{\bf{r}}}},{{{{\bf{r}}}}}^{{\prime} }) 	 \approx -\frac{1}{2}\delta ({{{\bf{r}}}}-{{{{\bf{r}}}}}^{{\prime} })\left[\frac{1}{| {{{\bf{r}}}}-{{{{\bf{r}}}}}^{{\prime} }| }-W({{{\bf{r}}}},{{{{\bf{r}}}}}^{{\prime} },\omega=0)\right],$$

which also implies a clear physical interpretation for these two self-energy parts. Namely, the quasiparticle experiences a screened exchange interaction *W* given by

10 $$W({{{\bf{r}}}},{{{{\bf{r}}}}}^{{\prime} },\omega=0)=\int\,{\mbox{d}}\,{{{{\bf{r}}}}}^{{\prime\prime} }{\varepsilon }^{-1}({{{\bf{r}}}},{{{{\bf{r}}}}}^{{\prime\prime} })\frac{1}{| {{{{\bf{r}}}}}^{{\prime\prime} }-{{{{\bf{r}}}}}^{{\prime} }| },$$

and the Coulomb hole describes the interaction potential induced by the rearrangement of the electrons around the quasiparticle. Importantly, the quasiparticle equation (7) reduces to a GKS-type equation within static COHSEX, as *Σ*^GW^ is now real and frequency-independent like *Σ*_x_. Following refs. ^44,48^, we further neglect the dispersion of the static inverse dielectric function *ε*^−1^, yielding

11 $$W({{{\bf{r}}}},{{{{\bf{r}}}}}^{{\prime} },\omega=0)\approx \frac{1}{{\varepsilon }_{\infty }| {{{\bf{r}}}}-{{{{\bf{r}}}}}^{{\prime} }| }.$$

Then, the SEX part reduces to the standard Fock exchange multiplied by ${\varepsilon }_{\infty }^{-1}$, see Eq. (3). By comparing Eqs. (7) and (2), we can identify *Σ*^COH^ with ${v}_{{{{\rm{R,xc}}}}}^{\alpha=1/{\varepsilon }_{\infty }}$, conferring a clear physical interpretation to the remainder potential. Following the standard GKS approach, we use DFT for the remainder potential, yielding

12 $${\Sigma }^{{{{\rm{COH}}}}}\approx (1-{\varepsilon }_{\infty }^{-1}){v}_{{{{\rm{x}}}}}^{{{{\rm{DFT}}}}}+{v}_{{{{\rm{c}}}}}^{{{{\rm{DFT}}}}}.$$

Once we plug the approximated SEX and COH expressions into the quasiparticle equation (7), we obtain the GKS equation for a global hybrid functional with $\alpha={\varepsilon }_{\infty }^{-1}$ [see Eq.(6)]. Setting this mixing parameter also in the corresponding energy functional (5), we obtain the dielectric-dependent hybrid functional

13 $${E}_{{{{\rm{xc}}}}}^{{{{\rm{DD-DFTH}}}}} 	={E}_{{{{\rm{xc}}}}}^{{{{\rm{DFTH}}}}}(\alpha={\varepsilon }_{\infty }^{-1})\\ 	={\varepsilon }_{\infty }^{-1}{E}_{{{{\rm{x}}}}}+(1-{\varepsilon }_{\infty }^{-1}){E}_{{{{\rm{x}}}}}^{{{{\rm{DFT}}}}}+{E}_{{{{\rm{c}}}}}^{{{{\rm{DFT}}}}}.$$

**Fig. 1:**
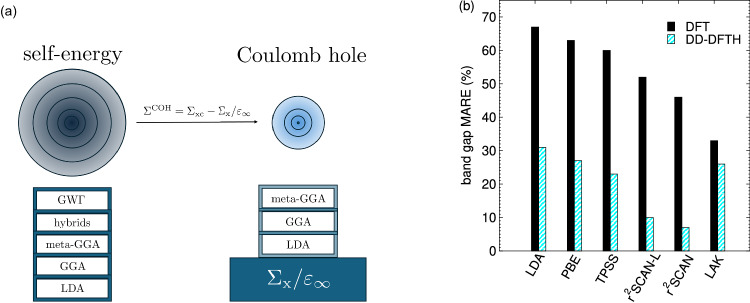
Improving DDH with a meta-GGA remainder functional. **a** Akin to Jacob’s ladder of DFT^61^, the illustration on the left side shows Jacob’s Ladder for the self-energy *Σ*_xc_. Proceeding from semilocal functionals (LDA, GGA, meta-GGA), over hybrid functionals up to many-body perturbation theory (GW and beyond), one adds more nonlocal information at each rung, improving the accuracy. The right side shows Jacob’s Ladder for the Coulomb hole (*Σ*_xc_ − *Σ*_x_/*ε*_*∞*_). The screened interaction *Σ*_x_/*ε*_*∞*_ explicitly accounts for the long-range asymptotic Coulomb interaction, making the remainder more short-ranged^17–19,38^. Semilocal DFT can more easily approximate this remainder (the Coulomb hole). **b** Band gap benchmark for various DFT functionals and their respective DD-DFTH functionals. The dark bars indicate the accuracy of the DFT base functionals, corresponding to Jacob’s Ladder for the self-energy. The light bars indicate the accuracy of the DD-DFTH functionals, corresponding to Jacob’s Ladder for the Coulomb hole. Band gap accuracy is measured by the mean absolute relative errors (MARE) over a benchmark set of 39 semiconductors and insulators; numerical data are given in Supplementary Tables 2 and 3. Source data are provided as a Source Data file.

Importantly, Eq. (11) is exact in the long-range limit $| {{{\bf{r}}}}-{{{{\bf{r}}}}}^{{\prime} }| \to \infty$^38^. Thus, one can also directly obtain Eq. (13) from Eq. (5) by imposing $\alpha={\varepsilon }_{\infty }^{-1}$ as an asymptotically exact constraint^34,37^. For finite systems such as atoms, one recovers *α* = 1. Apart from the long-range limit, the approximation in Eq. (11) is, of course, quite drastic, as real dielectric functions are far from static and dispersionless. Range-separated hybrid functionals have been derived by using more realistic model dielectric functions instead of Eq. (11)^48,50,55^. The link between DDH and GW is further blurred by the fact that ﻿GW neglects screening beyond the random-phase approximation, whereas DDH can include all screening effects to ${\varepsilon }_{\infty }^{-1}$. Nevertheless, we can draw some heuristic conclusions from the GW analogy. Namely, the Coulomb hole is identified as the remainder potential for the specific case of $\alpha={\varepsilon }_{\infty }^{-1}$ [see Eq. (12)]. This enables us to extend the ideas of Garrick et al.^60^ to the solid state. It has been specifically shown that the Coulomb hole in solids is short-ranged, as the screened exchange already takes care of the long-range asymptotic Coulomb interaction^17–19,38^. By treating the long-range part of the self-energy *Σ*_x_/*ε*_*∞*_ exactly, the remaining part of the self-energy (i.e., the Coulomb hole, *Σ*^COH^ = *Σ*_xc_ − *Σ*_x_/*ε*_*∞*_) can be much better approximated by semilocal DFT than the full self-energy *Σ*_xc_. This principle is illustrated in Fig. 1a, where we compare Jacob’s Ladder for the Coulomb hole (right) with the conventional Jacob’s Ladder (left)^61^.

### Role of the DFT base functional

Historically, the first hybrid functional proposed by Becke was based on the LDA^62^. Becke quickly proceeded to develop hybrid functionals based on GGA and meta-GGA^35,63^. Likewise, early DDH functionals used the LDA for their DFT part^40^, and GGA was rapidly adopted into DDH functionals as well^37^. However, further improvements have remained largely unexplored. Now, we proceed with a DDH functional based on meta-GGA, climbing Jacob’s Ladder^61^ for the Coulomb hole [see Fig. 1a]. A similar argument was recently presented by Jana et al.^64^. These authors also proposed a range-separated DDH based on a different meta-GGA functional. However, we note that their hybrid functional does not preserve the correct long-range limit due to their refitting of *α*^64^. The benchmark shown in Fig. 1b shows systematic improvement for the band gaps of the DFT base functionals as one proceeds from LDA to GGA (PBE), and further to meta-GGA. Out of the four meta-GGA functionals tested, the DDH based on r^2^SCAN yields the best band gaps, indicating that r^2^SCAN gives the best approximation for the remainder potential. Notably, the DDH based on the LAK^30^ (Lebeda–Aschebrock–Kümmel) meta-GGA underperforms, even though LAK yields better DFT band gaps than r^2^SCAN. In the rest of this work, we focus on the comparison of the presently proposed DFT base functional r^2^SCAN with the current standard PBE. A more detailed analysis of the other DFT base functionals and their DDH functionals is given in Supplementary Note 5.

A global hybrid functional [Eq. (5)] interpolates the band gap approximately linearly between the DFT gap and the gap obtained from Hartree–Fock (+DFT correlation). Assuming that the electronic orbitals vary little with *α*, one can obtain^37,65,66^

14 $${E}_{{{{\rm{g}}}}}^{{{{\rm{DFTH}}}}}(\alpha )\simeq {E}_{{{{\rm{g}}}}}^{{{{\rm{DFT}}}}}+\alpha ({E}_{{{{\rm{g}}}}}^{{{{\rm{HF}}}}+{{{{\rm{DFT}}}}}_{{{{\rm{c}}}}}}-{E}_{{{{\rm{g}}}}}^{{{{\rm{DFT}}}}}).$$

Strict equality in Eq. (14) holds if the DFT orbitals do not change when Fock exchange is added. It is interesting to note that this relation has an analogy in materials science. Namely, Vegard’s law states that the band gap of a binary compound semiconductor linearly interpolates between the band gaps of the two constituent semiconductors. Equation (14) was initially proven for a standard Kohn–Sham DFT base functional^37^, but following the original arguments, it is easy to see that Eq. (14) also holds for meta-GGA base functionals. For the sake of completeness, we note that the generalizations of DDH to finite systems^46^ and strongly heterogeneous systems^47^ are equally applicable to base functionals from meta-GGA.

**Fig. 2:**
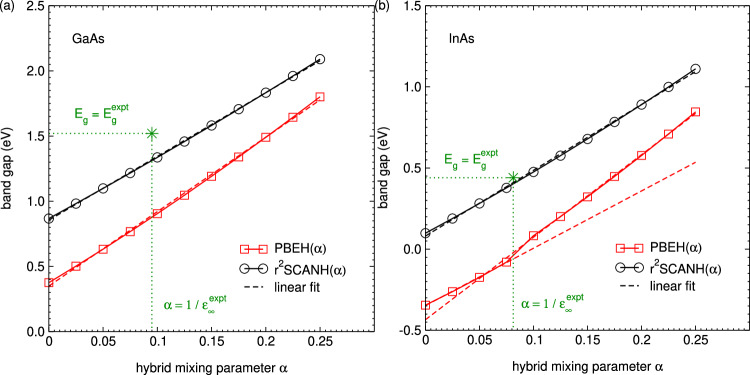
Role of the DFT base functional in the DDH band gap. Band gaps obtained with hybrid functionals PBEH and r^2^SCANH as a function of the mixing parameter *α*. **a** GaAs, both hybrid functionals closely follow the linear behavior predicted by Eq. (14) (dashed lines represent linear fits). **b** InAs, r^2^SCANH still exhibits linear behavior, but PBEH deviates from linearity as there is an inversion of valence and conduction bands for *α* ≲ 0.1, see also Table 1. Here, the two red dashed lines represent linear fits to small *α* data and large *α* data (regions of negative and positive band gaps, respectively). The fact that the experimental data (indicated by green stars) lie closer to the black lines than to the red lines signifies improvement of DD-r^2^SCANH upon DD-PBEH. Source data are provided as a Source Data file.

The accuracy of Eq. (14) for a standard semiconductor (GaAs) is demonstrated in Fig. 2a. The dependence of ${E}_{{{{\rm{g}}}}}^{{{{\rm{DFTH}}}}}$ as a function of *α* is very linear for both PBEH(*α*) and r^2^SCANH(*α*). Similar curves for different materials were recently obtained by Yang, Falletta, and Pasquarello^53^ using PBEH, confirming that Eq. (14) holds very well for typical semiconductors and insulators. We also note that PBEH underestimates the experimental band gap when the experimental inverse dielectric constant is used as a mixing parameter (see green star). Due to the larger gap of the r^2^SCAN base functional, the ideal mixing parameter for r^2^SCANH is closer to ${\varepsilon }_{\infty }^{-1,{{{\rm{expt}}}}}$.

### r^2^SCAN can open band gaps for false PBE metals

Narrow-gap semiconductors (NGS) are physically interesting materials due to their small band gaps (*E*_g_ ≲ 1 eV) and relatively large spin–orbit coupling effects. Key technological applications of NGS include spintronics, optoelectronics and thermoelectrics. In Fig. 2b, we study InAs, an important narrow-gap semiconductor. Inspecting the band gaps of the base functionals (*α* = 0), one can see that PBE yields a negative band gap. That is, PBE predicts a false metallic phase, and wrongly interchanges the order of the *p*-like valence band and the *s*-like conduction band. This is a well-known failure of PBE and occurs also for other semilocal DFT functionals such as LDA^67^. Figure 2b further shows that the negative band gap persists also for finite values of *α*, until the correct band order is finally restored for *α* ≳ 0.10. This means that PBEH can only reproduce the experimental band gap if a very large mixing parameter is used ($\alpha \approx 0.17\gg {\varepsilon }_{\infty }^{-1,{{{\rm{expt}}}}}$). It is also evident that PBEH shows a distinct non-linearity around *α* ≈ 0.09, consistent with the idea that the electronic orbitals undergo qualitative changes when the material becomes semiconducting. In contrast, the r^2^SCAN base functional already yields a finite band gap for InAs. Morevover, the band gap behavior of r^2^SCANH(*α*) is approximately linear, and the optimal mixing parameter is close to ${\varepsilon }_{\infty }^{-1,{{{\rm{expt}}}}}$. In summation, DD-r^2^SCANH exhibits the desired behavior of a dielectric-dependent hybrid functional for InAs, whereas DD-PBEH clearly does not.

InAs is not the only narrow-gap semiconductor that is a false DFT metal. Classic examples include germanium (Ge), cadmium oxide (CdO), gallium antimonide (GaSb), and indium pnictides (InX), see Table 1. Even if PBE does not predict a false metallic phase, the valence or conduction band can still be qualitatively wrong. For instance, PBE predicts small band gaps for the lead chalcogenides (PbX) listed in Table 1, but inverts valence and conduction bands if spin–orbit coupling is taken into account (the band gap should be interpreted as negative, see Supplementary Table S4 and refs. ^68,69^). Here, we find that r^2^SCAN can open band gaps for eight out of nine NGS, albeit the gaps for CdO and InN remain very small, see Supplementary Table S2. Two factors contribute to this striking improvement^10,26,70^. On the one hand, the use of a GKS scheme enables meta-GGA functionals to yield more realistic band gaps than GGA functionals (see also Supplementary Note 5). On the other hand, the fact that r^2^SCAN reduces the self-interaction error with respect to PBE also contributes to larger r^2^SCAN band gaps. However, Table 1 also shows that r^2^SCAN incorrectly yields a vanishing band gap for InSb, even though the correct band order is retained. The band gap closes only when spin–orbit coupling is taken into account, see Supplementary Table 2. In contrast, PBE yields a large negative band gap for InSb. Previously, it has also been found that SCAN and r^2^SCAN can open band gaps for other classes of false PBE metals. References ^71^ and ^72^ demonstrate this for Cu-chalcogenides, and refs. ^70,73,74^ for transition metal oxides (see also Supplementary Note 7 for FeO and CoO). A recent review further showcases these remarkable electronic structure advancements^75^. However, SCAN and r^2^SCAN cannot open any band gap, and some materials remain false metals like InSb, see also refs. ^14,31,32^.

**Table 1:** Electronic ground state of nine challenging narrow-gap semiconductors (NGS)

	Band gap		Band order	
	PBE	r^2^SCAN	PBE	r^2^SCAN
Ge	✗	*✓*	✗	*✓*
GaSb	✗	*✓*	✗	*✓*
CdO	✗	*✓*	✗	*✓*
InN	✗	*✓*	✗	*✓*
InAs	✗	*✓*	✗	*✓*
InSb	✗	✗	✗	*✓*
PbS	*✓*	*✓*	✗	*✓*
PbSe	*✓*	*✓*	✗	*✓*
PbTe	*✓*	*✓*	✗	*✓*

PBE yields wrong metallic ground states for six NGS, whereas r^2^SCAN can open a band gap for all NGS except InSb. r^2^SCAN consistently gives the correct band order for valence and conduction bands. In contrast, PBE wrongly predicts a band inversion for all these NGS.

### r^2^SCAN predicts accurate dielectric constants

**Fig. 3:**
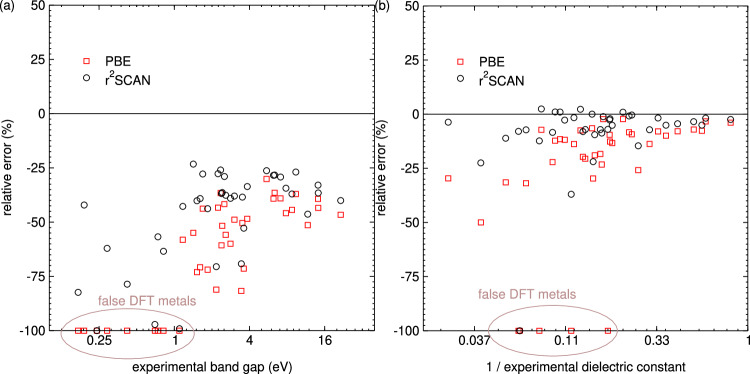
Performance of the DFT base functionals. Comparison of PBE and r^2^SCAN for **a** band gaps and **b** inverse dielectric constants. Numerical data and statistics are listed in Supplementary Tables 2 and 5. Relative errors are given with respect to the experiment, and relative errors of −100% indicate qualitatively wrong ground states (see Table 1). Note that logarithmic scales are used for the abscissae. Source data are provided as a Source Data file.

In the following, we compare the electronic structure predicted by PBE and r^2^SCAN for a wider set of materials. Our benchmark set consists of 39 well-characterized materials and covers a large range of band gaps and dielectric constants, ranging from PbTe (narrow-gap semiconductor) to Ne (wide-gap insulator). To ensure a direct, unbiased comparison, we use the experimental geometries as given in Supplementary Table 1. Figure 3a shows that PBE substantially underestimates the experimental band gaps with a MARE of 62% (mean absolute relative error). This is the aforementioned band gap problem of DFT^16^. Band gaps predicted by r^2^SCAN are slightly larger, but still underestimate the experimental gaps (MARE 46%). This trend is in good agreement with the high-throughput study of ref. ^31^, who reported MAREs of 46% for PBE and 38% for SCAN, respectively. As we demonstrate below, the addition of Fock exchange enables both PBE and r^2^SCAN to obtain realistic band gaps.

Within DDH, the mixing parameter *α* is given by the inverse dielectric constant [Eq. (13)]. In Fig. 3b, we therefore compare ${\varepsilon }_{\infty }^{-1}$ predicted by PBE and r^2^SCAN with experiment. We use finite electric fields^76,77^ to calculate ${\varepsilon }_{\infty }^{-1}$, see Supplementary Note 1 for further details. Inverse dielectric constants predicted by PBE are systematically smaller than experimental ones (MARE 26%). In comparison, inverse dielectric constants predicted by r^2^SCAN are astoundingly accurate (MARE 9%). Thus, r^2^SCAN largely eliminates the PBE overscreening error. We can attribute this success to the reduction of the self-interaction error inherent to PBE. That is, semilocal functionals such as PBE yield ground state charge densities that are too delocalized^22,24,75^. Thus, the response of the overly spread-out charge density to an electric field is exaggerated, resulting in overscreening. Heuristically speaking, semilocal density functionals are based on electron gas models, and therefore, it is not surprising that these functionals tend to deviate in the direction of excessively metal-like response (overscreening). It has been shown explicitly that the r^2^SCAN charge densities are more compact, indicating reduced self-interaction error^75^. To this point, recent studies have also found that SCAN and r^2^SCAN can achieve impressive improvements for Born effective charges^12,78,79^, electron-phonon coupling^79^, molecular polarizabilities^80^ and chemical shifts^81^. Figure 3b also shows that an accurate description of band gaps is not always necessary for accurate dielectric constants. This can be understood by the fact that the static dielectric constant is a ground state property, see refs. ^82–84^. Thus, even the standard KS method can, in principle, predict ${\varepsilon }_{\infty }^{-1}$ correctly, whereas the band gap is outside of standard KS theory [compare the relative errors obtained by PBE in Fig. 3a, b]. The differences between r^2^SCAN and PBE are clearly correlated with ${\varepsilon }_{\infty }^{-1}$. That is, r^2^SCAN mostly improves ${\varepsilon }_{\infty }^{-1}$ for semiconductors (${\varepsilon }_{\infty }^{-1,{{{\rm{expt}}}}}\lesssim 0.25$), whereas the calculated inverse dielectric constants for insulators are very similar (${\varepsilon }_{\infty }^{-1,{{{\rm{expt}}}}}\gtrsim 0.25$). The same trend is also observed for the band gaps (see also ref. ^31^), but not as clearly as for ${\varepsilon }_{\infty }^{-1}$.

### DD-r^2^SCANH, a dielectric-dependent hybrid functional based on r^2^SCAN

We now investigate dielectric-dependent hybrid functionals based on PBE and r^2^SCAN. For a fair comparison, we first use the experimental inverse dielectric constants listed in Supplementary Table 5 as mixing parameter [$\alpha={\varepsilon }_{\infty }^{-1,{{{\rm{expt}}}}}$ in Eq. (13)]. Next, ab initio choices for *α* will be discussed.

**Fig. 4:**
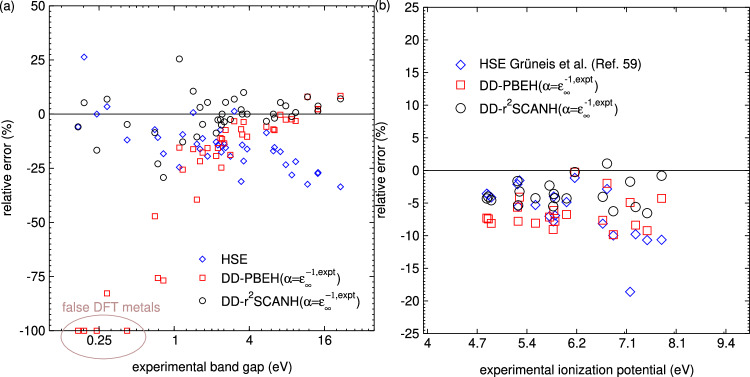
Benchmarking DD-r^2^SCANH for energy levels. **a** Band gaps and **b** ionization potentials predicted by dielectric-dependent hybrid functionals based on PBE and r^2^SCAN. Inverse dielectric constants from experiment are used as mixing parameters ($\alpha={\varepsilon }_{\infty }^{-1,{{{\rm{expt}}}}}$, see text). For reference, we also show results obtained with the HSE functional^85^. Relative errors of −100% indicate qualitatively wrong ground states for DD-PBEH, see also Supplementary Table 2. Ionization potentials of dielectric-dependent hybrid functionals are calculated by combining bulk valence band shifts with HSE surface lineups from ref. ^59^. For further details, see Supplementary Note 2. Note that logarithmic scales are used for the abscissae. Source data are provided as a Source Data file.

Figure 4a compares the calculated hybrid functional band gaps with the experiment. It is clear that DD-PBEH consistently underestimates semiconductor band gaps (${E}_{{{{\rm{g}}}}}^{{{{\rm{expt}}}}}\lesssim$ 5 eV), confirming the findings of refs. ^45,48,50^. DD-PBEH even yields a false metallic ground state for some NGS [${E}_{{{{\rm{g}}}}}^{{{{\rm{expt}}}}}\lesssim$ 1 eV, see also Fig. 2b]. In comparison, DD-r^2^SCANH closely reproduces the experimental band gaps for all NGS, and significantly improves the accuracy for standard semiconductors as well. For insulators (${E}_{{{{\rm{g}}}}}^{{{{\rm{expt}}}}}\gtrsim 5\,{\mbox{eV}}\,$), DD-PBEH and DD-r^2^SCANH yield very similar results, both slightly overestimating the experimental band gaps. For comparison, Fig. 4a also shows results obtained with the popular HSE (Heyd–Scuseria–Ernzerhof) functional^85–88^. HSE works well for semiconductors, including NGS, but notably underestimates the band gaps of insulators. This can be understood by the fact that the fixed *α* = 1/4 used in HSE is too small for insulators^41^.

Overall, DD-r^2^SCANH achieves the best accuracy for this band gap benchmark (MARE 7%), followed by HSE (MARE 16%) and DD-PBEH (MARE 27%). In Supplementary Table 3, we also list band gaps obtained via a high-level many-body perturbation technique (GWΓ, calculations done by ref. ^59^). This method achieves a MARE of 6% for a subset of 24 materials, close to the accuracy of DD-r^2^SCANH (7% for the same subset, same as the MARE for the full 39 materials). It is also interesting to compare the performance of DD-r^2^SCANH with range-separated hybrid functionals. The doubly screened hybrid (DSH) of ref. ^48^ achieves a MARE of 14% for a subset of 30 materials (band gaps listed in Supplementary Table 3, the DD-r^2^SCANH MARE for the same subset is 6%, similar to the 7% for the full 39 materials. Previous studies have shown that the range-separated hybrid of Chen et al. (DD-RSH-CAM) performs similarly to DSH^50,53^. Moreover, the range-separated hybrid of refs. ^54,89^ is essentially equivalent to DD-RSH-CAM for conventional semiconductors and insulators as considered here (see, however, Supplementary Note 7 for a discussion of the strongly correlated material NiO). Recently, accurate band gaps were also obtained with the WOT-SRSH range-separated hybrid functional from ref. ^52,90^. WOT-SRSH yields a MARE of 7% for a subset of 15 materials (band gaps listed in Supplementary Table 3), compared to 9% MARE achieved by DD-r^2^SCANH for this subset. However, we note that the optimal tuning procedure for WOT-SRSH is fairly involved, and the range-separation parameter *γ* is ill-defined for materials such as AlN that have ${\varepsilon }_{\infty }^{-1}\approx 1/4$ (“*γ* collapse problem”). Finally, the range-separated hybrid functional based on meta-GGA of ref. ^64^ performs slightly worse than DD-RSH-CAM, even though it employs fitted parameters to optimize band gap accuracy.

While the performance of DD-r^2^SCANH for band gaps is encouraging, it is important to verify that combining the DDH scheme with r^2^SCAN does not spoil other electronic structure properties. As we will see, this is not an issue, and DD-r^2^SCANH consistently inherits the improvements from its r^2^SCAN base functional. First, we calculate ionization potentials (IPs) for a subset of 20 semiconductors and insulators by combining bulk valence band shifts with surface lineups from ref. ^59^ (see Supplementary Note 2 for details). The IP is a ground state property and thus in principle accessible to standard Kohn–Sham DFT^20,21,34^. Nevertheless, it is well known that PBE systematically underestimates experimental IPs^66,91–93^, mirroring its description of band gaps and inverse dielectric constants. Here, we obtain a MARE of 16% for PBE, see Supplementary Table 6. Consistent with ref. ^93^, we find that r^2^SCAN IPs (MARE 12%) only slightly improve upon PBE and remain underestimated. The addition of Fock exchange systematically increases the ionization potentials. This follows from the fact that Eq. (14) holds not only for band gaps, but separately for valence band edges and conduction band edges^37,65,66^. Figure 4b shows that DD-r^2^SCANH (MARE 4%) yields slightly larger ionization potentials than DD-PBEH (MARE 7%), resulting in better agreement with experiment. DD-r^2^SCANH also surpasses HSE (MARE 6%), and its accuracy for ionization potentials approaches the level of GWΓ^59^ (MARE 3%, see Supplementary Table 6). We conclude that the improved starting point provided by the r^2^SCAN base functional is helpful not only for band gaps, but also for ionization potentials.

Similarly, we also find that DD-r^2^SCANH yields effective masses of six common semiconductors in good agreement with the experimental ones, see Table 2. In contrast, effective masses obtained by DD-PBEH are too small, reflecting the underestimated band gaps. Indeed, effective masses are roughly proportional to band gaps and also increase with the exchange mixing parameter *α*^94^.

**Table 2:** Effective masses obtained using Eq. (16)

	PBE	r^2^SCAN	HSE	DD-PBEH	DD-r^2^SCANH
MSRE	−32%	−32%	−5%	−29%	−6%
MARE	53%	32%	11%	44%	13%

The data set includes 24 hole and electron effective masses, see Supplementary Table 7. Mean signed relative errors (MSRE) and mean absolute relative errors (MSRE) compare different exchange-correlation functionals with experimental data. Dielectric-dependent hybrid functionals use inverse dielectric constants from experiment for the *α* parameter in Eq. (13). Numerical data and further details are given in Supplementary Table 7.

Finally, we assess the DD-r^2^SCANH equilibrium lattice constants for 18 representative strongly bonded solids. PBE has a well-known tendency of overestimating lattice constants (underbinding), and the fact that r^2^SCAN largely eliminates this underbinding is an important achievement^13,14^. Table 3 shows that DD-PBEH tends to correct the PBE underbinding error, but for InAs and InSb, the DD-PBEH lattice constants remain significantly too large (about 2%, see Supplementary Table 8). Finally, DD-r^2^SCANH keeps the accurate geometries obtained by r^2^SCAN (similar MARE), and even gets rid of the small systematic underbinding of r^2^SCAN (the MSRE improves from +0.5 to +0.1%).

**Table 3:** Equilibrium lattice constants obtained using Birch–Murnaghan equation of state fits [Eq. (17)]

	PBE	r^2^SCAN	HSE	DD-PBEH	DD-r^2^SCANH
MSRE	+1.6%	+0.5%	+0.6%	+0.7%	+0.1%
MARE	1.6%	0.5%	0.7%	0.8%	0.4%

The data set includes 18 strongly bonded solids. Mean signed relative errors (MSRE) and mean absolute relative errors (MARE) compare different exchange-correlation functionals with experimental data. Dielectric-dependent hybrid functionals use experimental inverse dielectric constants for the *α* parameter in Eq. (13). Numerical data and further details are given in Supplementary Table 8.

### Non-empirical hybrid functional based on r^2^SCAN

Up to now, we have used the experimental inverse dielectric constants as mixing parameters. This is practicable here, since our benchmark materials are well characterized. However, accurate values for ${\varepsilon }_{\infty }^{-1,{{{\rm{expt}}}}}$ are not always available. From a theoretical standpoint, it is also more satisfying to stay free from empirical parameters. In Table 4, we compare different first principles choices for *α*, and assess their performance for our band gap benchmark. First, a fixed mixing parameter *α* = 1/4 yields 44% MARE for PBE0, better than the 63% for PBE. In contrast, *α* = 1/4 gives a MARE of 64% for r^2^SCAN0, worse than the MARE of 46% of the r^2^SCAN base functional. Supplementary Fig. 1 shows that the reason for this poor performance is that PBE0 and especially r^2^SCAN0 significantly overestimate the band gaps of semiconductors (particularly NGS). PBE0 profits from fortuitous error cancellation, as the PBE induced overscreening compensates for the overly large amount of Fock exchange (see Fig. 2). We note that the mean absolute errors of both PBE0 (MAE 0.79 eV) and r^2^SCAN0 (MAE 0.84 eV) do show improvements with respect to the respective base functionals, and further outperform HSE for this metric (MAE 0.94 eV), see Table 4. This reflects the comparatively better description of insulators achieved by both PBE0 and r^2^SCAN0. Nevertheless, large mean absolute errors persist, and r^2^SCAN0 remains, on average, worse than PBE0. Previously, it has also been reported that SCAN0 cannot outperform PBE0 for ionization potentials and electron affinities of small molecules^56^, while r^2^SCAN0 gives similar results to SCAN0^57^. We conclude that to take full advantage of the improved meta-GGA base functional, one has to abandon the fixed screening parameter *α* = 1/4.

**Table 4:** Band gaps predicted by various hybrid functionals based on PBE and r^2^SCAN

		MARE	MAE
PBE	*α* = 0	63%	2.07 eV
HSE	*α* = 1/4^*^	16%	0.94 eV
PBE0	*α* = 1/4	44%	0.79 eV
DD-PBEH	$\alpha=1/{\varepsilon }_{\infty }^{{{{\rm{PBE}}}}}$	35%	0.50 eV
DD-PBEH	$\alpha=1/{\varepsilon }_{\infty }^{{{{\rm{expt}}}}}$	27%	0.37 eV
r^2^SCAN	*α* = 0	46%	1.60 eV
r^2^SCAN0	*α* = 1/4	64%	0.84 eV
DD-r^2^SCANH	$\alpha=1/{\varepsilon }_{\infty }^{{{{{\rm{r}}}}}^{2}{{{\rm{SCAN}}}}}$	10%	0.18 eV
DD-r^2^SCANH	$\alpha=1/{\varepsilon }_{\infty }^{{{{\rm{expt}}}}}$	7%	0.19 eV
*HSE is a range-separated variant of PBE0 that uses *α* = 1/4 in the short-range and *α* = 0 in the long-range, see ref. ^85^.			

The hybrid functionals differ in the choice of the hybrid mixing parameter [Eq. (13)]. Band gaps for *α* = 1/4 and $\alpha=1/{\varepsilon }_{\infty }^{{{{\rm{DFT}}}}}$ are mostly interpolated via Eq. (14), though we performed explicit calculations for all NGS. The data set includes 39 standard semiconductors and insulators, as in Fig. 4a. Mean absolute relative errors (MARE) and mean absolute errors (MAE) compare calculated band gaps with experimental data. Data are visualized in Supplementary Fig. 1.

Two natural non-empirical choices are available for dielectric-dependent hybrid functionals^44^. First, one can calculate ${\varepsilon }_{\infty }^{-1}$ at the level of the DFT base functional. This choice is appealing due to its simplicity and relative numerical efficiency. Table 4 shows that for DD-r^2^SCANH, the usage of $\alpha={\varepsilon }_{\infty }^{-1,{{{{\rm{r}}}}}^{2}{{{\rm{SCAN}}}}}$ gives very similar results to $\alpha={\varepsilon }_{\infty }^{-1,{{{\rm{expt}}}}}$. This is not surprising, since the r^2^SCAN inverse dielectric constants closely approximate the experimental ones [see Fig. 3b]. In contrast, $\alpha={\varepsilon }_{\infty }^{-1,{{{\rm{PBE}}}}}$ performs worse for DD-PBEH than experimental mixing parameters. Using $\alpha={\varepsilon }_{\infty }^{-1,{{{\rm{expt}}}}}$, DD-PBEH already underestimates the band gaps of semiconductors [see Fig. 4a]. Thus, replacing ${\varepsilon }_{\infty }^{-1,{{{\rm{expt}}}}}$ with ${\varepsilon }_{\infty }^{-1,{{{\rm{PBE}}}}}$ introduces additional overscreening, and the semiconductor band gaps are underestimated even more (see Supplementary Fig. 1). Still, DD-PBEH with a material-specific screening parameter $\alpha={\varepsilon }_{\infty }^{-1,{{{\rm{PBE}}}}}$ outperforms PBE0 on average, in agreement with previous studies^45,48,53^.

The second natural ab initio choice for *α* is to calculate ${\varepsilon }_{\infty }^{-1}$ using the dielectric-dependent hybrid functionals themselves, and iterate this process to self-consistency^44^. This choice for *α* is, in principle, the most sound one, but will not be pursued here as the self-consistency cycle introduces considerable computational overhead. We also note that previous studies reported that the changes due to the self-consistency cycle are typically small^44,48,50^. We confirm this behavior for DD-r^2^SCANH for the example of GaAs, see Supplementary Note 8. References ^48^ and ^49^ have shown that the DD-PBEH band gaps of NGS remain much too small or vanish completely - even when *α* is calculated self-consistently. In Supplementary Note 8, we study the case of InSb, which is the only material in Table 1 where r^2^SCAN yields a false metallic ground state. We find that self-consistent DD-r^2^SCANH can solve this particular problem, correctly yielding a semiconducting ground state for InSb. In contrast, self-consistent DD-PBEH does not change the false metallic ground state found with PBE.

## Discussion

Throughout this work, we have demonstrated that the r^2^SCAN meta-GGA functional consistently improves electronic structure properties with respect to the semilocal PBE functional. In particular, we have found that the inverse dielectric constants obtained with r^2^SCAN are very close to experiment (MARE of 9%, compared with 26% for PBE). This is an important result on its own, qualifying r^2^SCAN as an excellent choice for high-throughput screening of dielectric constants.

**Fig. 5:**
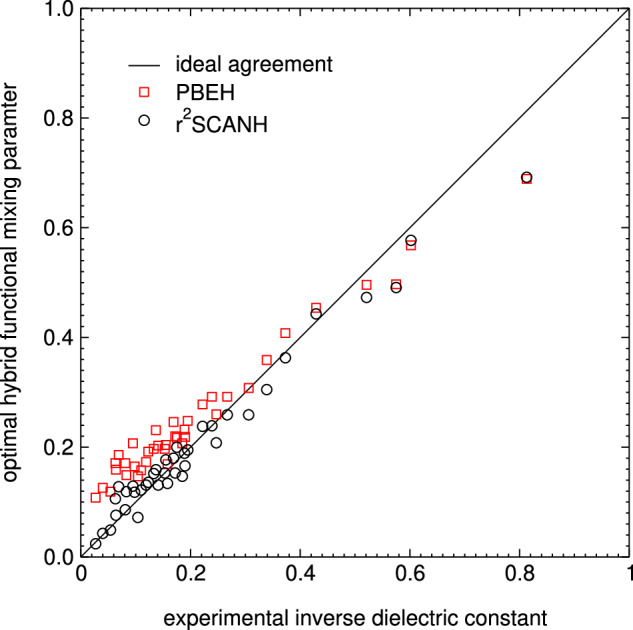
The DDH scheme is close to optimal for the r^2^SCAN base functional. The optimal mixing parameters *α*_opt_ for hybrid functionals based on PBE and r^2^SCAN are defined by requiring that the hybrid functionals reproduce the experimental band gap, ${E}_{{{{\rm{g}}}}}^{{{{\rm{DFTH}}}}}({\alpha }_{{{{\rm{opt}}}}}) {=}^{!}{E}_{{{{\rm{g}}}}}^{{{{\rm{expt}}}}}$. We calculate *α*_opt_ with the help of Eq. (14). Ideal agreement of the optimal mixing parameter with the experimental inverse dielectric constants (${\alpha }_{{{{\rm{opt}}}}}={\varepsilon }_{\infty }^{-1,{{{\rm{expt}}}}}$) indicates that the dielectric-dependent hybrid functional scheme exactly yields the experimental band gaps. Close to optimal agreement is found for r^2^SCANH, corresponding to the good performance of DD-r^2^SCANH shown in Fig. 4a. In contrast, for PBEH we find that *α*_opt_ is systematically larger than ${\varepsilon }_{\infty }^{-1,{{{\rm{expt}}}}}$ for semiconductors (${\varepsilon }_{\infty }^{-1,{{{\rm{expt}}}}}\lesssim 0.25$), corresponding to DD-PBEH underestimating the band gap of these materials [see Fig. 4a]. Source data are provided as a Source Data file.

Next, we have introduced DD-r^2^SCANH, a dielectric-dependent hybrid functional based on the r^2^SCAN meta-GGA functional. We have shown that DD-r^2^SCANH can outperform the standard dielectric-dependent hybrid functional based on PBE. In particular, the r^2^SCAN base functional significantly improves the description of narrow-gap semiconductors such as Ge and InAs. For these materials, PBE predicts false metallic ground states, which relate to strongly underestimated DD-PBEH band gaps. In other words, imposing the asymptotic constraint $\alpha={\varepsilon }_{\infty }^{-1}$ is not compatible with correct band gaps for a global hybrid functional based on PBE. Figure 5 demonstrates that the optimal mixing parameters that reproduce the experimental band gaps are consistently larger than the inverse dielectric constants (see red squares). This problem is cured by replacing the PBE base functional with r^2^SCAN (the black circles in Fig. 5 are close to the ideal curve). The accuracy of DD-r^2^SCANH approaches that of the gold-standard many-body perturbation theory techniques (GWΓ) for both band gaps and ionization potentials. It is worth noting that DD-r^2^SCANH is significantly more efficient than GWΓ in terms of computational cost. In fact, the computational cost of DD-r^2^SCANH is essentially equivalent to that of DD-PBEH for typical solid-state applications as considered in this work. The overhead of using r^2^SCAN instead of PBE for the base functional part (roughly a factor three^14^) can be neglected, as the Fock exchange part dominates the overall cost of the hybrid functional calculation. To further reduce the computational cost of DD-r^2^SCANH, our method could be extended towards range-separation as in HSE^85,86^. However, we do not pursue this approach here, since the range-separation comes at the cost of introducing an additional hybrid functional parameter and also violates the asymptotically exact constraint $\alpha={\varepsilon }_{\infty }^{-1}$ for the long-range interaction.

It is astounding that DD-r^2^SCANH, a global hybrid functional based on a single parameter, can be this accurate. In the following, we investigate the effect of the DFT base functional, and particularly the role of the exchange and correlation parts of the DFT remainder. We start the analysis with Eq. (14). In essence, the band gap of a global hybrid functional is determined by three factors: (i) the intercept of the linear function given by the band gap of the DFT base functional, (ii) the mixing parameter *α*, (iii) the band gaps slopes,

15 $$\frac{\,{{\mbox{d}}}\,{E}_{{{{\rm{g}}}}}^{{{{\rm{DFTH}}}}}}{\,{\mbox{d}}\,\alpha }(\alpha )\simeq {E}_{{{{\rm{g}}}}}^{{{{\rm{HF}}}}+{{{{\rm{DFT}}}}}_{{{{\rm{c}}}}}}-{E}_{{{{\rm{g}}}}}^{{{{\rm{DFT}}}}}.$$

**Fig. 6:**
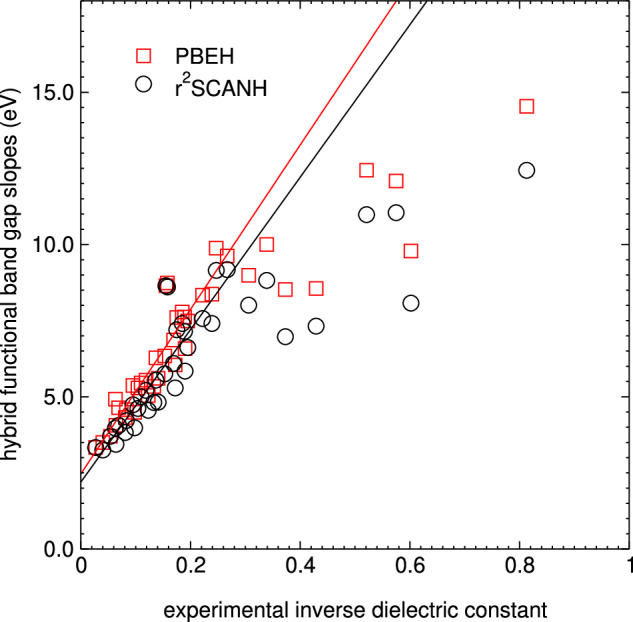
r^2^SCANH has systematically smaller band gap slopes than PBEH. Shown are the hybrid functional band gap slopes $\,{\mbox{d}}\,{E}_{{{{\rm{g}}}}}^{{{{\rm{DFTH}}}}}/\,{\mbox{d}}\,\alpha$ [see Eq. (15)] as a function of the experimental inverse dielectric constants ${\varepsilon }_{\infty }^{-1,{{{\rm{expt}}}}}$. The smaller slopes for r^2^SCANH compared to PBEH indicate that the exchange part of the r^2^SCAN base functional better approximates Fock exchange. Straight lines present linear fits to semiconductor data (${\varepsilon }_{\infty }^{-1,{{{\rm{expt}}}}}\le 0.25$). The good correlation with ${\varepsilon }_{\infty }^{-1,{{{\rm{expt}}}}}$ is further discussed in Supplementary Note 6. The slopes are calculated as $[{E}_{{{{\rm{g}}}}}^{{{{\rm{DFTH}}}}}(\alpha={\varepsilon }_{\infty }^{-1,{{{\rm{expt}}}}})-{E}_{{{{\rm{g}}}}}^{{{{\rm{DFT}}}}}]/{\varepsilon }_{\infty }^{-1,{{{\rm{expt}}}}}$. Source data are provided as a Source Data file.

We have already studied the influence of the mixing parameter, see Table 4. In Fig. 4a, the band gaps are calculated with the same mixing parameter ($\alpha={\varepsilon }_{\infty }^{-1,{{{\rm{expt}}}}}$) for both DD-PBEH and DD-r^2^SCANH. This means that the screened exchange part including the correct asymptotic Coulomb interaction is treated exactly [disregarding small differences due to electronic self-consistency, in line with Eq. (14)]. We also note that the comparison with experimental data in Fig. 4a formally assumes that all relevant physics is included in our calculations (see Supplementary Notes 2 and 3 for our treatment of spin–orbit coupling and zero-point renormalization effects). Since the band gaps obtained by DD-r^2^SCANH are better than the DD-PBEH band gaps, we can thus conclude that the remainder potential is better described by r^2^SCAN than it is by PBE [see discussion following Eq. (13)]. Next, Fig. 6 shows that the band gap slopes of r^2^SCANH are consistently smaller than the band gap slopes of PBEH. Since DFT correlation cancels out in Eq. (15), the slopes measure how different DFT exchange is from Fock exchange. Therefore, Fig. 6 tells us that r^2^SCAN exchange is closer to Fock exchange than PBE exchange. It is remarkable that the band gap slopes correlate very well with the inverse dielectric constants^37,39^ (almost linear relations for ${\varepsilon }_{\infty }^{-1,{{{\rm{expt}}}}}\lesssim 0.25$). This is discussed further in Supplementary Note 6. We note that the smaller slopes for DD-r^2^SCANH are also very convenient in practical applications. That is, the band gaps simply depend less on *α*, and the use of an approximate mixing parameter such as ${\varepsilon }_{\infty }^{-1,{{{{\rm{r}}}}}^{2}{{{\rm{SCAN}}}}}$ causes smaller errors. This is also useful for the modeling of semiconductor interfaces, where the optimal mixing parameter can be approximated by averaging the inverse dielectric constants of the two constituent semiconductors^37^.

So far, we have considered equal mixing parameters for DD-r^2^SCANH and DD-PBEH, and we have shown that the slopes are smaller for DD-r^2^SCANH. Thus, we conclude that the larger band gaps of DD-r^2^SCANH compared to DD-PBEH originate from the larger intercepts [the band gaps of the r^2^SCAN base functional are larger than PBE band gaps, see Fig. 3a]. While the slopes are only determined by exchange, correlation plays an important role for the intercepts^29,32^. Supplementary Fig. 2 shows that the more nonlocal correlation functional in r^2^SCAN, compared to PBE, generally reduces the band gaps, partially counteracting the effect of the more nonlocal exchange functional.

Summing up the analysis so far, we see that r^2^SCAN improves upon PBE not only in terms of exchange and correlation, but also notably in terms of the remainder potential [Eq. (4), see also Supplementary Fig. 5]. The improved DFT band gaps [Fig. 3a] represent a step up Jacob’s Ladder for the self-energy *Σ*_xc_ (left side of Fig. 1). The improvement is systematic, and in cases like InAs even qualitative. Still, r^2^SCAN alone is clearly an insufficiently accurate approximation to *Σ*_xc_. Once we remove the asymptotic long-range Coulomb interaction by treating *Σ*_x_/*ε*_*∞*_ exactly, the remaining part becomes more short-ranged. In a GW picture, this remainder is identified as the Coulomb hole [see Eq. (12)], and it is much easier to approximate by semilocal DFT than the fully nonlocal self-energy *Σ*_xc_ (see right side of Fig. 1). This brings new life to Kohn’s principle of near-sightedness^95^, pinpointing the Coulomb hole as the central semilocal quantity that should be approximated by DFT. The fact that r^2^SCAN works this well across a wide range of band gaps and dielectric constants is extremely encouraging.

In conclusion, we advance dielectric-dependent hybrid functional theory by replacing the PBE part of the hybrid functional with r^2^SCAN. This solves a long-standing problem of the dielectric-dependent hybrid functional. Namely, DD-PBEH underestimates the band gaps of semiconductors and especially of narrow-gap semiconductors. Our DD-r^2^SCANH functional improves the band gap accuracy and can treat narrow-gap semiconductors on the same footing as standard materials. The improvements of DD-r^2^SCANH also extend to other electronic structure properties. DD-r^2^SCANH keeps the simple and elegant one-parameter global hybrid form, and can be readily adopted by the broader electronic structure community. Moreover, the computational overhead of DD-r^2^SCANH compared to the standard DD-PBEH is fairly modest for typical solid-state calculations.

## Methods

All electronic structure calculations are performed using the projector augmented-wave (PAW) code VASP^96,97^. We use accurate PBE PAW pseudopotentials^97^ distributed with vasp.6.4.2 that include semi-core electrons as valence, details are given in Supplementary Table 1. We raise the default plane-wave cutoffs (ENCUT in VASP) by a factor of 1.4 to ensure that band gaps are converged within 0.01 eV with respect to this parameter. Furthermore, we use 12 × 12 × 12 *Γ*-centered **k**-point meshes, ensuring convergence up to 0.01 eV as well. For wurtzite and rutile materials, we reduce the number of **k**-points along the *c* direction according to the *c*/*a* ratio. Dielectric constants are calculated using finite electric fields^76,77^, details are given in Supplementary Note 1. We account for the fact that denser **k**-point samplings are required for the calculation of dielectric constants^98^. Throughout, the calculations are performed at the experimental geometries given in Supplementary Table 1, except for the determination of equilibrium lattice constants (Table 3). Our calculations account for spin–orbit coupling (SOC) and zero-point renormalization (ZPR) effects; details are given in Supplementary Notes 3 and 4, respectively.

To obtain effective masses, we follow the procedure of ref. ^99^. Band structures are evaluated with dense **k**-point samplings along specified directions in reciprocal space, and effective masses m^*^ are obtained by fitting to the non-linear ansatz

16 $$E(k)\left[1+E(k)/{E}_{0}\right]={\hslash }^{2}{k}^{2}/2{m}^{*}.$$

Here, *E*_0_ is a fit parameter and accounts for band nonparabolicity^100^. Equilibrium lattice constants are obtained by fitting energy-volume curves to a four-parameter Birch–Murnaghan equation of state,^101^

17 $$E(V)=a+b{V}^{-2/3}+c{V}^{-4/3}+d{V}^{-2}.$$

DD-r^2^SCANH is available as of version vasp.6.4, including structure relaxation. Since DDH functionals and other optimally-tuned hybrid functionals are not size consistent^33,45^, comparing total energies for materials with different hybrid functional parameters is problematic. We therefore advice to select a single parameter $\alpha={\varepsilon }_{\infty }^{-1}$ for a given material, and keep it fixed. For the study of semiconductor interfaces, we recommend the mixed scheme described in ref. ^37^.

## Supplementary information

Supplementary Information

Transparent Peer Review File

## Source data

Source Data

## Data Availability

The benchmark data generated in this study are provided in the Supplementary Information. Crystal structure files are freely available on the Materials Cloud platform, see ref. ^102^. Source data are provided with this paper.

## Code availability

The calculations are performed mostly with vasp.6.4.2. DD-r^2^SCANH is fully available in VASP as of version vasp.6.4, including structure relaxation. LAK calculations are performed with vasp.6.5.0. VASP input files are freely available on the Materials Cloud platform, see ref. ^102^.
