# Supplementary material for: Dielectric-dependent hybrid functional based on meta-GGA
Source: Nat Commun. 2026 Jul 4;17:8323. doi: 10.1038/s41467-026-75146-x (PMC13470121; doi:10.1038/s41467-026-75146-x)
Supplement: Supplementary file 1 [file 41467_2026_75146_MOESM1_ESM.pdf]

Supplementary Information for Dielectric-dependent hybrid functional based on meta-GGA

Stefan Riemelmoser*,[1] Xun Xu,[1,2] and Alfredo Pasquarello[1]
[1] *Chaire de Simulation à l'Echelle Atomique (CSEA), Ecole Polytechnique Fédérale de Lausanne (EPFL), CH-1015 Lausanne, Switzerland*
[2] *Beijing Computational Science Research Center, Beijing 100193, China*

*Corresponding author email: stefan.riemelmoser@epfl.ch

SUPPLEMENTARY NOTE 1 - CALCULATION OF DIELECTRIC CONSTANTS

In the following, we describe the calculation of static, ion-clamped, macroscopic dielectric constants ε_∞ using finite electric fields.[1,2] Specifically, we use an implementation along the lines of the "perturbation expansion after discretization" (PEAD) method of Nunes and Gonze.[3] This involves minimizing the electric enthalpy functional,

$$E[\boldsymbol{\mathcal{E}}, u_{n\mathbf{k}}] = E^{\mathrm{KS}}[u_{n\mathbf{k}}] - \Omega \boldsymbol{\mathcal{E}} \cdot \boldsymbol{\mathcal{P}}[u_{n\mathbf{k}}], \tag{1}$$

with respect to $u_{n\mathbf{k}}$. Here, $u_{n\mathbf{k}}$ are the cell-periodic parts of the occupied electronic orbitals, $\boldsymbol{\mathcal{E}}$ is the electric field, $\boldsymbol{\mathcal{P}}$ is the polarization and Ω is the unit cell volume. The component of $\boldsymbol{\mathcal{P}}$ along a reciprocal lattice vector, $\boldsymbol{\mathcal{P}} \cdot \mathbf{b}_i$, is calculated via the discretized Berry phase expression[4]

$$\boldsymbol{\mathcal{P}} \cdot \mathbf{b}_i = \frac{2e}{\Omega} \frac{1}{N_\perp^{(i)}} \sum_{l=1}^{N^{(i)}} \mathrm{Im} \ln \prod_{j=0}^{N^{(i)}-1} \det S(\mathbf{k}_j, \mathbf{k}_{j+1}). \tag{2}$$

Here, e is the unit charge, and the factor two accounts for spin. Furthermore, the Brillouin zone is sampled with $N_\mathbf{k} = N^{(i)} N_\perp^{(i)}$ $\mathbf{k}$-points ($N^{(i)}$ along $\mathbf{b}_i$, and $N_\perp^{(i)}$ in the plane perpendicular to $\mathbf{b}_i$). Moreover, S is the overlap matrix given by $S_{mn}(\mathbf{k}, \mathbf{k}') = \langle u_{m\mathbf{k}} | u_{n\mathbf{k}'} \rangle$. Then, the dielectric tensor $\varepsilon_{\infty,\alpha\beta}$ can be obtained as

$$\varepsilon_{\infty,\alpha\beta} = \delta_{\alpha\beta} + 4\pi \frac{\partial \boldsymbol{\mathcal{P}}_\alpha}{\partial \boldsymbol{\mathcal{E}}_\beta}, \tag{3}$$

and the dielectric constant is calculated as the trace average

$$\varepsilon_\infty = \frac{\varepsilon_{\infty,xx} + \varepsilon_{\infty,yy} + \varepsilon_{\infty,zz}}{3}. \tag{4}$$

The PEAD method holds several advantages: (i) it naturally includes all screening effects for arbitrary exchange-correlation functionals (including functionals in generalized Kohn-Sham DFT[5]). That is, the DFT exchange-correlation kernel is self-consistently taken into account. The importance of the exchange-correlation kernel for dielectric-dependent hybrid functionals was stressed in Refs. 6 and 7. (ii) Calculations can be done in a single supercell unlike earlier methods based on finite electric fields.[8,9] (iii) The method does not rely on Wannier functions as opposed to earlier works.[10,11] For details of the implementation, we refer to Refs. 1–3, and for theoretical background ("modern theory of polarization") we recommend the seminal papers of King-Smith and Vanderbilt [12] and Resta.[4]

To check the accuracy of our calculations, we compare results obtained via the PEAD method with density functional perturbation theory (DFPT, see Ref. 13). Supplementary Table 5 shows that PBE dielectric constants obtained with both methods closely agree, as they should.[3,4] Our dielectric constants obtained with PBE are also in good agreement with previous studies.[6,14] It is satisfying that the numerical differences are much smaller than typical experimental errors ($|\varepsilon_\infty^{-1,\mathrm{PEAD}} - \varepsilon_\infty^{-1,\mathrm{DFPT}}| \leq 0.001$). To obtain this level of accuracy, it is important to carefully converge the electronic wave functions to high precision in both methods. Further, the finite electric field $\boldsymbol{\mathcal{E}}$ needs to be chosen small enough to ensure that the dielectric response stays within the linear regime. Here, we use a uniform electric field vector of $\boldsymbol{\mathcal{E}} = \{\mathcal{E}, \mathcal{E}, \mathcal{E}\}$ with $\mathcal{E} = 0.003$ eV Å^{-1} for all materials (`EFIELD_PEAD` in `VASP` [see Eq. (1)].

SUPPLEMENTARY NOTE 2 - CALCULATION OF IONIZATION POTENTIALS

The ionization potential is defined as the energy required to remove an electron from the valence band, and add it to vacuum. It is clear that this definition requires an absolute alignment of energy levels (with respect to vacuum, which defines the zero energy level). Furthermore, an electron has to leave the material through some surface to reach vacuum. Phenomenologically, it is well known that the ionization potentials as measured by experiments can depend strongly on the specific form and orientation of the surface.[15] Thus, first principles calculations of ionization

potentials require specific surface models.[16,17] This is in stark contrast to bulk properties such as band gaps. Here, we use surface potential lineups from Grüneis *et al.*[18] that were obtained with the HSE functional (geometries were obtained using PBE and then scaled to experimental lattice constants). Ionization potentials for other functionals are then obtained by adding bulk valence band shifts to the HSE ionization potentials listed in Supplementary Table 6. The bulk valence bands are aligned via average electrostatic potentials. For instance, for the DD-r^{2}SCANH functional, we obtain

$$\mathrm{IP}^{\text{DD-r}^2\text{SCANH}} = \mathrm{IP}^{\text{HSE}} + \left(\mathrm{VBM}^{\text{HSE}} - \mathrm{VBM}^{\text{DD-r}^2\text{SCANH}}\right). \quad (5)$$

The same procedure was used by Grüneis *et al.*[18] to calculate ionization potentials from many-body perturbation theory (listed as GWΓ in Supplementary Table 6). Instead of using the HSE surface lineups from Ref. 18 in Eq. (5), we have also tested PBE lineups from the same study. This changes the ionization potentials by 0.08 eV on average, which is on the order of experimental errors (see Supplementary Table 6). Larger discrepancies occur for ZnO and MgO where the differences amount to 0.19 eV and 0.32 eV, respectively. This is in agreement with Refs. 19 and 20, who discuss effects of charge reconstruction and surface dipoles for these materials. Furthermore, we find a difference of 0.48 eV for CdO, where PBE yields a false metallic ground state. The HSE lineups used here should be considered more accurate than the PBE lineups. Following Refs. 16 and 19, we have also excluded the ionization potentials for SiC from the statistical error analysis as the experimental data refer to a polar surface, see Supplementary Table 6.

SUPPLEMENTARY NOTE 3 - SPIN-ORBIT COUPLING

Spin-orbit coupling corrections[21] to the band gaps are explicitly calculated using the r^{2}SCAN functional,

$$\Delta E_{\mathrm{g}}^{\mathrm{SOC}} = E_{\mathrm{g}}^{\mathrm{r^2SCAN+SOC}} - E_{\mathrm{g}}^{\mathrm{r^2SCAN}}. \quad (6)$$

Results for $\Delta E_{\mathrm{g}}^{\mathrm{SOC}}$ are listed in Supplementary Table 2. To study the influence of the exchange-correlation functional on SOC, we have also calculated $\Delta E_{\mathrm{g}}^{\mathrm{SOC}}$ using PBE. We find that both functionals yield virtually identical SOC corrections for standard semiconductors and insulators (not shown), confirming the assessment of Huhn and Blum.[22] However, these authors also reported that PBE can be inaccurate for narrow-gap semiconductors that show large SOC corrections, see also Ref. 23. Here, we find that our r^{2}SCAN SOC corrections (-0.10 for Ge, -0.11 eV for InAs, -0.22 eV for GaSb, -0.24 eV for InSb) are in good agreement with corrections $\Delta E_{\mathrm{g}}^{\mathrm{SOC}}$ obtained from HSE calculations (-0.10 eV for Ge, -0.13 eV for InAs, -0.25 for GaSb, -0.26 eV for InSb). Among the materials studied here, the lead chalcogenides PbS, PbSe and PbTe exhibit the largest SOC corrections. Since $\Delta E_{\mathrm{g}}^{\mathrm{SOC}}$ even exceeds the actual experimental band gaps (see Supplementary Table 2), particular care has to be given to these materials. Supplementary Table 4 shows that our PBE results for $|\Delta E_{\mathrm{g}}^{\mathrm{SOC}}|$ slightly underestimate all-electron calculations (FP-APW+LO+rel. LO) from the literature [see Ref. 24, our projector augmented-wave (PAW) PBE results are identical to theirs]. These all-electron calculations should be considered as slightly more accurate, note also that VASP neglects certain SOC contributions outside the PAW spheres.[21] It is also evident that PBE somewhat underestimates $\Delta E_{\mathrm{g}}^{\mathrm{SOC}}$ for lead chalcogenides, whereas r^{2}SCAN SOC corrections are in good agreement with respective HSE results. We add the small differences between the all-electron PBE and PAW PBE results to our r^{2}SCAN values to obtain our best estimate of SOC in lead chalcogenides, see Supplementary Table 4.

Furthermore, SOC corrections are applied to the calculated valence band shifts, see Supplementary Table 6. We confirm the assessment of Ref. 18 that SOC corrections for band gaps exclusively stem from an upwards shift of the valence band. This means that $\Delta \mathrm{IP}^{\mathrm{SOC}} = \Delta E_{\mathrm{g}}^{\mathrm{SOC}}$ to very high accuracy (≤ 0.01 eV) for all materials listed in Supplementary Table 6. Heuristically, this can be understood from the fact that the valence bands are p-like and that the conduction bands are s-like (there is no SOC for s electrons).

We also calculate SOC corrections to the inverse dielectric constants using r^{2}SCAN,

$$\Delta \varepsilon_{\infty}^{-1,\mathrm{SOC}} = \varepsilon_{\infty}^{-1,\mathrm{r^2SCAN+SOC}} - \varepsilon_{\infty}^{-1,\mathrm{r^2SCAN}}. \quad (7)$$

Unsuprisingly, such SOC corrections to $\varepsilon_{\infty}^{-1}$ are negative and most significant for those materials that also show large negative SOC corrections to the band gaps. In particular, the values of $\Delta \varepsilon_{\infty}^{-1,\mathrm{SOC}}$ for lead chalcogenides (-0.021 for PbS, -0.024 for PbSe, and -0.016 for PbTe) are on the order of the experimental values themselves (0.054 for PbS, 0.040 for PbSe, and 0.027 for PbTe[25]). The addition of the SOC correction significantly improves the r^{2}SCAN inverse dielectric constant for these materials, see also Supplementary Table 5. However, the large SOC correction for the dielectric constant of InSb (-0.066, compared with an experimental value of 0.064[26]) is likely overestimated by r^{2}SCAN, as SOC erroneously closes the r^{2}SCAN band gap for InSb. In Supplementary Note 8, we discuss that self-consistent DD-r^{2}SCANH yields smaller correction of -0.010, similar to the HSE value of -0.007. We also include the SOC corrections to $\varepsilon_{\infty}^{-1}$ when calculating band gaps with non-empirical DDHs (see Table IV in the main text). In total, the SOC contributions are treated as follows

$$E_{\mathrm{g}}^{\mathrm{DD-DFTH+SOC}} = E_{\mathrm{g}}^{\mathrm{DDH}}(\alpha = \varepsilon_{\infty}^{-1,\mathrm{DFT}} + \Delta \varepsilon_{\infty}^{-1,\mathrm{SOC}}) + \Delta E_{\mathrm{g}}^{\mathrm{SOC}}. \quad (8)$$

Similarly, it has been pointed out by Cunningham *et al.*[27] that self-consistent GW calculations cannot neglect SOC corrections to the screened interaction W. To conclude, we further note that the SOC corrections $\Delta E_{\mathrm{g}}^{\mathrm{SOC}}$ and $\Delta\varepsilon_{\infty}^{-1,\mathrm{SOC}}$ converge faster with respect to the Brillouin zone sampling than the band gaps and inverse dielectric constants themselves. Thus, we can evaluate Eqs. (6) and (7) on smaller **k**-point meshes, reducing computational cost. Here, we find that an $10 \times 10 \times 10$ Γ-centered **k**-point mesh is sufficient to converge $\Delta E_{\mathrm{g}}^{\mathrm{SOC}}$ to an accuracy of 0.01 eV as well as to converge $\Delta\varepsilon_{\infty}^{-1,\mathrm{SOC}}$ to an accuracy of 0.001.
Finally, our calculated effective masses fully include SOC for each functional, see Supplementary Table 7. SOC corrections to equilibrium lattice constants are negligible even for materials with strong SOC effects such as InAs and InSb.[28] We have confirmed this explicitly by performing r^{2}SCAN calculations, finding that SOC corrections change lattice constants by less than 0.1% for every material studied.

SUPPLEMENTARY NOTE 4 - ZERO-POINT RENORMALIZATION

The calculated band gaps are corrected for zero-point renormalization (ZPR). Values for $\Delta E_{\mathrm{g}}^{\mathrm{ZPR}}$ are mostly taken from the literature,[27,29,30] see Supplementary Table 2. For CdO, Ar and Ne, $\Delta E_{\mathrm{g}}^{\mathrm{ZPR}}$ is calculated here using the special displacement method.[31,32] The calculations are done using r^{2}SCAN and use 128 atom supercell (CdO) or 64 atom supercells (Ar, Ne). This yields ZPR band gap corrections of -0.08 eV for CdO, -0.02 eV for Ar, and -0.08 eV for Ne. ZPR corrections for the valence band shift are mostly taken from Ref. 29. For a few materials not included in Ref. 29, we assume that the ZPR correction to the band gap originates in equal parts from a ZPR induced upshift of the valence bands and a ZPR induced downshift of the conduction bands (thus $\Delta\mathrm{IP}^{\mathrm{ZPR}} \approx \Delta E_{\mathrm{g}}^{\mathrm{ZPR}}/2$). This is a reasonable approximation given that the ZPR corrections are very small for these materials ($\Delta E_{\mathrm{g}}^{\mathrm{ZPR}} \leq 0.03$ eV). For the same reason, we completely neglect ZPR corrections to calculated effective masses. ZPR is also neglected in the calculation of dielectric constants. For selected materials, we have confirmed that the ZPR corrections to $\varepsilon_{\infty}^{-1}$ obtained by the special displacement method do not exceed typical experimental errors for $\varepsilon_{\infty}^{-1}$ (not shown). For the materials studied here, band gap ZPR corrections are largest for alkali halides, such as LiCl. However, the Clausius-Mossotti picture works well for these polar rock salt crystals, implying that the dielectric constant depends only on the density of ions, but not on their atomistic configurations.

SUPPLEMENTARY NOTE 5 - DIELECTRIC-DEPENDENT HYBRID FUNCTIONAL BENCHMARK

In this section, we present a detailed discussion of the band gap benchmark presented in Fig. 1 (b) in the main text. We construct different dielectric-dependent hybrid functionals via [copy of Eq. (9) in the main text]

$$E_{\mathrm{xc}}^{\mathrm{DD-DFTH}} = \varepsilon_{\infty}^{-1} E_{\mathrm{x}} + (1 - \varepsilon_{\infty}^{-1}) E_{\mathrm{x}}^{\mathrm{DFT}} + E_{\mathrm{c}}^{\mathrm{DFT}}. \tag{9}$$

For a fair comparison, we use the experimental dielectric constants listed in Supplementary Table 5 throughout. We consider six different DFT base functionals that are briefly summarized in the following.
The first rung of Jacob's Ladder is given by the local density approximation (LDA), here we use the parametrization of Perdew and Wang[33] (PW92). Historically, LDA was the first DFT functional, proposed already in the original work of Kohn and Sham.[34] The second rung of Jacob's Ladder is the generalized gradient approximation (GGA). We consider the popular Perdew-Burke-Ernzerhof functional (PBE) GGA functional. PBE is constructed via the method of exact constraint satisfaction[35] and yields well balanced results for various electronic structure properties. Meta-GGAs make up the third rung of Jacob's Ladder.[35,36] The Tao-Perdew-Staroverov-Scuseria (TPSS) functional is the first non-empirical meta-GGA functional from Perdew and coworkers.[37] TPSS generally yields modest, but consistent improvements over PBE.[35,37] TPSS was later superseded by the SCAN functional (strongly constrained and appropriately normed).[38] Here, we consider its regularized-restored variant r^{2}SCAN.[39] r^{2}SCAN is superior to SCAN in terms of numerical stability while retaining SCAN's overall accuracy. We also consider the r^{2}SCAN-L meta-GGA functional, which is a deorbitalized version of r^{2}SCAN. That is, r^{2}SCAN-L uses the Laplacian of the density $\nabla^2 n$ instead of the kinetic energy density τ as ingredient for the meta-GGA functional, which can be beneficial in terms of computational cost. Finally, we study the Lebeda-Aschebrock-Kümmel (LAK) meta-GGA functional.[40] LAK was recently proposed as a universal purpose meta-GGA functional that aims at improving upon SCAN and r^{2}SCAN in terms of band gaps, while keeping numerical stability and the overall accuracy of SCAN and r^{2}SCAN for other electronic structure properties.
Before discussing the band gaps obtained with the various DD-DFTH functionals, we analyze the performance of the DFT base functionals (see Supplementary Tables 2 and 3). In line with previous results,[23,41,42] we find that LDA yields the smallest band gaps, substantially underestimating the experimental ones (MARE 67%). Small, but consistent improvements are obtained by PBE (MARE 63%) and TPSS (MARE 60%). Further consistent improvements are achieved with r^{2}SCAN, which still underestimates the experimental band gaps (MARE 46%). Next, the band gaps of r^{2}SCAN-L are generally smaller than the band gaps of r^{2}SCAN (MARE 52%). This difference can be attributed to the fact that r^{2}SCAN-L misses the derivative discontinuity associated with the dependence

on τ.[41,43–45] Finally, the LAK gaps are consistently larger than the r^{2}SCAN ones. While we confirm the good performance of LAK for the band gaps for conventional semiconductors with E_g^{expt} between 1 eV and 4 eV,[40] we also find a clear systematic error that was not apparent in Ref. 40. Namely, the LAK band gap of narrow-gap semiconductors with $E_g^{expt} \lesssim 1$ eV are systematically overestimated, while the LAK band gaps of insulators with $E_g^{expt} \gtrsim 4$ eV are systematically underestimated. This behavior resembles that of a hybrid functional with fixed amount of Fock exchange (such as HSE or PBE0, see Supplementary Fig. 1). On average, LAK achieves a MARE of 33%, which is still better than r^{2}SCAN (MARE 46%), but not as good as HSE (MARE 16%). Focusing on the challenging narrow-gap semiconductors (see Table I in the main text), LDA and PBE yield qualitatively wrong states for all nine materials, TPSS for eight materials, and r^{2}SCAN-L for five materials. r^{2}SCAN yields only a single false metallic ground state (InSb), while LAK correctly predicts all nine materials to be semiconducting.

Next, we comment on the band gaps obtained with the dielectric-dependent hybrid functionals. Throughout, the admixing of Fock exchange increases the band gaps. These larger DD-DFTH band gaps systematically improve the agreement with experiment for all DFT base functionals but LAK. The band gaps of DD-LDAH (MARE 31%), DD-PBEH (MARE 27%) and DD-TPSSH (MARE 23%) remain systematically underestimated. The best performance is achieved by DD-r^{2}SCAN-LH (MARE 10%) and DD-r^{2}SCANH functional (MARE 7%), with no clear systematic error with respect to experiment. Since LAK already achieves good band gaps for conventional semiconductors, the increased band gaps of DD-LAKH now overshoot the experimental ones. The performance is even worse for narrow-gap semiconductors, where the already overestimated LAK band gaps are further deteriorated by DD-LAKH. However, DD-LAKH still improves upon LAK for insulators ($E_g^{expt} \gtrsim 4$ eV) and achieves an overall MARE of 26%, comparable to DD-PBEH (MARE 27%) and only slightly better than the LAK base functional itself (MARE 33%). The example of LAK highlights the difference between the two Jacob's Ladders shown in Fig. 1 (a) in the main text. LAK aims at approximating the full self-energy, (left Jacob's Ladder) but underperforms as a DDH functional (right Jacob's Ladder). For the purpose of DDH, a good DFT base functional does not necessarily have to yield very accurate band gaps on its own, but only needs to approximate the Coulomb hole well [the DFT remainder, see Eq. (12) in the main text]. The good performance of DD-r^{2}SCAN-LH further emphasizes this point. Namely, the r^{2}SCAN-L base functional is a pure Kohn-Sham DFT functional, which suggests that the Coulomb hole can be well approximated even by a fully local exchange-correlation potential. Still, currently we recommend to use r^{2}SCAN over r^{2}SCAN-L due to the better performance of DD-r^{2}SCANH functional. The advantages of r^{2}SCAN-L in terms of computational efficiency are not relevant for the DDH functionals, as the evaluation of the Fock exchange part dominates the overall cost of the DDH calculation.

SUPPLEMENTARY NOTE 6 - ANALYSIS OF THE HYBRID FUNCTIONAL BAND GAP SLOPES

In the following, we will further investigate the interesting correlation observed between the hybrid functional band gap slopes and the inverse dielectric constants (see Fig. 6 in the main text). For the sake of convenience, we start by repeating the central equations. A global hybrid functional is given by [copy of Eq. (5) in the main text]

$$E_{xc}^{DFTH}(\alpha) = \alpha E_x + (1-\alpha)E_x^{DFT} + E_c^{DFT}. \tag{10}$$

Dielectric-dependent hybrid functionals (DDH) are obtained by specifying $\alpha = \varepsilon_\infty^{-1}$. Assuming that the electronic orbitals of the hybrid functional do not vary significantly with respect to those of its DFT base functional, the band gap increases approximately linearly with the mixing parameter α [copy of Eq. (14) in the main text],

$$E_g^{DFTH}(\alpha) \simeq E_g^{DFT} + \alpha(E_g^{HF+DFT_c} - E_g^{DFT}). \tag{11}$$

Within this approximation, the hybrid functional band gap slopes are given by [copy of Eq. (15) in the main text]

$$\frac{dE_g^{DFTH}}{d\alpha}(\alpha) \simeq E_g^{HF+DFT_c} - E_g^{DFT}. \tag{12}$$

Analogously, the valence band edges exhibit a linear down-shift as a function of α,[46–48]

$$-\mathrm{VBM}^{DFTH}(\alpha) \simeq -\mathrm{VBM}^{DFT} + \alpha(\mathrm{VBM}^{HF+DFT_c} - \mathrm{VBM}^{DFT}), \tag{13}$$

and the corresponding VBM slopes are given by

$$-\frac{d\mathrm{VBM}^{DFTH}}{d\alpha}(\alpha) \simeq \mathrm{VBM}^{HF+DFT_c} - \mathrm{VBM}^{DFT}. \tag{14}$$

Supplementary Figure 3 (a) shows these VBM slopes for the PBEH and r^{2}SCANH functionals. Similar to the band gap shifts (see Fig. 6 in the main text), the negative VBM slopes are smaller for r^{2}SCANH, indicating that the r^{2}SCAN base functional better approximates Fock exchange than PBE. Extending the discussion in the main text, we further conclude that the improved IPs obtained with DD-r^{2}SCANH profit from a better treatment of the DFT

remainder by the r^2SCAN base functional as well [see Fig. 4 (b) in the main text]. Moreover, Supplementary Fig. 3 (a) shows that the linear trend found for the band gap slopes of semiconductors ($\varepsilon_\infty^{-1,\mathrm{expt}} \lesssim 0.25$, see Fig. 6 in the main text) is also observed for the valence band shifts. Supplementary Figure 3 (b) shows the relative valence band shifts given by[46,48]

$$\delta^{\mathrm{VBM}} = -\frac{\mathrm{dVBM}^{\mathrm{DFTH}}/\mathrm{d}\alpha}{\mathrm{d}E_\mathrm{g}^{\mathrm{DFTH}}/\mathrm{d}\alpha}. \quad (15)$$

If this ratio is equal to 50%, the valence and conduction band edges open up symmetrically when Fock exchange is added. Chen and Pasquarello[48] have previously found that δ^{VBM} for PBEH is typically 50% - 60% for typical semiconductors, but larger for polar materials. We confirm this result here, and further establish that δ^{VBM} are slightly larger for r^2SCANH than for PBEH. That is, Fock exchange shifts the band edges of r^2SCAN more asymmetrically than the PBE band edges.

So far, we have established that linear trends occur for the band gaps slopes and the VBM slopes of both PBEH and r^2SCANH. However, there is also same scatter, and the trends seem to break down for insulators ($\varepsilon_\infty^{-1,\mathrm{expt}} \gtrsim 0.25$). Furthermore, the physical origin of such linear relations is not entirely clear yet. Inspired by the work of Fiorentini and Baldereschi,[49] we now relate the non-empirical band gap corrections,

$$\Delta E_\mathrm{g} = E_\mathrm{g}^{\mathrm{DD-DFTH}}(\alpha = \varepsilon_\infty^{-1,\mathrm{DFT}}) - E_\mathrm{g}^{\mathrm{DFT}}, \quad (16)$$

to $\varepsilon_\infty^{-1,\mathrm{DFT}}$, the inverse dielectric constant calculated using the DFT base functionals. Supplementary Figure 4 shows this relation for both DD-PBEH and DD-r^2SCANH. Originally, Fiorentini and Baldereschi[49] (FB) proposed a linear relation,

$$\Delta E_\mathrm{g}^{\mathrm{FB}} = A\varepsilon_\infty^{-1}. \quad (17)$$

Here, we find that a quadratic relation better describes our data (see Supplementary Fig. 4),

$$\Delta E_\mathrm{g}^{\mathrm{FBM}} = A\varepsilon_\infty^{-1,\mathrm{DFT}} + B\varepsilon_\infty^{-2,\mathrm{DFT}}. \quad (18)$$

The extra quadratic term is physically motivated by the Moss relation.[50,51] We have introduced the shortcut FBM after the authors of Refs. 49 and 50. The quadratic form for ΔE_g is consistent with the linear relation shown in Fig. 6 in the main text. That is, ΔE_g can be obtained by multiplying the band gap slopes in Eq. (12) with ε_∞^{-1}. Supplementary Figure 4 shows that the quadratic fits work exceedingly well for semiconductors. The scatter is largely eliminated, however, we also see that the deviations for insulators remain. To understand this, we note that the linear form [Eq. (17)] can be motivated directly from Eq. (11),

$$\begin{aligned}\Delta E_\mathrm{g} &\simeq \varepsilon_\infty^{-1}(E_\mathrm{g}^{\mathrm{HF+DFT_c}} - E_\mathrm{g}^{\mathrm{DFT}}) = \varepsilon_\infty^{-1}(\frac{\mathrm{dCBM}}{\mathrm{d}\alpha} - \frac{\mathrm{dVBM}}{\mathrm{d}\alpha}) \\ &\simeq \varepsilon_\infty^{-1}(\langle\mathrm{CBM}|\,\Sigma_\mathrm{x} - v_\mathrm{x}^{\mathrm{DFT}}\,|\mathrm{CBM}\rangle - \langle\mathrm{VBM}|\,\Sigma_\mathrm{x} - v_\mathrm{x}^{\mathrm{DFT}}\,|\mathrm{VBM}\rangle),\end{aligned} \quad (19)$$

where the hybrid functional slopes of the valence and conduction bands are defined via Eq. (14). Crudely approximating the term in brackets in the last line as a material independent constant A yields Eq. (17). More realistically, A can be weakly varying within a set of chemically similar materials such as octet semiconductors. The derivation of Eq. (17) given by Fiorentini and Baldereschi[49] makes similar assumptions. To conclude, we note that both the linear relation [Eq. (17)] as well as the quadratic relation [Eq. (18)] correctly capture the fact the ΔE_g should vanish for a (real) metal.

Finally, we note that the original intention of Fiorentini and Baldereschi[49] and Moss[50] was to use empirical relations such as Eq. (18) in a predictive way. That is, we can estimate the DD-DFTH band gap simply by performing a DFT ground state calculation. Our fit for DD-PBEH is

$$E_\mathrm{g}^{\mathrm{DD-PBEH}}(\mathrm{eV}) = E_\mathrm{g}^{\mathrm{PBE}}(\mathrm{eV}) + 5.542\varepsilon_\infty^{-1,\mathrm{PBE}} + 11.017\varepsilon_\infty^{-2,\mathrm{PBE}}, \quad (20)$$

and exhibits an excellent MAE of 0.18 eV (measured against the first principles data). This should be compared with the accuracy of DD-PBEH (MAE 0.50 eV measured against experimental data, see Table IV in the main text). Our fit for DD-r^2SCANH reads

$$E_\mathrm{g}^{\mathrm{DD-r^2SCANH}}(\mathrm{eV}) = E_\mathrm{g}^{\mathrm{r^2SCAN}}(\mathrm{eV}) + 5.023\varepsilon_\infty^{-1,\mathrm{r^2SCAN}} + 8.958\varepsilon_\infty^{-2,\mathrm{r^2SCAN}}, \quad (21)$$

reaching an MAE of 0.22 eV (measured against the first principles data), which is similar to the accuracy of DD-r^2SCANH (MAE 0.18 eV measured against experimental data, see Table IV in the main text). These empirical relations could be useful as a pre-screening step in high-throughput studies such as Ref. 52. Interestingly, this study also found that the dielectric constant is very useful for the prediction of band gaps.

SUPPLEMENTARY NOTE 7 - STRONGLY CORRELATED TRANSITION METAL OXIDES

Our work mainly focuses on conventional semiconductors and insulators that are experimentally well characterized. Still, it is interesting to apply DD-r^{2}SCANH to more challenging strongly-correlated materials. Here, we consider the rock salt antiferromagnetic monoxides MnO, FeO, CoO, and NiO as prototypical examples for strong correlation. These materials have recently been used as a testbed for dielectric-dependent hybrid functionals[53–56] and many-body perturbation theory.[27,57,58] Following these previous studies, we model the four antiferromagnetic monoxides in the ideal rock salt structure, neglecting small atomic displacements. As we shall see, a precise quantitative analysis is precluded by the fact that both experimental data and benchmark first-principles calculations show large spreads of results. In particular, this is the case for FeO, which is also the most challenging material due to its particularly strong correlation effects. As such, the discussion of these materials is necessarily more qualitative and speculative.
We begin by calculating dielectric constants using the PBE and r^{2}SCAN functionals, see Supplementary Table 9. PBE yields a false metallic ground state for FeO and CoO, and strongly underestimates the inverse dielectric constants of NiO and MnO. In contrast, r^{2}SCAN correctly predicts a semiconducting ground state for all four transition metal monoxides, see also Refs. 59 and 60. We note that it is necessary to allow for symmetry breaking of the electronic orbitals to obtain the correct ground state for CoO and FeO.[58,59] Furthermore, the r^{2}SCAN inverse dielectric constants significantly improve upon the PBE ones. For MnO, the agreement with experiment is very satisfactory, while the inverse dielectric constants for FeO, CoO, and NiO also improve but still remain underestimated. The close numerical agreement of the PBE dielectric constants of NiO and MnO with PBE references obtained with density functional perturbation theory (DFPT) demonstrates that the finite field method (PEAD) remains very accurate for spin-polarized systems, see also Supplementary Note 1. We note that the r^{2}SCAN inverse dielectric constants for NiO (0.139) is in good agreement with a recent calculation from Wang et al.,[60] who reported 0.133 for NiO. However, our r^{2}SCAN inverse dielectric constants for CoO (0.137) is significantly smaller than the value of 0.182 given in Ref. 60. It is also interesting to compare the performance of r^{2}SCAN with state-of-the-art many-body perturbation theory techniques (see MBPT in Supplementary Table 9). Some spread in the MBPT results is due to different starting points used for the Bethe-Salpeter equation. In particular, the MBPT calculations in Refs. 27 and 58 can be viewed as GWΓ with approximate vertex corrections. However, large differences also occur purely due to code implementation and numerical convergence, especially for FeO.[58] Overall, r^{2}SCAN is surprisingly competitive with MBPT considering the enormous effort of the MBPT calculations. Our results suggest that r^{2}SCAN can predict accurate dielectric constants even when strong correlation is present.
Next, we calculate DD-PBEH and DD-r^{2}SCANH band gaps using the experimental inverse dielectric constants (averages are taken if multiple experimental data are available), see Supplementary Table 10. Compared to experimental data and MBPT references, DD-PBEH systematically underestimates the band gaps, consistent with the performance of DD-PBEH for conventional semiconductors (see Supplementary Table 2). In particular, DD-PBEH underestimates the band gap of MnO by ≈ 1 eV. DD-r^{2}SCANH yields larger band gaps than DD-PBEH, which is also in line with the trend for conventional semiconductors. The larger DD-r^{2}SCANH band gap leads to an improvement for MnO, but the DD-r^{2}SCANH band gap for CoO and NiO is too large (the overestimation is roughly 0.5 eV for CoO and 1 eV for NiO). Concerning the band gap of FeO, we note that both DD-PBEH and DD-r^{2}SCANH fall within the very large spread of reference results. We note that the use of DFT dielectric constants would tilt the results strongly in favor of DD-r^{2}SCANH, since (i) band gaps of CoO and NiO would be reduced, and (ii) DD-PBEH would yield false metallic states for CoO and FeO. Moreover, the large overestimation for NiO might indicate a potential route of improvement for DD-r^{2}SCANH. Namely, Zhan, Govoni, and Galli[56] have shown that a range-separated DDH functional (DD-RSH-CAM) yields a band gap of 5.15 eV, close to our DD-r^{2}SCANH result (5.07 eV). They further showed that the band gap is reduced by 0.46 eV if local variations of the dielectric constant (and of the range-separation parameter) are considered.[61,62] Based on data given in Ref. 61, one can expect that applying this scheme to DD-r^{2}SCANH could also address the slight overestimation of insulator band gaps (for example Ne, see Supplementary Table 2). Furthermore, we note that DD-r^{2}SCANH similarly overestimates the band gap of the nonmagnetic rock salt monoxide CdO (see Supplementary Table 2). Finally, we note that HSE yields overall reasonable band gaps for the transition metal monoxides. However, Liu et al.[55] noted that HSE overestimates the inverse dielectric constants, see Supplementary Table 9. Concluding the band gap analysis, Supplementary Fig. 6 shows that the four strongly correlated transition metal monoxides are clear outliers in terms of the hybrid functional band-gap slopes. The clear violation of the relationship between band gap and inverse dielectric constant elucidates why these materials are challenging for dielectric-dependent hybrid functionals.[54,55]
Last, the magnetic moment of the transition metal monoxides are shown in Supplementary Table 11. Generally, the magnetic moments are roughly proportional to the band gap and the influence of the particular functional is strongly reduced. In agreement with previous studies,[59,60] we find that r^{2}SCAN yields larger magnetic moments than PBE. This improves the agreement with experimental values and MBPT references, and has been interpreted as better description of self-interaction.[59,60] While the r^{2}SCAN magnetic moments are still slightly too small, the DD-r^{2}SCANH magnetic moments are in excellent agreement with MBPT and experiment. However, it should be stated that accurate magnetic moments are also achieved by any decent hybrid functional, see also Ref. 55.
In conclusion, we find that r^{2}SCAN clearly improves upon PBE for electronic structure properties of the rock salt transition metal monoxides. However, DD-r^{2}SCANH is only superior to DD-PBEH if one uses the inverse dielectric

constants obtained with the respective DFT base functionals, rather than the experimental ones.

SUPPLEMENTARY NOTE 8 - SELF-CONSISTENCY IN THE DIELECTRIC CONSTANTS

Throughout this work, we have mainly used inverse dielectric constants from experiment as mixing parameter [$\alpha = \varepsilon_\infty^{-1,\mathrm{expt}}$, see Eq. (13) in the main text]. Table IV in the main text also explores inverse dielectric constants calculated with the respective DFT base functionals ($\alpha = \varepsilon_\infty^{-1,\mathrm{DFT}}$, see also Supplementary Figs. 1). There, we discuss also a second way of obtaining first principles dielectric constants, namely the self-consistent DDH scheme introduced by Skone, Govoni, and Galli.[6] That is, given an initial estimate for the mixing parameter $\alpha^{(0)}$, one calculates the inverse dielectric constant with the dielectric-dependent hybrid functional DDH($\alpha = \alpha^{(0)}$). The obtained inverse dielectric constant serves as next iteration for the mixing parameter ($\alpha^{(1)} = 1/\varepsilon_\infty^{\mathrm{DDH}(\alpha_0)}$), and this process is iterated until self-consistency is reached (for instance, some stopping criterion can be applied either for α or for the band gap). Even though this self-consistent scheme is theoretically satisfying, the computational cost associated with evaluating the dielectric constants several times at the hybrid functional level is large.

In the following, we evaluate the self-consistent DDH scheme for two selected materials (GaAs and InSb), comparing DD-r^{2}SCANH with DD-PBEH. To reduce computational cost, the dielectric constants are evaluated on a reduced **k**-point grid ($8 \times 8 \times 8$ Γ-centered mesh compared to a $16 \times 16 \times 16$ Γ-centered mesh). For GaAs, the difference obtained by comparing $\varepsilon_\infty^{-1,\mathrm{r^2SCAN}}$ for the two **k**-point meshes amounts to a small difference of -0.002, which is added as a correction to the DDH calculations (PBE also yields the same correction of -0.002). For InSb, the $8 \times 8 \times 8$ Γ-centered mesh is already sufficient to converge the inverse dielectric constants within our target accuracy of 0.001.

First, we study GaAs as a prototypical semiconductor. For reference, the hybrid functional band gaps as a function of α are shown in Fig. 2 (a) in the main text. We start the self-consistent iteration by using the inverse dielectric constants calculated with the DFT base functionals as initial mixing parameter ($\alpha^{(0)} = \varepsilon_\infty^{-1,\mathrm{DFT}}$). Supplementary Figure 8 (a) shows that convergence is very fast, and that the inverse dielectric constant increases slightly during self-consistency. This is the typical behavior found also in previous studies.[6,51,53] In particular, the inverse dielectric constant obtained with self-consistent DD-PBEH is 0.082, which is $\approx 11\%$ larger than the value of 0.074 obtained by the PBE base functional. Self-consistent DD-r^{2}SCANH yields a dielectric constant of 0.092, which is $\approx 6\%$ larger than the value of 0.087 obtained with the r^{2}SCAN base functional. We note that the inverse dielectric constant obtained with self-consistent DD-r^{2}SCANH is also in excellent agreement with the experimental value of 0.095 (see Supplementary Table 5). The increased inverse dielectric constants result from the larger hybrid functional gaps (compared to the DFT base functionals). Furthermore, the smaller self-consistency effect for DD-r^{2}SCAN can be understood as a consequence of the smaller hybrid functional band gap slopes [see Fig. 2 (a) in the main text].

Next, we treat the rather special case of InSb. InSb is the only material studied here, where r^{2}SCAN yields a false metallic ground state, and the analysis of this narrow-gap semiconductor is further complicated by the large spin-orbit coupling correction to ε_∞^{-1}. To add some independent reference, and to disentangle the treatment of spin-orbit coupling, we also perform HSE calculations. First, we note that r^{2}SCAN does not yield a spurious band inversion as PBE does (see Table I in the main text). This is also demonstrated in Supplementary Fig. 7, where we show that PBEH yields negative band gaps for a substantial range of mixing parameters ($\alpha \lesssim 0.11$). In contrast, r^{2}SCANH only needs tiny amounts of Fock exchange to open a band gap. To avoid the metallic phase, we initialize our self-consistent DDH calculations with $\alpha^{(0)} = 1/4$. For the sake of computational efficiency, spin-orbit coupling is only included as a post-processing correction (discussed later). Even without spin-orbit coupling, DD-PBEH eventually converges back to the metallic solution also found by PBE, confirming the result of Cui et al.[14] The self-consistent DD-r^{2}SCANH calculation yields an inverse dielectric constant of 0.074, which is close to the HSE value (0.076) and $\approx 12\%$ larger than the value obtained by the r^{2}SCAN base functional (0.066). The spin-orbit correction obtained by r^{2}SCAN is equal to -0.066, as the inclusion of spin-orbit coupling closes the band gap for r^{2}SCAN. As discussed in Supplementary Note 3, this value is likely overestimated. For self-consistent DD-r^{2}SCANH(α=0.074), we obtain a spin-orbit coupling correction of -0.010, in good agreement with the HSE value of -0.007. Applying this correction yields 0.064 for the inverse dielectric constant of self-consistent DD-r^{2}SCANH, in excellent agreement with the experimental value of 0.064 (see Supplementary Table 5).

Finally, we comment on the reason why self-consistent DD-PBEH converges to a false metallic ground state. As Supplementary Fig. 7 shows, the spurious PBE band inversion causes a large region of mixing parameters corresponding to a false metallic phase for PBEH. For any α inside this range, this directly implies $\varepsilon_\infty^{-1} = 0$. Thus the basin of attraction of the self-consistent DDH cycle has to include at least the range $\alpha \lesssim 0.11$, and, as our calculations show, it is in fact even larger. Similarly, Cui et al. also found that self-consistent DD-PBEH converges to a false metallic ground state for InAs, where PBE also has a substantial negative band gap [see Fig. 2 (a) in the main text]. In contrast, the lack of spurious band inversion for r^{2}SCAN means that the basis of attraction for the false metallic ground state can be smaller. However, one needs to choose a better starting point inside the basin of attraction of the semiconducting phase, since it is clear that at least $\alpha = 0$ represents a fixed point for the self-consistent DDH calculation in all cases where the DFT base functional yields a false metallic ground state. To ensure a unique semiconducting solution (independent of starting point), the DFT base functional also needs to yield the correct ground state. We also note that in rare cases the opposite problem can occur, namely standard hybrid functionals

yielding a false semiconducting ground state for a real metal.[63] This might also imply that self-consistent DDH could have some basin of attraction for the false semiconducting solution. However, in this opposite case the fixed point $\alpha = 0$ guarantees a correct metallic solution for self-consistent DDH provided that the DFT base functional also yields a metallic ground state.

In summation, we consider these selected results to be quite satisfactory. Namely, for GaAs, self-consistency for DD-r^{2}SCANH slightly increases ε_∞^{-1} bringing it closer to the experimental value. A closer inspection of Fig. 3 (b) in the main text and Supplementary Table 5 shows that the inverse dielectric constants of r^{2}SCAN slightly underestimate the experimental ones on average (MSRE -9%), leaving room for further improvement upon self-consistency. We have also shown that self-consistency brings qualitative improvements for the challenging case of InSb, where self-consistent DD-r^{2}SCANH can escape the false metallic phase while self-consistent DD-PBEH cannot. Thus, self-consistent DD-r^{2}SCANH has the potential to yield even more accurate dielectric constants than its r^{2}SCAN base functional. However, due to the large computational overhead, we recommend self-consistent DD-r^{2}SCANH only in special cases such as InSb. The use of r^{2}SCAN inverse dielectric constants should be sufficient for many applications including high-throughput studies.

SUPPLEMENTARY FIGURES

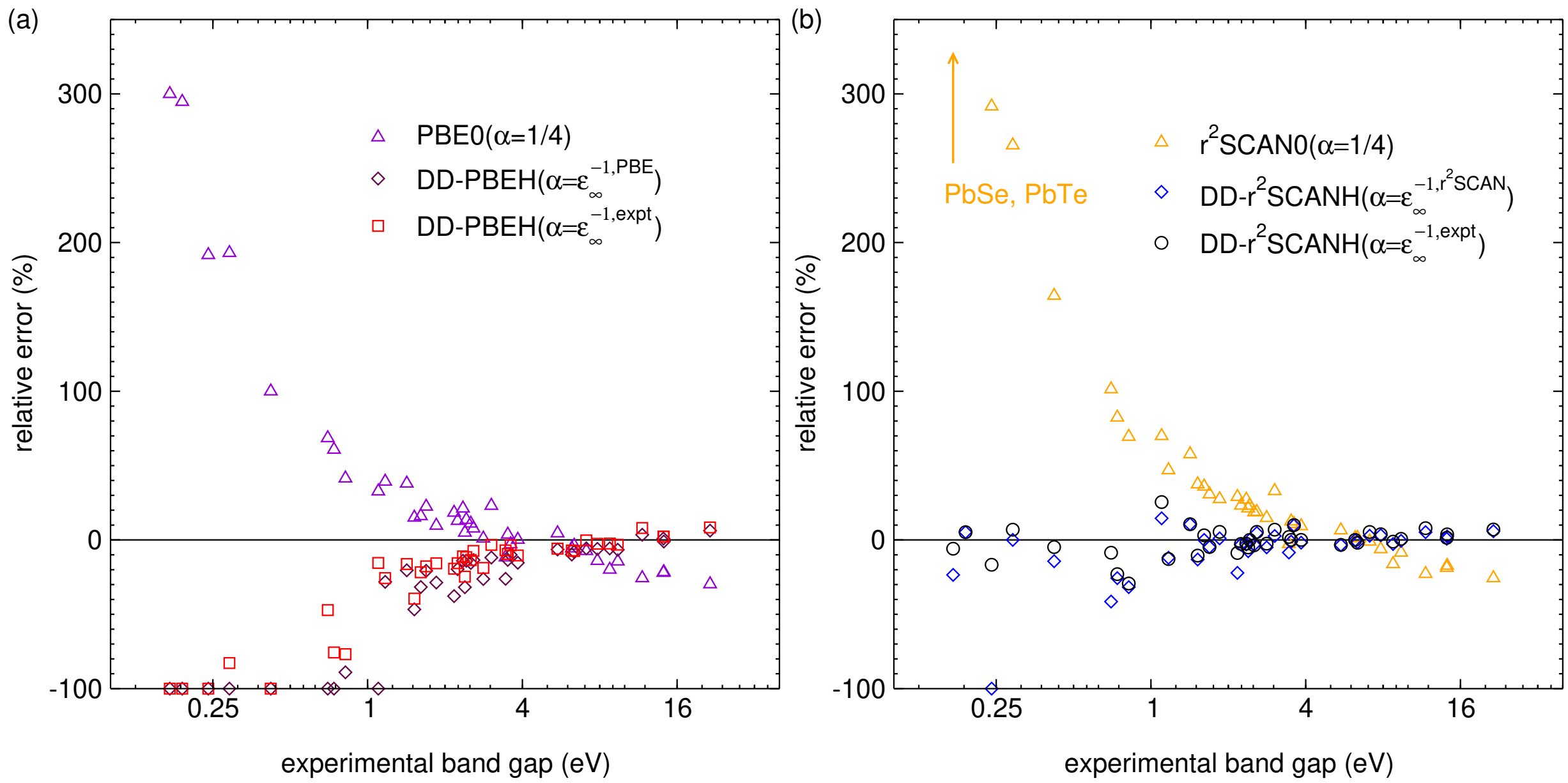

Supplementary Figure 1. **Non-empirical DD-r^{2}SCANH via calculating ε_∞^{-1} with r^{2}SCAN.** Band gaps for 39 semiconductors and insulators as in Fig. 4 (a) in the main text. Here, we compare different global hybrid functionals based on (a) PBE, and (b) r^{2}SCAN, respectively. It is evident that the use of a fixed mixing parameter $\alpha = 1/4$ (PBE0 and r^{2}SCAN0) leads to significantly overestimated band gaps for narrow-gap semiconductors, whereas insulator gaps are systematically underestimated. The use of a DFT mixing parameter ($\alpha = \varepsilon_\infty^{-1,\mathrm{DFT}}$) closely approximates the semi-empirical hybrid functional ($\alpha = \varepsilon_\infty^{-1,\mathrm{expt}}$), especially for DD-r^{2}SCANH. Statistics are given in Table IV in the main text. Note the different ordinate scale compared to Fig. 4 (a) in the main text. r^{2}SCAN0 overestimates the experimental band gaps of PbSe and PbTe by roughly 400%, these two data points are outside of the plotted error range. Source data are provided as a Source Data file.

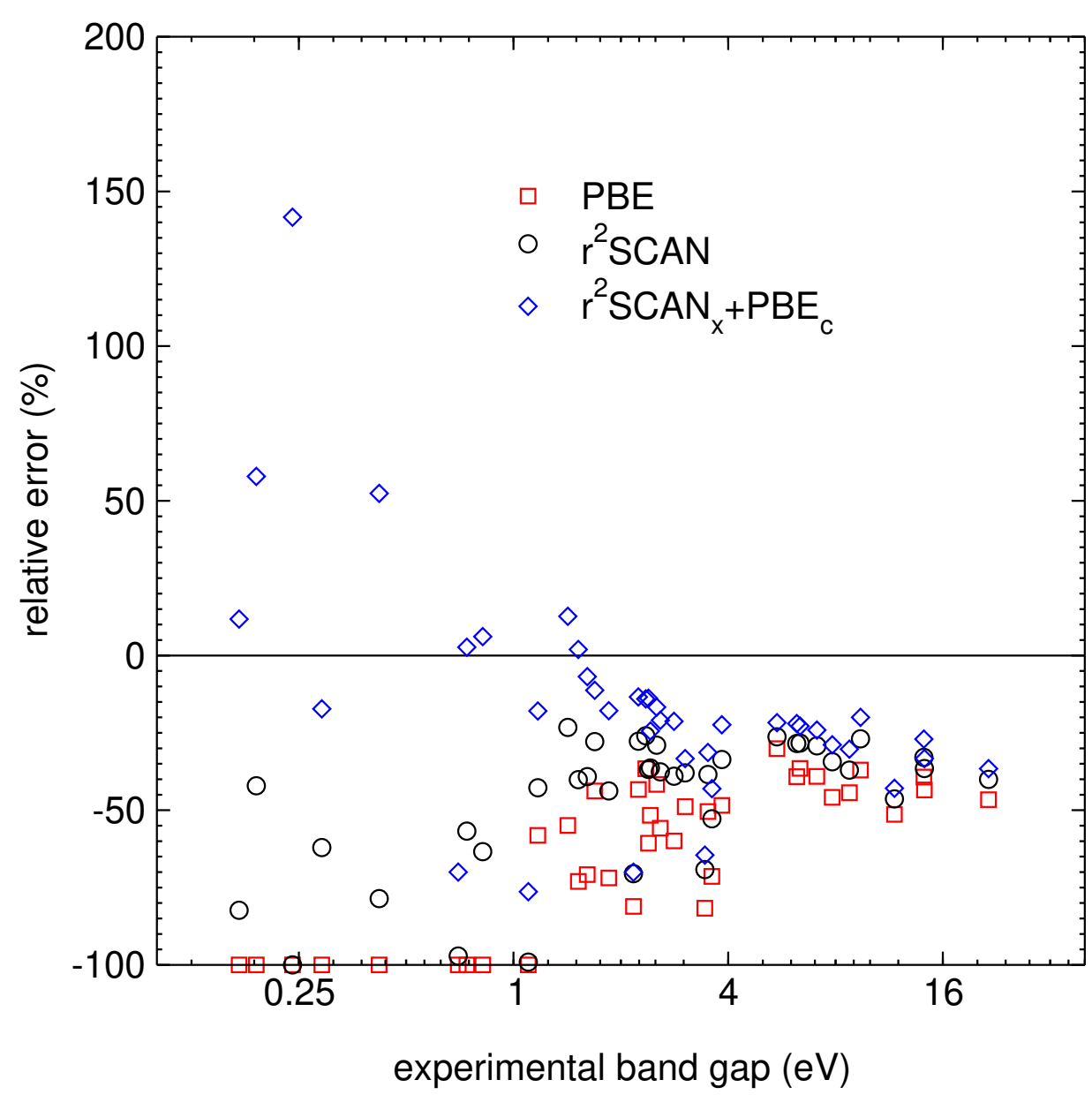

Supplementary Figure 2. **Role of r^{2}SCAN exchange and correlation.** Band gaps for 39 semiconductors and insulators as in Fig. 3 (a) in the main text. Here, we also show a DFT functional composed of r^{2}SCAN exchange and PBE correlation. On the one hand, the effect of replacing PBE exchange with r^{2}SCAN exchange is evident by comparing the mixed functional with PBE. The more nonlocal meta-GGA exchange drastically increases the band gaps for semiconductors in particular ($E_\mathrm{g}^\mathrm{expt} \lesssim 5$ eV). On the other hand, by comparing the mixed functional with r^{2}SCAN, one can see the effect of replacing PBE correlation with r^{2}SCAN correlation. The more nonlocal meta-GGA correlation partially cancels the effect of exchange. In total, the r^{2}SCAN gaps remain somewhat larger than the PBE gaps. Note the different ordinate scale compared to Fig. 3 (a) in the main text. Source data are provided as a Source Data file.

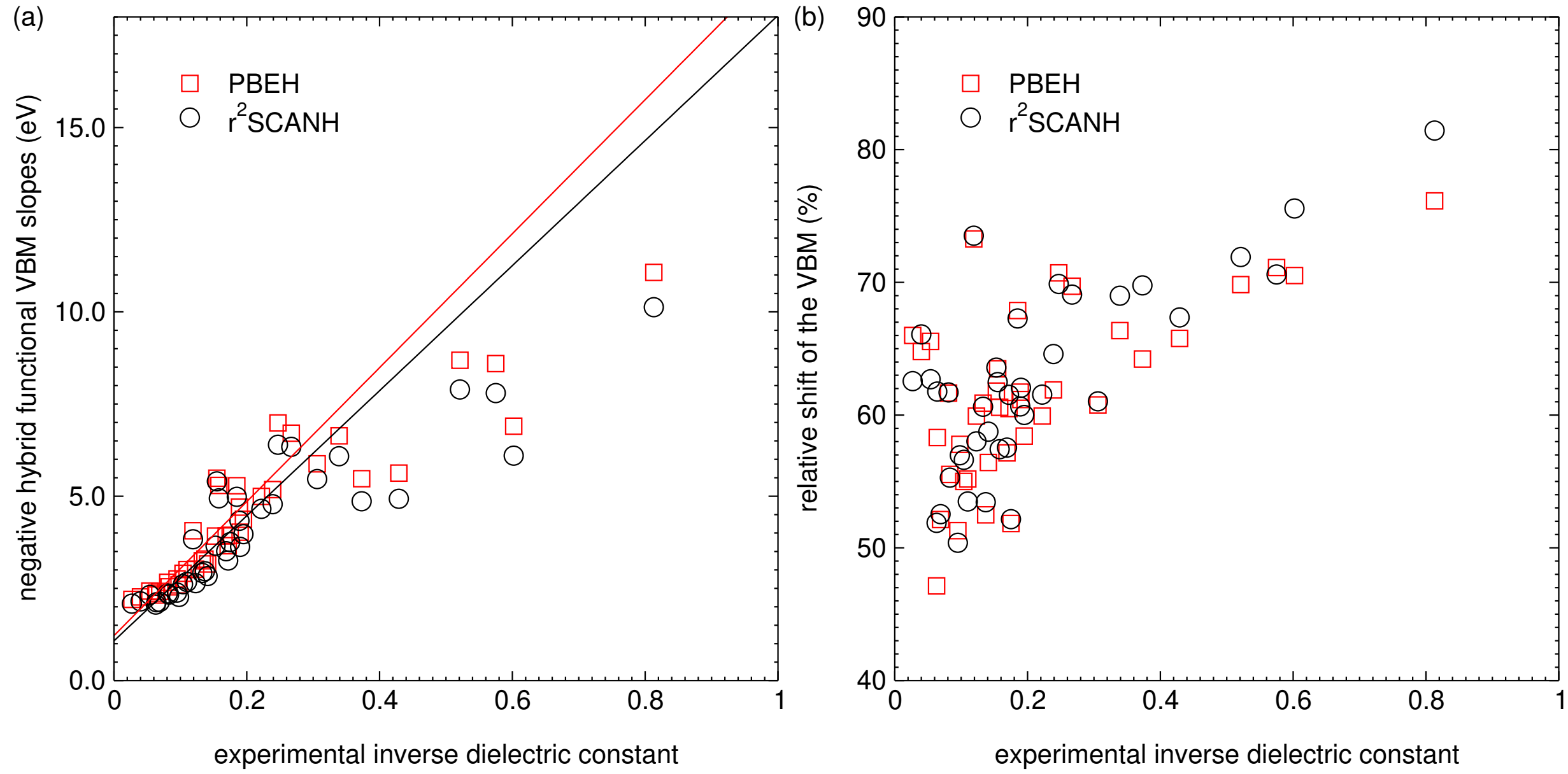

Supplementary Figure 3. **Analyzing the VBM shifts and their contribution to the DDH band gaps.** (a) Hybrid functional slopes as in Fig. 6 in the main text, but here for the negative valence band shifts, $-\mathrm{dVBM}^{\mathrm{DFTH}}/\mathrm{d}\alpha$ [Eq. (14)]. Specifically, the slopes are calculated as $-[\mathrm{VBM}^{\mathrm{DFTH}}(\alpha = \varepsilon_\infty^{-1,\mathrm{expt}}) - \mathrm{VBM}^{\mathrm{DFT}}]/\varepsilon_\infty^{-1,\mathrm{expt}}$. Smaller slopes for DD-r^{2}SCANH indicate a better description of Fock exchange by the r^{2}SCAN base functional, see Eq. (14). The valence band shifts are strongly correlated to $\varepsilon_\infty^{-1,\mathrm{expt}}$, mirroring the band gap trend shown in Fig. 6 in the main text. Straight lines present linear fits to semiconductor data ($\varepsilon_\infty^{-1,\mathrm{expt}} \leq 0.25$). (b) The relative shift of the VBM, δ^{VBM}, measures how symmetrically a global hybrid functional opens the band gap [see Eq. (15)]. In agreement with Chen and Pasquarello,[48] we find that δ^{VBM} is roughly 50%-60% for typical semiconductors, and larger for insulators. Overall, DD-r^{2}SCANH yields slightly larger values for δ^{VBM} than DD-PBEH. Source data are provided as a Source Data file.

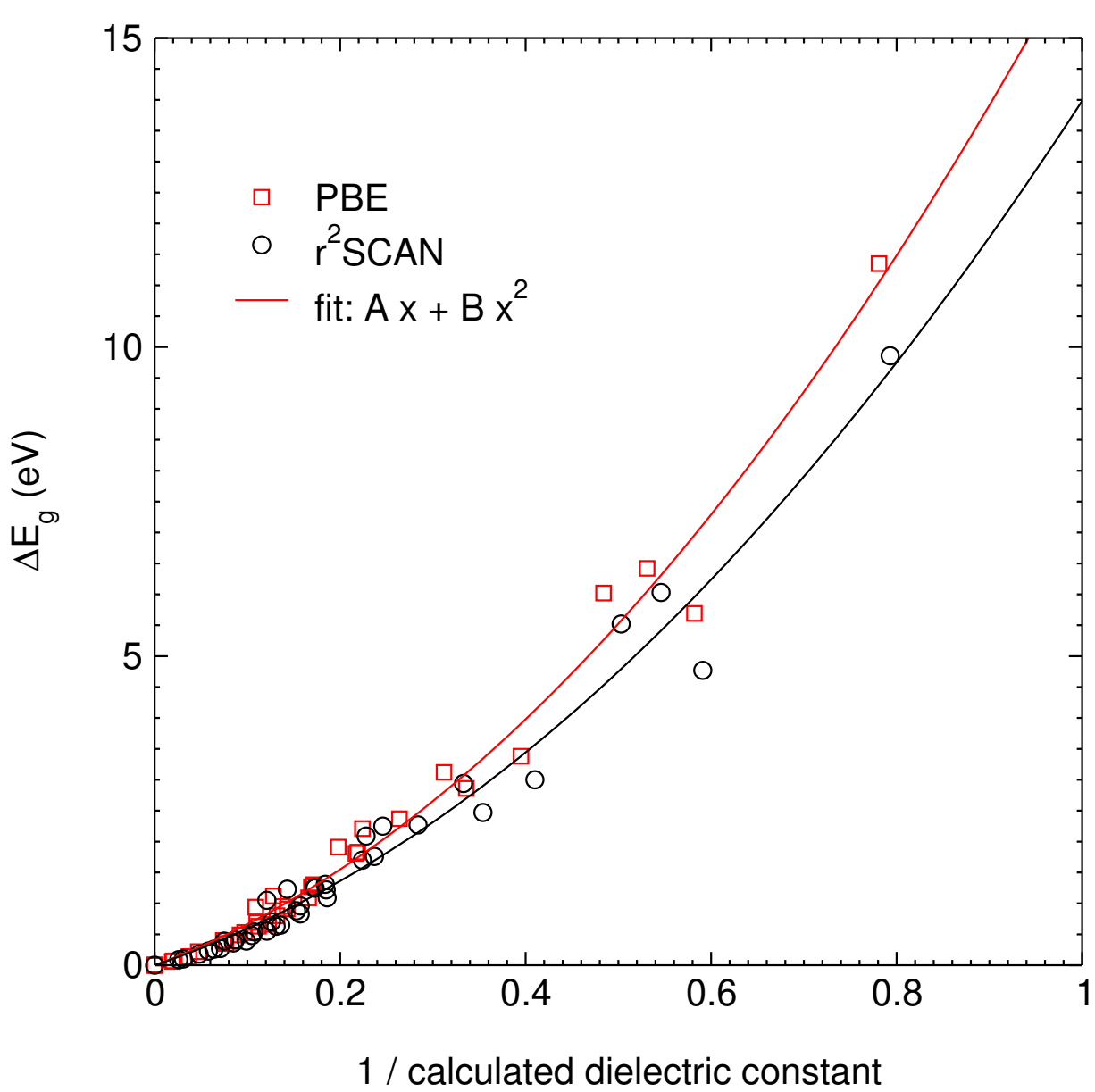

Supplementary Figure 4. **Simple model for the DDH band gaps.** Hybrid functional band gap correction ΔE_{g} [see Eq. (16)] as a function of the inverse dielectric constant $\varepsilon_\infty^{-1,\mathrm{DFT}}$. Quadratic fits are inspired by the works of Fiorentini, Baldereschi, and Moss,[49,50] see Eq. (18). Fit parameters for PBE and r^{2}SCAN are given in Eqs. (20) and (21), respectively. The good agreement of the empirical fits with the first principles data implies that a ground state calculation (yielding $E_{\mathrm{g}}^{\mathrm{DFT}}$ and $\varepsilon_\infty^{-1,\mathrm{DFT}}$) can be sufficient to predict hybrid functional band gaps within a set of electronically similar materials. This could be useful for high-throughput screening of band gaps. Source data are provided as a Source Data file.

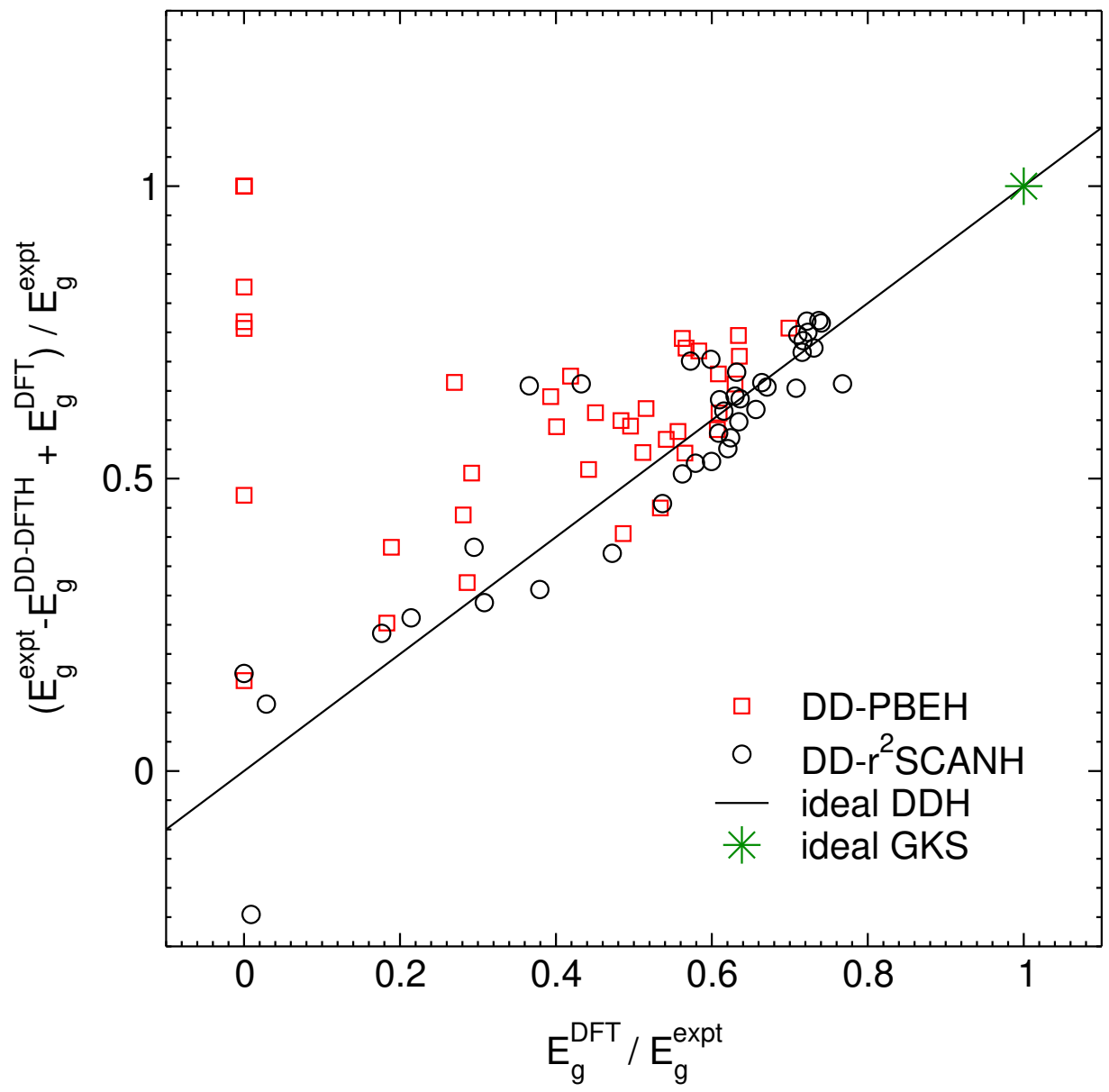

Supplementary Figure 5. **Summary of the band gap improvements for DD-r^{2}SCANH.** (i) The band gaps of the r^{2}SCAN base functional are closer to experiment than PBE, corresponding to right-shifted symbols for DD-r^{2}SCANH. (ii) The band gap slopes of r^{2}SCANH are smaller than the band gap slopes of PBEH, corresponding to up-shifted symbols for DD-r^{2}SCANH. (iii) The symbols for DD-r^{2}SCANH are closer to the 45° line that indicates an ideal DDH (this line indicates $E_{\mathrm{g}}^{\mathrm{DD-DFTH}} = E_{\mathrm{g}}^{\mathrm{expt}}$). Thus, the DFT remainder is better described by r^{2}SCAN (see Fig. 1 in the main text). The green star represents an ideal generalized Kohn-Sham functional that would capture the experimental band gap for both $\alpha = 0$ and $\alpha = \varepsilon_\infty^{-1}$. Experimental inverse dielectric constants are used for DD-DFTH calculations. Note that the black circles and red squares do not share the same abscissa. Source data are provided as a Source Data file.

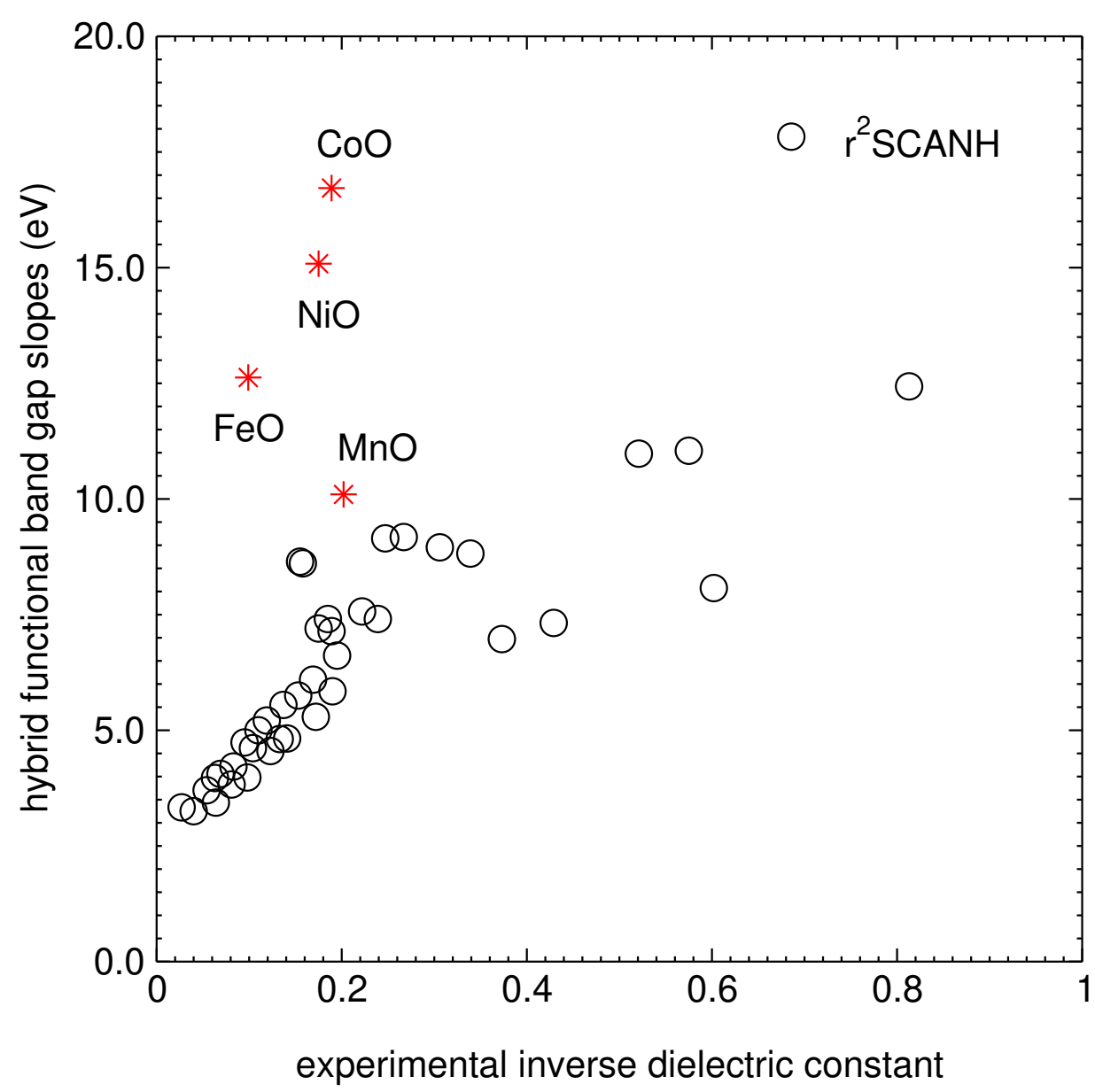

Supplementary Figure 6. **Strongly correlated transition metal oxides as challenging outliers.** DD-r^{2}SCANH hybrid functional slopes as in Fig. 6 in the main text, but here also including the rock salt transition metal monoxides MnO, FeO, CoO, and NiO (indicated as red stars). The large deviation from the trend given by conventional semiconductors can be interpreted as a sign of strong correlation, and elucidates difficulties of the DDH approach for these materials. Note the slightly different ordinate scale compared to Fig. 6 in the main text. Source data are provided as a Source Data file.

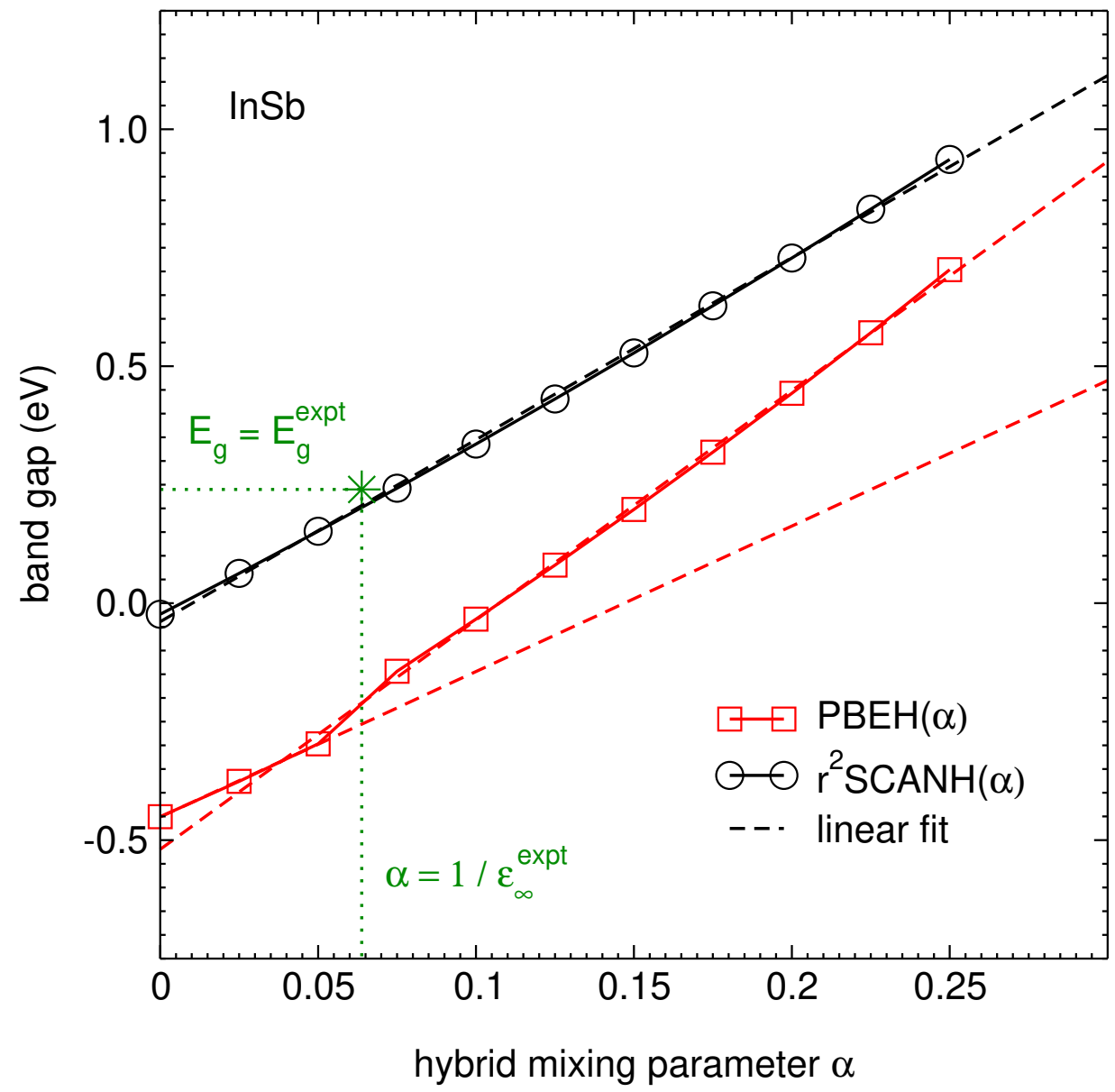

Supplementary Figure 7. **Case study for a challenging narrow-gap semiconductor.** Band gaps of InSb obtained with hybrid functionals PBEH and r^{2}SCANH as a function of the mixing parameter α. Similar to the case of InAs shown in Fig. 2(b) in the main text, r^{2}SCANH exhibits linear behavior, but PBEH deviates from linearity as there is an inversion of valence and conduction bands for $\alpha \lesssim 0.11$, see also Table I in the main text. Here, the two red dashed lines represent linear fits to small α data and large α data (regions of negative and positive band gaps, respectively). The fact that the experimental data (indicated by green stars) lie closer to the black lines than to the red lines signifies improvement of DD-r^{2}SCANH upon DD-PBEH. Source data are provided as a Source Data file.

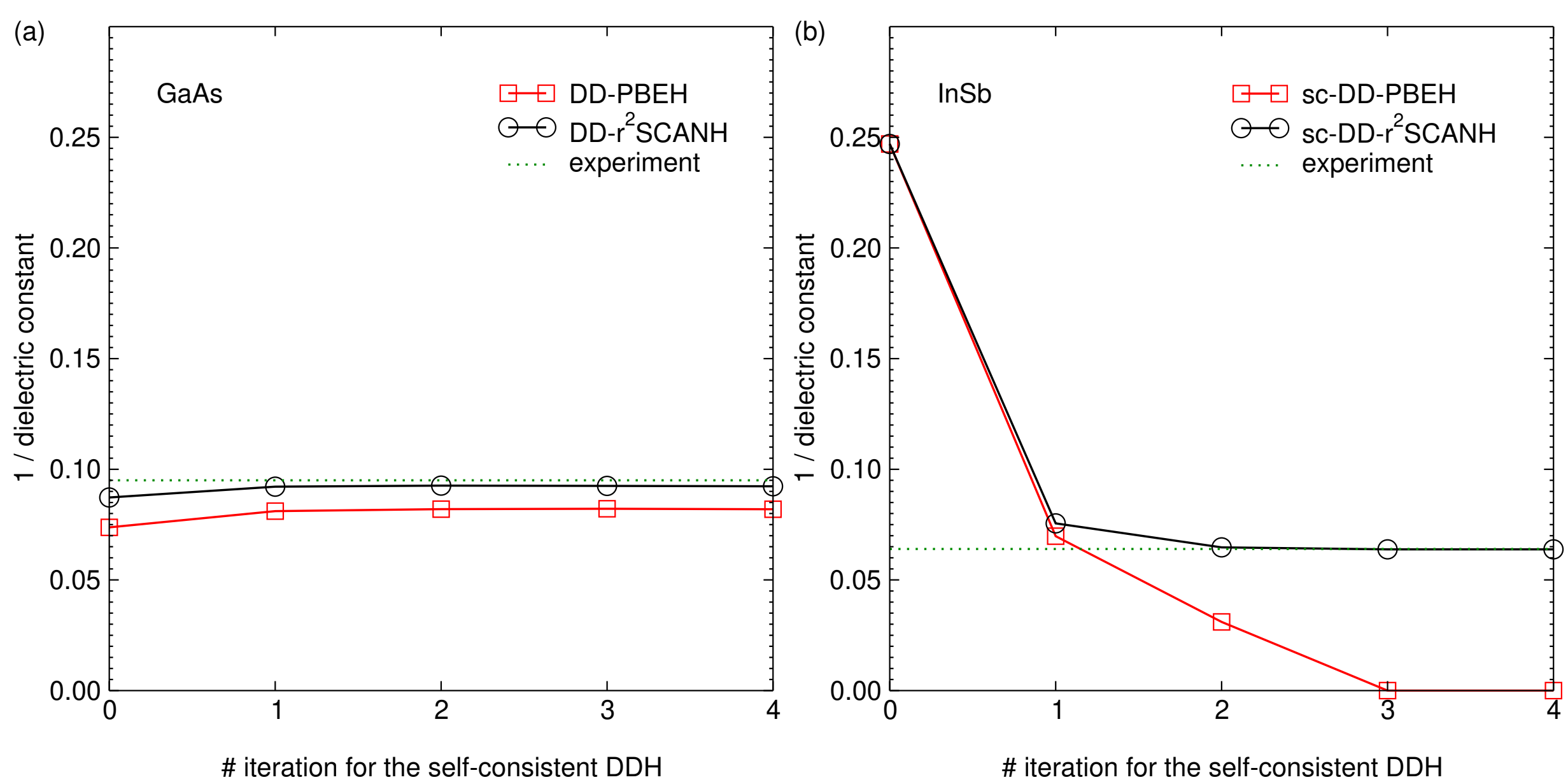

Supplementary Figure 8. **Self-consistent dielectric constants.** Evaluation of the dielectric constants following the self-consistent DDH scheme of Skone, Govoni, and Galli.[6] (a) GaAs, the respective DFT dielectric constants are used as starting point. (b) InSb, a mixing parameter of 1/4 is used as starting point. We apply a spin-orbit coupling correction of −0.010 to the calculated inverse dielectric constants, see Supplementary Note 8. We note that self-consistent DD-PBEH yields a false metallic ground state even without the inclusion of spin-orbit coupling, see also Ref. 14. Source data are provided as a Source Data file.

SUPPLEMENTARY TABLES

Supplementary Table 1. Characterization of the materials studied and technical settings used. Values for the experimental lattice constant a_0 (in Å) are taken from Ref. 7, unless stated otherwise. For wurtzite and rutile materials (space groups $P6_3mc$ and $P4_2/mnm$), c_0/a_0 ratios and internal parameters (u / x_2) are given as well. Short-hands _GW of the PAW potentials indicate an accurate treatment of high energy states. Moreover, short-hands _sv and _d indicate treatment of the outermost semi-core states as valence. Orbital plane wave cutoffs E_{cut} are given in eV. Throughout, we have increased E_{cut} beyond the default cutoff by a factor 1.4 .

	Space group	a_0	c_0/a_0	u / x_2	PAW potentials	E_{cut}
C	Fd$\bar{3}$m	3.567			C_GW_new	608
Si	Fd$\bar{3}$m	5.430			Si_sv_GW	767
Ge	Fd$\bar{3}$m	5.658			Ge_sv_GW	575
SiC	F$\bar{4}$3m	4.358			Si_sv_GW C_GW_new	767
BN	F$\bar{4}$3m	3.616			B_GW_new N_GW_new	634
AlN	P6_3mc	3.110[64]	1.601	0.3821	Al_sv_GW N_GW_new	634
AlP	F$\bar{4}$3m	5.460[65]			Al_sv_GW P_sv_GW*	576
AlAs	F$\bar{4}$3m	5.661			Al_sv_GW As_sv_GW	581
AlSb	F$\bar{4}$3m	6.136[18]			Al_sv_GW Sb_sv_GW	576
GaN	P6_3mc	3.190[18]	1.626	0.3789	Ga_sv_GW N_GW_new	634
GaP	F$\bar{4}$3m	5.448			Ga_sv_GW P_sv_GW*	566
GaAs	F$\bar{4}$3m	5.648			Ga_sv_GW As_sv_GW	581
GaSb	F$\bar{4}$3m	6.096[18]			Ga_sv_GW Sb_sv_GW	566
InN	P6_3mc	3.538[66]	1.612	0.3769	In_sv_GW N_GW_new	634
InP	F$\bar{4}$3m	5.866			In_sv_GW P_sv_GW*	514
InAs	F$\bar{4}$3m	6.058[18]			In_sv_GW P_sv_GW	581
InSb	F$\bar{4}$3m	6.479[18]			In_sv_GW Sb_sv_GW	522
MgO	Fm$\bar{3}$m	4.207			Mg_sv_GW O_GW_new	654
MnO	Fm$\bar{3}$m, AFM-II	4.445[55]			Mn_sv_GW O_GW_new	654
FeO	Fm$\bar{3}$m, AFM-II	4.334[55]			Fe_sv_GW O_GW_new	654
CoO	Fm$\bar{3}$m, AFM-II	4.254[55]			Co_sv_GW O_GW_new	654
NiO	Fm$\bar{3}$m, AFM-II	4.171[55]			Ni_sv_GW O_GW_new	654
CaO	Fm$\bar{3}$m	4.803			Ca_sv_GW O_GW_new	654
TiO_2	P4_2/mnm	4.587[67]	0.644	0.3047	Ti_sv_GW O_GW_new	654
Cu_2O	Pn$\bar{3}$m	4.269			Cu_sv_GW O_GW_new	654
ZnO	P6_3mc	3.250[18]	1.602	0.3819	Zn_sv_GW O_GW_new	654
ZnS	F$\bar{4}$3m	5.410			Zn_sv_GW S_GW	562
ZnSe	F$\bar{4}$3m	5.667			Zn_sv_GW Se_sv_GW	657
ZnTe	F$\bar{4}$3m	6.103[18]			Zn_sv_GW Te_sv_GW	562
CdO	Fm$\bar{3}$m	4.696[18]			Cd_sv_GW O_GW_new	654
CdS	P6_3mc	4.137[68]	1.624	0.3772	Cd_sv_GW S_GW	507
CdSe	P6_3mc	4.298[69]	1.631	0.3760	Cd_sv_GW Se_sv_GW	657
CdTe	F$\bar{4}$3m	6.481[18]			Cd_sv_GW Te_sv_GW	527
SnO_2	P4_2/mnm	4.737[70]	0.673	0.3056	Sn_sv_GW O_GW_new	654
PbS	Fm$\bar{3}$m	5.909[24]			Pb_d_GW S_sv_GW	362
PbSe	Fm$\bar{3}$m	6.098[24]			Pb_d_GW Se_sv_GW	657
PbTe	Fm$\bar{3}$m	6.428[24]			Pb_d_GW Te_sv_GW	527
LiF	Fm$\bar{3}$m	4.010			Li_sv_GW F_GW_new	672
LiCl	Fm$\bar{3}$m	5.106			Li_sv_GW Cl_GW	607
NaF	Fm$\bar{3}$m	4.609[65]			Na_sv_GW F_GW_new	683
NaCl	Fm$\bar{3}$m	5.595			Na_sv_GW Cl_GW	522
Ne	Fm$\bar{3}$m	4.464[71]			Ne_GW	605
Ar	Fm$\bar{3}$m	5.311[71]			Ar_GW	407

*P_sv_GW PAW potential as in Ref. 72, where the authors discussed that the inclusion of phosphor semicore states is required to obtain accurate band gaps for AlP.

Supplementary Table 2. Band gaps E_g for 39 semiconductors and insulators. Spin-orbit coupling corrections ΔSOC are calculated using r^{2}SCAN. For lead chalcogenides, additional corrections are applied to fully account for SOC at the all-electron level, see Supplementary Table 4. Zero-point renormalization correction (ΔZPR) are taken from Ref. 29 unless indicated otherwise. Mean signed relative errors (MSRE), mean absolute relative errors (MARE), and maximum relative errors (MAXRE) compare calculated band gaps (corrected for SOC and ZPR) with respective experimental values. To avoid strong statistical distortions caused by qualitative failures for narrow-gap semiconductors, we have limited the relative errors to −100%. Therefore, we refrain from counting negative band gaps as negative in the statistics. We also report mean signed errors (MSE), mean absolute errors (MAE), and maximum signed errors (MAXSE). Unless stated otherwise, experimental band gaps are taken from Ref. 7. E_g is given in eV and refers to the fundamental (indirect) gaps. CaO is an exception, as indirect experimental band gaps are currently unavailable.[73,74] We therefore compare the direct gap at Γ with the corresponding experimental value.[75] For the sake of completeness, the calculated fundamental band gaps for CaO are given as well. Experimental inverse dielectric constants $\varepsilon_\infty^{-1,\mathrm{expt}}$ are listed in Supplementary Table 5.

	PBE	r^{2}SCAN	HSE	DD-PBEH $\alpha=\varepsilon_\infty^{-1,\mathrm{expt}}$	DD-r^{2}SCANH $\alpha=\varepsilon_\infty^{-1,\mathrm{expt}}$	ΔSOC	ΔZPR	Expt.
C	4.16	4.37	5.34	5.49	5.63	−0.00	−0.33	5.48
Si	0.57	0.75	1.14	0.95	1.10	−0.02	−0.06	1.17
Ge	0.00	0.45	0.79	0.31	0.70	−0.10	−0.03	0.74
SiC	1.35	1.72	2.24	2.32	2.60	−0.00	−0.18	2.42
BN	4.46	4.98	5.80	6.31	6.66	−0.01	−0.41	6.36[76]
AlN	4.19	4.86	5.57	6.19	6.63	−0.00	−0.40	6.23
AlP	1.58	1.90	2.28	2.29	2.54	−0.02	−0.09	2.52
AlAs	1.44	1.79	2.09	2.06	2.35	−0.10	−0.07	2.24
AlSb	1.21	1.48	1.76	1.65	1.87	−0.21	−0.05	1.69[76]
GaN	1.93	2.35	3.20	3.37	3.70	−0.00	−0.19	3.51
GaP	1.59	1.84	2.28	2.19	2.39	−0.03	−0.07	2.35
GaAs	0.54	1.04	1.44	1.05	1.49	−0.11	−0.02	1.52
GaSb	0.12	0.55	0.92	0.44	0.83	−0.22	−0.03[27]	0.82[76]
InN	−0.22	0.09	0.72	0.44	0.71	−0.00	−0.07[27]	0.70[23]
InP	0.70	1.15	1.49	1.25	1.63	−0.03	−0.03[30]	1.42
InAs	−0.23	0.22	0.50	0.12	0.53	−0.11	−0.02[27]	0.42[76]
InSb	−0.19	0.24	0.50	0.07	0.46	−0.24	−0.02[27]	0.24[76]
MgO	4.77	5.67	6.53	8.16	8.66	−0.01	−0.52	7.83
CaO‡ ($E_g^{\Gamma\to\Gamma}$)	4.75	5.45	6.29	7.50	7.90	−0.01	−0.42[30]	7.09[75]
CaO‡	3.65	4.20	5.34	6.61	6.94	−0.01	−0.34	N/A‡
TiO_2	1.89	2.22	3.41	3.27	3.58	−0.00	−0.34	3.03[23]
Cu_2O	0.53	0.76	2.01	1.87	2.10	−0.09	−0.03[7]	2.17
ZnO	0.81	1.24	2.55	3.38	3.69	−0.02	−0.16	3.44
ZnS	2.09	2.66	3.33	3.55	3.95	−0.02	−0.09	3.84
ZnSe	1.29	1.88	2.43	2.45	2.91	−0.12	−0.04	2.82
ZnTe	1.24	1.81	2.27	2.10	2.57	−0.28	−0.02	2.39[76]
CdO	−0.41	0.11	0.93	1.03	1.48	−0.02	−0.08†	1.09[77]
CdS	1.22	1.69	2.26	2.47	2.80	−0.01	−0.07	2.58
CdSe	0.67	1.19	1.64	1.71	2.10	−0.12	−0.03	1.85
CdTe	0.76	1.27	1.64	1.55	1.95	−0.27	−0.02	1.61[76]
SnO_2	1.25	1.92	3.04	3.69	4.18	−0.00	−0.22	3.60
PbS	0.32	0.58	0.77	0.52	0.78	−0.47*	−0.00*	0.29[23]
PbSe	0.25	0.55	0.68	0.39	0.68	−0.52*	−0.00*	0.17[76]
PbTe	0.70	0.98	1.11	0.79	1.07	−0.87*	−0.00*	0.19[76]
LiF	9.18	10.16	11.52	15.66	15.88	−0.02	−1.13[30]	14.2
LiCl	6.40	7.35	7.82	9.58	9.95	−0.04	−0.44[30]	9.4
NaF	6.41	7.00	8.63	13.36	13.35	−0.02	−0.70[30]	11.70[23]
NaCl	5.21	5.85	6.63	8.88	8.99	−0.04	−0.30[30]	8.75[23]
Ne	11.59	13.00	14.39	23.41	23.11	−0.04	−0.08†	21.48[23]
Ar	8.67	9.57	10.35	14.56	14.43	−0.06	−0.02†	14.15[23]
MSRE	−63%	−46%	−15%	−26%	−1%			
MARE	63%	46%	16%	27%	7%			
MAXSRE	−100%	−100%	−34%	−100%	29%			
MSE	−2.03 eV	−1.59 eV	−0.93 eV	−0.20 eV	+0.09 eV			
MAE	2.03 eV	1.59 eV	0.94 eV	0.37 eV	0.19 eV			
MAXSE	−10.01 eV	−8.60 eV	−7.21 eV	+1.81 eV	+1.51 eV			

*SOC corrections for PbX are further detailed in Supplementary Table 4), ZPR is negligible according to Ref. 78.
†ZPR corrections for CdO, Ar and Ne are calculated here using the special displacement method.[31,32] Our ZPR calculations employ the r^{2}SCAN functional, a 128 atom supercell for CdO and 64 atom supercells for Ar and Ne, respectively.
‡The fundamental band gap of CaO has not been determined experimentally,
thus the direct band gap $E_g^{\Gamma\to\Gamma}$ is used for statistical analysis.

Supplementary Table 3. Continuation of Supplementary Table 2 for various exchange-correlation functionals. GWΓ results (high-level many-body perturbation theory) for 24 materials are taken from Ref. 18. Band gaps calculated for 30 materials with a range-separated DDH (sc-DSH) are taken from Ref. 14. Band gaps calculated for 15 materials with a different range-separated DDH (WOT-RSHS)[79] are taken from Ref. 80. For easier comparison, we have subtracted spin-orbit coupling (SOC) corrections using the values listed in Supplementary Table 2 from the WOT-RSHS+SOC band gaps given in Ref. 80.

	GWΓ Ref. 18	sc-DSH Ref. 14	WOT-SRSH Ref.80	LDA	TPSS	r^{2}SCAN-L	LAK	DD-LDAH $\alpha=\varepsilon_\infty^{-1,\text{expt}}$	DD-TPSSH $\alpha=\varepsilon_\infty^{-1,\text{expt}}$	DD-r^{2}SCAN-LH $\alpha=\varepsilon_\infty^{-1,\text{expt}}$	DD-LAKH $\alpha=\varepsilon_\infty^{-1,\text{expt}}$
C	5.92	5.66	5.76	4.12	4.20	4.27	4.74	5.53	5.53	5.58	6.03
Si	1.37	1.16	1.16	0.47	0.66	0.76	1.10	0.88	1.05	1.12	1.45
Ge	0.94	0.83	0.79	0.00	0.12	0.07	0.82	0.19	0.44	0.37	1.05
SiC	2.76	2.55		1.31	1.33	1.63	2.12	2.35	2.30	2.55	3.01
BN	6.81	6.60		4.35	4.47	4.82	5.50	6.34	6.32	6.57	7.15
AlN		6.25	6.56	4.24	4.19	4.52	5.37	6.31	6.18	6.44	7.03
AlP	2.74	2.51	2.54	1.45	1.64	1.84	2.38	2.22	2.35	2.51	3.00
AlAs	2.56	2.32	2.35	1.35	1.49	1.73	2.17	2.02	2.11	2.31	2.70
AlSb	2.06	2.09	1.92	1.15	1.28	1.44	1.65	1.62	1.71	1.86	2.07
GaN	3.75	3.57*		1.96	1.85	2.04	2.75	3.42	3.30	3.49	3.99
GaP	2.56	2.46	2.42	1.44	1.64	1.81	2.25	2.07	2.22	2.35	2.76
GaAs	1.66	1.62	1.52	0.35	0.67	0.80	1.61	0.86	1.20	1.29	1.97
GaSb	1.06	1.09	0.91	−0.05	0.26	0.40	0.88	0.26	0.59	0.70	1.12
InN		0.84		−0.23	−0.29	−0.13	0.38	0.44	0.37	0.56	0.97
InP	1.58	1.63	1.59	0.49	0.84	0.96	1.69	1.03	1.38	1.48	2.09
InAs	0.62	0.66	0.53	−0.43	−0.10	0.08	0.72	0.07	0.26	0.41	0.97
InSb	0.59	0.70	0.56	−0.38	−0.03	0.15	0.52	0.05	0.24	0.40	0.70
MgO	8.20	8.70	8.17	4.71	4.87	5.35	5.94	8.25	8.23	8.51	8.69
CaO ($E_g^{\Gamma\to\Gamma}$)				4.61	4.79	5.09	5.67	7.51	7.55	7.70	7.93
TiO_2				1.80	2.00	1.98	2.34	3.18	3.39	3.37	3.68
Cu_2O				0.53	0.55	0.69	0.94	1.89	1.88	2.04	2.27
ZnO	3.88	4.19		0.76	0.77	0.94	1.46	3.40	3.38	3.50	3.74
ZnS	3.93	4.04		1.87	2.25	2.31	3.13	3.37	3.70	3.72	4.25
ZnSe	3.07	3.08		1.10	1.49	1.63	2.36	2.28	2.65	2.73	3.23
ZnTe	2.72	2.31		1.04	1.44	1.57	2.11	1.91	2.31	2.38	2.73
CdO	1.33			−0.49	−0.41	−0.10	0.42	0.97	1.02	1.31	1.67
CdS	2.62*	2.84		0.96	1.42	1.45	2.05	2.24	2.66	2.65	3.00
CdSe	2.05*	2.17		0.45	0.90	1.03	1.57	1.50	1.94	2.00	2.32
CdTe	2.00	1.68		0.54	0.99	1.12	1.51	1.33	1.77	1.85	2.05
SnO_2				1.15	1.42	1.59	2.28	3.63	3.84	3.98	4.31
PbS		0.70		0.17	0.31	0.58	0.98	0.38	0.52	0.78	1.15
PbSe		0.64		0.14	0.28	0.57	0.91	0.29	0.42	0.70	1.03
PbTe		0.81		0.60	0.74	0.99	1.18	0.70	0.84	1.08	1.25
LiF		16.45	15.36	8.95	9.33	9.75	10.12	15.75	15.77	15.79	15.41
LiCl				6.07	6.59	6.98	7.78	9.50	9.72	9.81	10.00
NaF				6.11	6.76	6.68	6.74	13.36	13.54	13.30	12.68
NaCl		8.84		4.83	5.52	5.61	5.93	8.75	9.08	8.94	8.72
Ne				11.42	12.23	12.31	12.84	23.34	23.52	23.20	22.24
Ar				8.16	9.32	9.19	9.25	14.36	14.84	14.40	13.48

*) Calculations for CdS and CdSe used the zincblende structure in Ref. 18, and Ref. 14 used the zincblende structure for GaN, whereas the experimental crystal structure is wurtzite for all three semiconductors. Comparing HSE band gaps reported in Refs. 18 and 14 for zincblende structures with our own HSE calculations for wurtzite structures, we estimate that the zincblende band gaps are too small by 0.07 eV (CdS), 0.06 eV (CdSe), and 0.23 eV (GaN).

Supplementary Table 4. Spin-orbit coupling correction ΔSOC for the band gaps of lead chalcogenides PbS, PbSe, and PbTe. ΔSOC is evaluated as $E_g^{\mathrm{DFT+SOC}} - E_g^{\mathrm{DFT}}$ for the PBE, r^{2}SCAN, and HSE functionals. PBE yields erroneous inversion of valence and conduction bands at the L point, which is interpreted as a negative band gap $E_g^{\mathrm{DFT+SOC}}$, see Refs. 24 and 78. By comparing to the all-electron PBE calculations from Ref. 24 (FP-APW+LO+rel. LO), we obtain a correction $\Delta\mathrm{SOC}^{\mathrm{FP-APW}} - \Delta\mathrm{SOC}^{\mathrm{PAW}}$, which is added to our PAW calculations to obtain best estimates for ΔSOC. The SOC corrections used in Supplementary Table 2 are indicated in bold. All energies are given in eV.

	PBE	r^{2}SCAN	HSE
PbS			
E_g^{DFT} PAW (present work)	0.32	0.58	0.77
$E_g^{\mathrm{DFT+SOC}}$ PAW (present work)	−0.06	0.17	0.37
ΔSOC PAW present work	−0.38	−0.41	−0.40
ΔSOC PAW (Ref. 24)	−0.38		
ΔSOC FP-APW+LO+rel. LO (Ref. 24)	−0.44		
$\Delta\mathrm{SOC}^{\mathrm{FP-APW}}$-$\Delta\mathrm{SOC}^{\mathrm{PAW}}$	−0.06		
ΔSOC Best estimate	−0.44	**−0.47**	−0.46
PbSe			
E_g^{DFT} PAW (present work)	0.25	0.55	0.68
$E_g^{\mathrm{DFT+SOC}}$ PAW (present work)	−0.17	0.08	0.22
ΔSOC PAW present work	−0.42	−0.47	−0.46
ΔSOC PAW (Ref. 24)	−0.42		
ΔSOC FP-APW+LO+rel. LO (Ref. 24)	−0.47		
$\Delta\mathrm{SOC}^{\mathrm{FP-APW}}$-$\Delta\mathrm{SOC}^{\mathrm{PAW}}$	−0.05		
ΔSOC Best estimate	−0.47	**−0.52**	−0.51
PbTe			
E_g^{DFT} PAW (present work)	0.70	0.98	1.11
$E_g^{\mathrm{DFT+SOC}}$ PAW (present work)	−0.04	0.15	0.27
ΔSOC PAW present work	−0.74	−0.85	−0.84
ΔSOC PAW (Ref. 24)	−0.74		
ΔSOC FP-APW+LO+rel. LO (Ref. 24)	−0.76		
$\Delta\mathrm{SOC}^{\mathrm{FP-APW}}$-$\Delta\mathrm{SOC}^{\mathrm{PAW}}$	−0.02		
ΔSOC Best estimate	−0.76	**−0.87**	−0.86

Supplementary Table 5. Inverse dielectric constants ε_∞^{-1} for 39 semiconductors and insulators. For non-cubic materials, we report the inverse of the trace-average of the dielectric tensor, that is $\varepsilon_\infty^{-1} = 3/(\varepsilon_{\infty,xx} + \varepsilon_{\infty,yy} + \varepsilon_{\infty,zz})$. We use finite electric fields,[1,2] see Supplementary Note 1 for details. The numerical accuracy of our approach is demonstrated by comparing PBE results for ε_∞^{-1} obtained with finite electric fields (PEAD, see Supplementary Note 1) and density functional perturbation theory[13] (DFPT). Calculated inverse dielectric constants are corrected by spin-orbit coupling at the r^{2}SCAN level, see Eq. (7). We have limited the relative errors to −100%, and thus refrain from counting some PBE inverse dielectric constants as negative in the statistics. Mean signed relative errors (MSRE), mean absolute relative errors (MARE), and maximum signed relative errors (MAXSRE) compare corrected ε_∞^{-1} with respective experimental values. Unless stated otherwise, experimental dielectric constants are taken from Ref. 7 (see last column).

	PBE DFPT	PBE PEAD	r^{2}SCAN PEAD	ΔSOC	Expt.	$\varepsilon_\infty^{\mathrm{expt}}$
C	0.171	0.171	0.173	−0.000	0.175	5.70
Si	0.077	0.077	0.085	−0.000	0.083	12.0
Ge	0.000	0.000	0.059	−0.001	0.063	15.9
SiC	0.143	0.143	0.153	−0.000	0.153	6.52
BN	0.217	0.217	0.224	−0.000	0.222	4.5
AlN	0.219	0.219	0.237	−0.000	0.239	4.18
AlP	0.123	0.123	0.136	−0.000	0.133	7.54
AlAs	0.107	0.107	0.122	−0.001	0.123	8.16
AlSb	0.086	0.086	0.099	−0.000	0.098	10.24[26]
GaN	0.171	0.171	0.184	−0.000	0.189	5.30
GaP	0.097	0.097	0.107	−0.000	0.110	9.1
GaAs	0.074	0.074	0.087	−0.000	0.095	10.58
GaSb	0.050	0.050	0.067	−0.003	0.069	14.4[81]
InN	0.000	0.000	0.075	−0.000	0.119	8.4[26]
InP	0.091	0.092	0.105	−0.000	0.104	9.6
InAs	0.000	0.000	0.076	−0.005	0.081	12.37[26]
InSb	0.000	0.000	0.066	−0.066*	0.064	15.68[26]
MgO	0.312	0.312	0.333	−0.000	0.339	2.95[82]
CaO	0.264	0.264	0.284	−0.000	0.306	3.27[82]
TiO_2	0.128	0.128	0.143	−0.000	0.158	6.34
Cu_2O	0.109	0.109	0.121	−0.000	0.155	6.46
ZnO	0.197	0.198	0.228	−0.000	0.267	3.74
ZnS	0.168	0.169	0.185	−0.000	0.195	5.13
ZnSe	0.138	0.138	0.157	−0.000	0.169	5.9
ZnTe	0.112	0.112	0.128	−0.002	0.137	7.28[26]
CdO	0.000	0.000	0.172	−0.000	0.185	5.4[83]
CdS	0.166	0.166	0.186	−0.000	0.190	5.25
CdSe	0.134	0.134	0.159	−0.002	0.172	5.8
CdTe	0.115	0.115	0.134	−0.003	0.141	7.1
SnO_2	0.224	0.224	0.246	−0.000	0.247	4.05
PbS	0.057	0.057	0.068	−0.020	0.054	18.4[25]
PbSe	0.044	0.044	0.055	−0.024	0.040	25.2[25]
PbTe	0.034	0.034	0.041	−0.015	0.027	36.9[25]
LiF	0.484	0.484	0.503	−0.000	0.521	1.92[82]
LiCl	0.336	0.336	0.354	−0.000	0.373	2.68[82]
NaF	0.531	0.531	0.546	−0.000	0.575	1.74[82]
NaCl	0.396	0.395	0.410	−0.000	0.429	2.33[82]
Ne	0.782	0.781	0.793	−0.000	0.813	1.23[6]
Ar	0.582	0.582	0.591	−0.000	0.602	1.66
MSRE	−26%	−26%	−9%			
MARE	26%	26%	9%			
MAXSRE	−100%	−100%	−100%			

*In Supplementary Note 8 we discuss that r^{2}SCAN likely overestimates the SOC correction for InSb, a better estimate is −0.010.

Supplementary Table 6. Ionization potentials for 24 semiconductors and insulators. Reference values for PBE and HSE are taken from Grüneis et al.[18] Ionization potential for other exchange-correlation functionals are obtained by adding bulk valence band shifts to HSE lineups [Eq. (5)]. GWΓ results (high-level many-body perturbation theory) are taken from Ref. 18. Spin-orbit corrections ΔSOC are calculated using r^{2}SCAN. Zero-point renormalization corrections ΔZPR for the ionization potentials are taken from Ref. 29 unless indicated otherwise. Mean signed relative errors (MSRE), mean absolute relative errors (MARE), and maximum signed relative errors (MAXRE) compare corrected band gaps with average experimental values. We also report mean signed errors (MSE), mean absolute errors (MAE), and maximum signed errors (MAXSE). All energies are given in eV. Unless stated otherwise, experimental ionization potentials are taken from Ref. 18. Experimental inverse dielectric constants $\varepsilon_\infty^{-1,\mathrm{expt}}$ are listed in Supplementary Table 5.

	PBE Ref. 18	r^{2}SCAN	HSE Ref. 18	DD-PBEH $\alpha=\varepsilon_\infty^{-1,\mathrm{expt}}$	DD-r^{2}SCANH $\alpha=\varepsilon_\infty^{-1,\mathrm{expt}}$	GWΓ Ref. 18	ΔSOC	ΔZPR	Expt.
C	5.58	5.58	6.18	6.23	6.24	6.74	−0.00	−0.13	5.85, 6.00, 6.5
Si	4.82	4.94	5.22	5.09	5.13	5.49	−0.02	−0.04	5.15, 5.33
Ge	4.43	4.57	4.73	4.55	4.70	5.17	−0.10	−0.02	4.75, 4.80
SiC†	6.03	6.13	6.64	6.62	6.69	7.00	−0.00	−0.11	5.90, 6.00
BN	7.03	7.15	7.85	8.08	8.18	8.52	−0.01	−0.20	
AlP	5.70	5.91	6.22	6.17	6.29	6.64	−0.02	−0.05	
AlAs	5.32	5.60	5.83	5.75	5.92	6.37	−0.10	−0.04	
AlSb	4.80	4.96	5.17	5.05	5.18	5.58	−0.22	−0.02	5.22
GaN	5.92	6.05	6.61	6.67	6.87	7.12	−0.00	−0.10	6.60, 6.80
GaP	5.26	5.49	5.75	5.64	5.78	6.13	−0.03	−0.03	5.95, 6.01
GaAs	4.84	5.15	5.32	5.17	5.38	5.77	−0.11	−0.02	5.40, 5.47, 5.56
GaSb	4.40	4.70	4.86	4.68	4.85	5.24	−0.22	−0.02‡	4.70, 4.76, 4.94
InP	5.13	5.34	5.59	5.47	5.61	5.95	−0.03	−0.02‡	5.69, 5.85
InAs	4.77	5.06	5.22	5.04	5.24	5.67	−0.11	−0.01‡	4.90, 5.31, 5.42
InSb	4.44	4.72	4.88	4.69	4.86	5.28	−0.23	−0.01‡	4.77, 4.90
MgO	5.20	5.29	6.15	7.13	7.36	7.34	−0.01	−0.32	7.15
ZnO	6.01	6.18	7.11	7.61	7.88	8.19	−0.02	−0.11	7.82[18] 7.80[17]
ZnS	6.03	6.31	6.77	6.88	7.08	7.43	−0.02	−0.05	7.50
ZnSe	5.62	5.95	6.29	6.30	6.54	7.06	−0.12	−0.03	6.82
ZnTe	5.09	5.34	5.61	5.54	5.74	6.19	−0.28	−0.02	5.76
CdO	4.82	4.68	5.40	5.39	5.67	5.78	−0.02	−0.08§	5.70
CdS*	5.92	6.21	6.60	6.70	6.90	7.17	−0.01	−0.04	7.26[17]
CdSe*	5.61	5.94	6.23	6.26	6.50	6.93	−0.12	−0.03	6.62
CdTe	5.17	5.44	5.68	5.63	5.83	6.24	−0.27	−0.02	5.78 5.8
MSRE	−16%	−12%	−6%	−7%	−4%	+3%			
MARE	16%	12%	6%	7%	4%	3%			
MAXSRE	−32%	−31%	−19%	−10%	−7%	+8%			
MSE	−0.99 eV	−0.78 eV	−0.41 eV	−0.39 eV	−0.21 eV	+0.14 eV			
MAE	0.99 eV	0.78 eV	0.41 eV	0.39 eV	0.22 eV	0.19 eV			
MAXSE	−2.28 eV	−2.19 eV	−1.33 eV	−0.69 eV	−0.49 eV	+0.49 eV			

*CdS and CdSe were calculated as zincblende in Ref. 18, whereas the experimental crystal structure is wurtzite. However, the PBE ionization potentials of the two phases differ by less than 0.1 eV for CdS (see Ref. 17).

†Following Refs. 16 and 19, we have excluded SiC from the statistical analysis. The experimental ionization potential is measured for a reconstructed polar SiC (100) surface, whereas the nonpolar SiC (110) surface was modelled in Ref. 18.

‡ZPR for GaSb and InX is approximated as 50% of the band gap ZPR corrections listed in Supplementary Table 2, thus $\Delta \mathrm{IP}^{\mathrm{ZPR}} \approx \Delta \mathrm{E}_\mathrm{g}^{\mathrm{ZPR}}/2$.

§ZPR for CdO is calculated here using the special displacement method,[31,32] using r^{2}SCAN and a 128 atom supercell.

Supplementary Table 7. Hole effective masses m_p and electron masses m_n of six common semiconductors (in electron masses m_0) are obtained by non-linear fits [see Eq. (16) in the main text].[57,84] Per convention, all effective masses are reported as positive. Throughout, we report effective masses at the Γ point along the [100] direction. For all six materials considered here, spin-orbit coupling introduces a splitting of the valence band into a three bands which are commonly denoted heavy hole, light hole, and split-off band. The corresponding hole effective masses are denoted $m_{p,\mathrm{hh}}$, $m_{p,\mathrm{lh}}$ and $m_{p,\mathrm{so}}$, respectively. Mean signed relative errors (MSRE), mean absolute relative errors (MARE), and maximum signed relative errors (MAXSRE) are given with respect to the experimental values. A false metallic ground state can qualitatively alter the DFT band dispersions, leading to large errors for the calculated effective masses. In such cases (marked by daggers), we have limited the relative errors to ±100%. Experimental inverse dielectric constants $\varepsilon_\infty^{-1,\mathrm{expt}}$ are listed in Supplementary Table 5.

		PBE	$\mathrm{r^2SCAN}$	HSE	DD-PBEH $\alpha{=}\varepsilon_\infty^{-1,\mathrm{expt}}$	DD-$\mathrm{r^2SCANH}$ $\alpha{=}\varepsilon_\infty^{-1,\mathrm{expt}}$	Expt.
Ge	$m_{p,\mathrm{so}}$	0.029†	0.070	0.092	0.060	0.085	0.095[26]
	$m_{p,\mathrm{lh}}$	0.006†	0.023	0.041	0.014	0.035	0.044[26]
	$m_{p,\mathrm{hh}}$	0.205	0.194	0.205	0.207	0.197	0.284[26]
	m_n	0.007†	0.019	0.032	0.013	0.028	0.038[26]
GaAs	$m_{p,\mathrm{so}}$	0.109	0.146	0.162	0.141	0.162	0.16[85]
	$m_{p,\mathrm{lh}}$	0.035	0.067	0.079	0.061	0.081	0.09[85]
	$m_{p,\mathrm{hh}}$	0.323	0.320	0.314	0.321	0.310	0.395[85]
	m_n	0.029	0.053	0.063	0.048	0.064	0.066[85]
GaSb	$m_{p,\mathrm{so}}$	0.088	0.121	0.139	0.113	0.134	0.15[26]
	$m_{p,\mathrm{lh}}$	0.010†	0.023	0.041	0.015	0.038	0.042[26]
	$m_{p,\mathrm{hh}}$	0.232	0.226	0.232	0.234	0.227	0.29[26]
	m_n	0.010†	0.022	0.037	0.014	0.034	0.039[26]
InP	$m_{p,\mathrm{so}}$	0.142	0.181	0.192	0.174	0.194	0.21[85]
	$m_{p,\mathrm{lh}}$	0.074	0.107	0.115	0.101	0.120	0.12[85]
	$m_{p,\mathrm{hh}}$	0.434	0.423	0.411	0.425	0.414	0.565[85]
	m_n	0.055	0.080	0.088	0.075	0.091	0.079[85]
InAs	$m_{p,\mathrm{so}}$	0.001†	0.076	0.106	0.048	0.108	0.140[28]
	$m_{p,\mathrm{lh}}$	0.002†	0.010	0.031	0.008†	0.033	0.027[28]
	$m_{p,\mathrm{hh}}$	0.363	0.360	0.344	0.007†	0.353	0.333[28]
	m_n	0.038	0.010	0.025	0.025	0.027	0.026[28]
InSb	$m_{p,\mathrm{so}}$	0.056	0.108	0.127	0.081	0.125	0.110[28]
	$m_{p,\mathrm{lh}}$	0.124†	0.001†	0.017	0.027†	0.016	0.015[28]
	$m_{p,\mathrm{hh}}$	0.255	0.254	0.251	0.037†	0.252	0.263[28]
	m_n	0.044†	0.001†	0.016	0.038†	0.016	0.014[28]
MSRE		−32%	−32%	−5%	−29%	−6%	
MARE		53%	32%	11%	44%	13%	
MAXSRE		100%	−93%	28%	100%	31%	

†Some effective masses show large deviations from experiment due to false metallic DFT ground states.
The effective mass fits for InAs and InSb obtained with DD-PBEH show significant errors,
as the appearance metal-insulator transition leads to large numerical noise in the calculated band structures.

Supplementary Table 8. Equilibrium lattice constants a_0 for 18 strongly bonded solids are obtained by a third order Birch-Murnaghan equation-of-state fit [Eq. (17) in the main text].[86] Experimental lattice constants a_0 and ZPR corrections for a_0 are taken from Ref. 7, unless stated otherwise. Mean signed relative errors (MSRE), mean absolute relative errors (MARE), and maximum signed relative errors (MAXRE) are given with respect to the experimental values. All lattice constants are given in Å. Experimental inverse dielectric constants $\varepsilon_\infty^{-1,\mathrm{expt}}$ are listed in Supplementary Table 5.

	PBE	r^2SCAN	HSE	DD-PBEH $\alpha=\varepsilon_\infty^{-1,\mathrm{expt}}$	DD-r^2SCANH $\alpha=\varepsilon_\infty^{-1,\mathrm{expt}}$	ΔZPR	Expt.
C	3.572	3.559	3.545	3.552	3.545	0.012	3.567
Si	5.472	5.448	5.442	5.460	5.441	0.008	5.430
Ge	5.764	5.684	5.683	5.739	5.671	0.008[65]	5.658
SiC	4.382	4.360	4.350	4.361	4.347	0.010	4.358
BN	3.625	3.609	3.596	3.599	3.591	0.015[65]	3.616
AlP	5.507	5.471	5.476	5.489	5.464	0.009[65]	5.460[65]
AlAs	5.728	5.672	5.683	5.703	5.663	0.009[65]	5.661
GaP	5.507	5.454	5.466	5.485	5.446	0.009[65]	5.448
GaAs	5.747	5.671	5.684	5.718	5.659	0.007	5.648
InP	5.961	5.909	5.903	5.932	5.893	0.008[65]	5.866
InAs	6.189	6.112	6.109	6.158	6.096	0.007[65]	6.058[18]
InSb	6.637	6.557	6.548	6.609	6.542	0.006[65]	6.479[18]
MgO	4.255	4.207	4.203	4.187	4.167	0.019	4.207
CaO	4.827	4.791	4.794	4.786	4.772	0.022	4.803
LiF	4.066	3.993	4.010	3.951	3.931	0.036	4.010
LiCl	5.150	5.117	5.114	5.090	5.085	0.034	5.106
NaF	4.708	4.599	4.646	4.571	4.540	0.027[65]	4.609[65]
NaCl	5.700	5.593	5.658	5.621	5.577	0.030	5.595
MSRE	+1.6%	+0.5%	+0.6%	+0.7%	+0.1%		
MARE	1.6%	0.5%	0.7%	0.8%	0.4%		
MAXSRE	+2.7%	+1.3%	+1.7%	+2.1%	−1.1%		

Supplementary Table 9. Inverse dielectric constants ε_∞^{-1} for four rock salt transition metal monoxides. The numerical accuracy of our approach is demonstrated by comparing PBE results for ε_∞^{-1} obtained with finite electric fields (PEAD, see Supplementary Note 1) and density functional perturbation theory[13] (DFPT). Experimental dielectric constants are taken from Ref. 58, unless indicated otherwise (see last column). Where multiple experimental references are available, we take an average value for $\varepsilon_\infty^{-1,\mathrm{expt}}$ (second-to-last column). For comparison, we also give literature results obtained using HSE,[55] and the Bethe-Salpeter equation (BSE) with various starting points from many-body perturbation theory (MBPT).[27,58]

	PBE DFPT	PBE PEAD	r^2SCAN PEAD	HSE PEAD	MBPT BSE	Expt.	$\varepsilon_\infty^{\mathrm{expt}}$
MnO	0.126	0.126	0.180	0.220[55]	0.210,[27] 0.236,[58] 0.244[58]	0.202	4.95
FeO	0.000	0.000	0.080	0.196[55]	0.057,[27] 0.173,[58] 0.188[58]	0.099	9.24, 11.1
CoO	0.000	0.000	0.137	0.207[55]	0.176,[58] 0.193,[58] 0.194[27]	0.189	5.30
NiO	0.059	0.059	0.139	0.198[55]	0.160,[58] 0.163,[27] 0.176[58]	0.175	5.43, 5.70, 6.0

Supplementary Table 10. Band gaps E_g for four rock salt transition metal monoxides. Spin-orbit correction (ΔSOC), zero-point renormalization corrections (ΔZPR), and experimental band gaps are taken from Ref. 58, unless indicated otherwise. For comparison, we also give literature results obtained using many-body perturbation theory (MBPT).[18,27,58] All calculated band gaps are given without SOC and ZPR effects. That is, ΔSOC and ΔZPR should be added to the calculated band gaps before comparing to experimental values. E_g is given in eV and always refers to the fundamental (indirect) gaps. False metallic ground state are indicated as "met". For DDH functionals, we indicate whether the experimental inverse dielectric constants or the inverse dielectric constants calculated with the respective DFT base functionals are used, see Supplementary Table 9 for the numerical values.

	PBE	r^2SCAN	HSE	DD-PBEH		DD-r^2SCANH		MBPT	ΔSOC	ΔZPR	Expt.
				$\varepsilon_\infty^{-1,\mathrm{expt}}$	$\varepsilon_\infty^{-1,\mathrm{PBE}}$	$\varepsilon_\infty^{-1,\mathrm{expt}}$	$\varepsilon_\infty^{-1,r^2\mathrm{SCAN}}$				
MnO	0.85	1.68	2.92	3.09	2.25	3.72	3.50	3.05,[27] 3.91,[58] 4.32[58]	−0.00[58]	−0.30[58]	3.9, 4.1
FeO	met	0.37	2.11	1.18	met	1.62	1.38	0.67,[27] 2.58,[58] 2.73[58]	+0.27[58]	−0.18[58]	1.05,* 2.15†
CoO	met	0.90	3.53	3.38	met	4.06	3.19	3.28,[27] 3.49,[58] 3.81[58]	−0.00[58]	−0.28[58]	2.5, 2.6, 3.6
NiO	0.97	2.43	4.56	4.10	2.03	5.07	4.53	3.23,[27] 3.75,[58] 4.14[58]	−0.00[58]	−0.25[58]	4.00, 4.30

*Reinterpretation of experimental data combined with a model MBPT calculation.[27]

†From a linear extrapolation of an experimental absorption spectra.[58]

Supplementary Table 11. Local magnetic moments of the transition metal atoms μ for four rock salt transition metal monoxides. Experimental band gaps are taken from Ref. 58. For comparison, we also give literature results obtained using many-body perturbation theory (MBPT).[27,58] For DDH functionals, the experimental inverse dielectric constants are used, see Supplementary Table 9 for the numerical values. All magnetic moments are given in μ_B per atom.

	PBE	$\mathrm{r^2SCAN}$	HSE	DD-PBEH $\alpha=\varepsilon_\infty^{-1,\mathrm{expt}}$	DD-$\mathrm{r^2SCANH}$ $\alpha=\varepsilon_\infty^{-1,\mathrm{expt}}$	MBPT	Expt.
MnO	4.20	4.34	4.41	4.38	4.44	4.43,[58] 4.46,[58] 4.73[27]	4.58
FeO	3.40	3.52	3.59	3.50	3.56	3.57,[58] 3.60,[58] 3.66[27]	3.32, 4.20
CoO	2.42	2.57	2.66	2.63	2.68	2.65,[58] 2.66,[58] 2.70[27]	2.46
NiO	1.38	1.60	1.70	1.65	1.72	1.67,[58] 1.67,[58] 1.67[27]	1.64, 1.90

SUPPLEMENTARY REFERENCES

[1] I. Souza, J. Íñiguez, and D. Vanderbilt, "First-principles approach to insulators in finite electric fields," Phys. Rev. Lett. **89**, 117602 (2002).

[2] P. Umari and A. Pasquarello, "*Ab initio* molecular dynamics in a finite homogeneous electric field," Phys. Rev. Lett. **89**, 157602 (2002).

[3] R. W. Nunes and X. Gonze, "Berry-phase treatment of the homogeneous electric field perturbation in insulators," Phys. Rev. B **63**, 155107 (2001).

[4] R. Resta, "Macroscopic polarization in crystalline dielectrics: the geometric phase approach," Rev. Mod. Phys. **66**, 899–915 (1994).

[5] J. Paier, M. Marsman, and G. Kresse, "Dielectric properties and excitons for extended systems from hybrid functionals," Phys. Rev. B **78**, 121201 (2008).

[6] J. H. Skone, M. Govoni, and G. Galli, "Self-consistent hybrid functional for condensed systems," Phys. Rev. B **89**, 195112 (2014).

[7] W. Chen, G. Miceli, G.-M. Rignanese, and A. Pasquarello, "Nonempirical dielectric-dependent hybrid functional with range separation for semiconductors and insulators," Phys. Rev. Mater. **2**, 073803 (2018).

[8] R. Resta and K. Kunc, "Self-consistent theory of electronic states and dielectric response in semiconductors," Phys. Rev. B **34**, 7146–7157 (1986).

[9] M. S. Hybertsen and S. G. Louie, "*Ab initio* static dielectric matrices from the density-functional approach. I. Formulation and application to semiconductors and insulators," Phys. Rev. B **35**, 5585–5601 (1987).

[10] R. W. Nunes and D. Vanderbilt, "Real-space approach to calculation of electric polarization and dielectric constants," Phys. Rev. Lett. **73**, 712–715 (1994).

[11] P. Fernández, A. Dal Corso, and A. Baldereschi, "*Ab initio* study of the dielectric properties of silicon and gallium arsenide using polarized Wannier functions," Phys. Rev. B **58**, R7480–R7483 (1998).

[12] R. D. King-Smith and D. Vanderbilt, "Theory of polarization of crystalline solids," Phys. Rev. B **47**, 1651–1654 (1993).

[13] M. Gajdoš, K. Hummer, G. Kresse, J. Furthmüller, and F. Bechstedt, "Linear optical properties in the projector-augmented wave methodology," Phys. Rev. B **73**, 045112 (2006).

[14] Z.-H. Cui, Y.-C. Wang, M.-Y. Zhang, X. Xu, and H. Jiang, "Doubly screened hybrid functional: An accurate first-principles approach for both narrow- and wide-gap semiconductors," J. Phys. Chem. Lett. **9**, 2338–2345 (2018).

[15] D. Cahen and A. Kahn, "Electron energetics at surfaces and interfaces: Concepts and experiments," Adv. Mater. **15**, 271–277 (2003).

[16] Y. Hinuma, A. Grüneis, G. Kresse, and F. Oba, "Band alignment of semiconductors from density-functional theory and many-body perturbation theory," Phys. Rev. B **90**, 155405 (2014).

[17] Z. Guo, F. Ambrosio, W. Chen, P. Gono, and A. Pasquarello, "Alignment of redox levels at semiconductor–water interfaces," Chem. Mater. **30**, 94–111 (2017).

[18] A. Grüneis, G. Kresse, Y. Hinuma, and F. Oba, "Ionization potentials of solids: The importance of vertex corrections," Phys. Rev. Lett. **112**, 096401 (2014).

[19] Y. Hinuma, Y. Kumagai, I. Tanaka, and F. Oba, "Band alignment of semiconductors and insulators using dielectric-dependent hybrid functionals: Toward high-throughput evaluation," Phys. Rev. B **95**, 075302 (2017).

[20] Y. Hinuma, Y. Kumagai, I. Tanaka, and F. Oba, "Effects of composition, crystal structure, and surface orientation on band alignment of divalent metal oxides: A first-principles study," Phys. Rev. Mater. **2**, 124603 (2018).

[21] S. Steiner, S. Khmelevskyi, M. Marsmann, and G. Kresse, "Calculation of the magnetic anisotropy with projected-augmented-wave methodology and the case study of disordered $\mathrm{Fe}_{1-x}\mathrm{Co}_x$ alloys," Phys. Rev. B **93**, 224425 (2016).

[22] W. P. Huhn and V. Blum, "One-hundred-three compound band-structure benchmark of post-self-consistent spin-orbit coupling treatments in density functional theory," Phys. Rev. Mater. **1**, 033803 (2017).

[23] P. Borlido, T. Aull, A. W. Huran, F. Tran, M. A. L. Marques, and S. Botti, "Large-scale benchmark of exchange–correlation functionals for the determination of electronic band gaps of solids," J. Chem. Theory Comput. **15**, 5069–5079 (2019).

[24] K. Hummer, A. Grüneis, and G. Kresse, "Structural and electronic properties of lead chalcogenides from first principles," Phys. Rev. B **75**, 195211 (2007).

[25] J. N. Zemel, J. D. Jensen, and R. B. Schoolar, "Electrical and optical properties of epitaxial films of PbS, PbSe, PbTe, and SnTe," Phys. Rev. **140**, A330–A342 (1965).

[26] O. Madelung, *Semiconductors: Data Handbook* (Springer Berlin Heidelberg, 2004).

[27] B. Cunningham, M. Grüning, D. Pashov, and M. van Schilfgaarde, "$QSG\hat{W}$: quasiparticle self-consistent GW with ladder diagrams in W," Phys. Rev. B **108**, 165104 (2023).

[28] Y.-S. Kim, K. Hummer, and G. Kresse, "Accurate band structures and effective masses for InP, InAs, and InSb using hybrid functionals," Phys. Rev. B **80**, 035203 (2009).

[29] A. Miglio, V. Brousseau-Couture, E. Godbout, G. Antonius, Y.-H. Chan, S. G. Louie, M. Côté, M. Giantomassi, and X. Gonze, "Predominance of non-adiabatic effects in zero-point renormalization of the electronic band gap," npj Comput. Mater. **6**, 167 (2020).

[30] H. Shang, J. Zhao, and J. Yang, "Assessment of the mass factor for the electron–phonon coupling in solids," J. Phys. Chem. C **125**, 6479–6485 (2021).

[31] M. Zacharias and F. Giustino, “One-shot calculation of temperature-dependent optical spectra and phonon-induced band-gap renormalization,” Phys. Rev. B **94**, 075125 (2016).

[32] F. Karsai, M. Engel, E. Flage-Larsen, and G. Kresse, “Electron–phonon coupling in semiconductors within the GW approximation,” New J. Phys. **20**, 123008 (2018).

[33] J. P. Perdew and Y. Wang, “Accurate and simple analytic representation of the electron-gas correlation energy,” Phys. Rev. B **45**, 13244–13249 (1992).

[34] W. Kohn and L. J. Sham, “Self-consistent equations including exchange and correlation effects,” Phys. Rev. **140**, A1133–A1138 (1965).

[35] J. P. Perdew, A. Ruzsinszky, J. Tao, V. N. Staroverov, G. E. Scuseria, and G. I. Csonka, “Prescription for the design and selection of density functional approximations: More constraint satisfaction with fewer fits,” J. Chem. Phys. **123**, 062201 (2005).

[36] J. P. Perdew and K. Schmidt, “Jacob’s ladder of density functional approximations for the exchange-correlation energy,” AIP Conf. Proc. **577**, 1–20 (2001).

[37] J. Tao, J. P. Perdew, V. N. Staroverov, and G. E. Scuseria, “Climbing the density functional ladder: Nonempirical meta–generalized gradient approximation designed for molecules and solids,” Phys. Rev. Lett. **91**, 146401 (2003).

[38] J. Sun, A. Ruzsinszky, and J. P. Perdew, “Strongly constrained and appropriately normed semilocal density functional,” Phys. Rev. Lett. **115**, 036402 (2015).

[39] J. W. Furness, A. D. Kaplan, J. Ning, J. P. Perdew, and J. Sun, “Accurate and numerically efficient r^2SCAN meta-generalized gradient approximation,” J. Phys. Chem. Lett. **11**, 8208–8215 (2020).

[40] T. Lebeda, T. Aschebrock, and S. Kümmel, “Balancing the contributions to the gradient expansion: Accurate binding and band gaps with a nonempirical meta-GGA,” Phys. Rev. Lett. **133**, 136402 (2024).

[41] Z.-h. Yang, H. Peng, J. Sun, and J. P. Perdew, “More realistic band gaps from meta-generalized gradient approximations: Only in a generalized Kohn-Sham scheme,” Phys. Rev. B **93**, 205205 (2016).

[42] P. Borlido, J. Schmidt, A. W. Huran, F. Tran, M. A. L. Marques, and S. Botti, “Exchange-correlation functionals for band gaps of solids: benchmark, reparametrization and machine learning,” npj Comput. Mater. **6**, 96 (2020).

[43] T. Aschebrock and S. Kümmel, “Ultranonlocality and accurate band gaps from a meta-generalized gradient approximation,” Phys. Rev. Research **1**, 033082 (2019).

[44] T. Aschebrock, T. Lebeda, M. Brütting, R. Richter, I. Schelter, and S. Kümmel, “Exact exchange-like electric response from a meta-generalized gradient approximation: A semilocal realization of ultranonlocality,” J. Chem. Phys. **159**, 234107 (2023).

[45] T. Lebeda, T. Aschebrock, J. Sun, L. Leppert, and S. Kümmel, “Right band gaps for the right reason at low computational cost with a meta-GGA,” Phys. Rev. Materials **7**, 093803 (2023).

[46] A. Alkauskas, P. Broqvist, and A. Pasquarello, “Defect levels through hybrid density functionals: Insights and applications,” Phys. Status Solidi B **248**, 775–789 (2011).

[47] F. Tran, “On the accuracy of the non-self-consistent calculation of the electronic structure of solids with hybrid functionals,” Phys. Lett. A **376**, 879–882 (2012).

[48] W. Chen and A. Pasquarello, “Band-edge positions in *GW*: Effects of starting point and self-consistency,” Phys. Rev. B **90**, 165133 (2014).

[49] V. Fiorentini and A. Baldereschi, “Dielectric scaling of the self-energy scissor operator in semiconductors and insulators,” Phys. Rev. B **51**, 17196–17198 (1995).

[50] T. S. Moss, “Relations between the refractive index and energy gap of semiconductors,” Phys. Status Solidi B **131**, 415–427 (1985).

[51] J. He and C. Franchini, “Assessing the performance of self-consistent hybrid functional for band gap calculation in oxide semiconductors,” J. Phys. Condens. Matter **29**, 454004 (2017).

[52] H. Wang, R. Ouyang, W. Chen, and A. Pasquarello, “High-quality data enabling universality of band gap descriptor and discovery of photovoltaic perovskites,” J. Am. Chem. Soc. **146**, 17636–17645 (2024).

[53] M. Gerosa, C. E. Bottani, L. Caramella, G. Onida, C. Di Valentin, and G. Pacchioni, “Electronic structure and phase stability of oxide semiconductors: Performance of dielectric-dependent hybrid functional DFT, benchmarked against *GW* band structure calculations and experiments,” Phys. Rev. B **91**, 155201 (2015).

[54] M. Gerosa, C. E. Bottani, C. Di Valentin, G. Onida, and G. Pacchioni, “Accuracy of dielectric-dependent hybrid functionals in the prediction of optoelectronic properties of metal oxide semiconductors: a comprehensive comparison with many-body *GW* and experiments,” J. Phys.: Condens. Matter **30**, 044003 (2017).

[55] P. Liu, C. Franchini, M. Marsman, and G. Kresse, “Assessing model-dielectric-dependent hybrid functionals on the antiferromagnetic transition-metal monoxides MnO, FeO, CoO, and NiO,” J. Phys.: Condens. Matter **32**, 015502 (2019).

[56] J. Zhan, M. Govoni, and G. Galli, “Dielectric-dependent range-separated hybrid functional calculations for metal oxides,” Phys. Rev. Materials **9**, 053808 (2025).

[57] D. Deguchi, K. Sato, H. Kino, and T. Kotani, “Accurate energy bands calculated by the hybrid quasiparticle self-consistent *GW* method implemented in the ecalj package,” Jpn. J. Appl. Phys. **55**, 051201 (2016).

[58] M. S. Abdallah and A. Pasquarello, “Quasiparticle self-consistent *GW* with effective vertex corrections in the polarizability and the self-energy applied to MnO, FeO, CoO, and NiO,” Phys. Rev. B **110**, 155105 (2024).

[59] Y. Zhang, J. Furness, R. Zhang, Z. Wang, A. Zunger, and J. Sun, “Symmetry-breaking polymorphous descriptions for correlated materials without interelectronic U,” Phys. Rev. B **102**, 045112 (2020).

[60] Y. Wang, M. Engel, C. Lane, Y. Zhang, H. Miranda, L. Hou, B. Barbiellini, R. S. Markiewicz, J.-X. Zhu, G. Kresse, A. Bansil, J. Sun, and R. Zhang, “Accurate electron-phonon interactions from advanced density functional theory,” PRX Energy **5**, 013002 (2026).

[61] H. Zheng, M. Govoni, and G. Galli, “Dielectric-dependent hybrid functionals for heterogeneous materials,” Phys. Rev. Materials **3**, 073803 (2019).

[62] J. Zhan, M. Govoni, and G. Galli, “Nonempirical range-separated hybrid functional with spatially dependent screened exchange,” J. Chem. Theory Comput. **19**, 5851–5862 (2023).

[63] J. W. Furness, Y. Zhang, C. Lane, I. G. Buda, B. Barbiellini, R. S. Markiewicz, A. Bansil, and J. Sun, “An accurate first-principles treatment of doping-dependent electronic structure of high-temperature cuprate superconductors,” Commun. Phys. **1**, 11 (2018).

[64] H. Schulz and K. Thiemann, “Crystal structure refinement of AlN and GaN,” Solid State Commun. **23**, 815–819 (1977).

[65] L. Schimka, J. Harl, and G. Kresse, “Improved hybrid functional for solids: The HSEsol functional,” J. Chem. Phys. **134**, 024116 (2011).

[66] W. Paszkowicz, R. Černý, and S. Krukowski, “Rietveld refinement for indium nitride in the 105–295 K range,” Powder Diffr. **18**, 114–121 (2003).

[67] J. K. Burdett, T. Hughbanks, G. J. Miller, J. W. Richardson, and J. V. Smith, “Structural-electronic relationships in inorganic solids: powder neutron diffraction studies of the rutile and anatase polymorphs of titanium dioxide at 15 and 295 K,” J. Am. Chem. Soc. **109**, 3639–3646 (1987).

[68] H. Sowa, “On the mechanism of the pressure-induced wurtzite- to NaCl-type phase transition in CdS: an X-ray diffraction study,” Solid State Sci. **7**, 73–78 (2005).

[69]H. Sowa, "The high-pressure behaviour of CdSe up to 3 GPa and the orientation relations between its wurtzite- and NaCl-type modifications," Solid State Sci. **7**, 1384–1389 (2005).
[70]A. A. Bolzan, C. Fong, B. J. Kennedy, and C. J. Howard, "Structural studies of rutile-type metal dioxides," Acta Cryst. B **53**, 373–380 (1997).
[71]K. Rośsocisżewski, B. Paulus, P. Fulde, and H. Stoll, "*Ab initio* coupled-cluster calculations for the fcc and hcp structures of rare-gas solids," Phys. Rev. B **62**, 5482–5488 (2000).
[72]S. Riemelmoser, M. Kaltak, and G. Kresse, "Optimized effective potentials from the random-phase approximation: Accuracy of the quasiparticle approximation," J. Chem. Phys. **154**, 154103 (2021).
[73]Y. Kaneko and T. Koda, "New developments in IIa–VIb (alkaline-earth chalcogenide) binary semiconductors," J. Cryst. Growth **86**, 72–78 (1988).
[74]M. L. D. Franckel, S. Falletta, W. Chen, and A. Pasquarello, "Absorption lines of F centers in MgO and CaO through time-dependent hybrid-functional theory calculations," Phys. Rev. B **112**, 104101 (2025).
[75]R. Whited, C. J. Flaten, and W. Walker, "Exciton thermoreflectance of MgO and CaO," Solid State Commun. **13**, 1903–1905 (1973).
[76]J. M. Crowley, J. Tahir-Kheli, and W. A. Goddard, "Resolution of the band gap prediction problem for materials design," J. Phys. Chem. Lett. **7**, 1198–1203 (2016).
[77]J. A. Spencer, A. L. Mock, A. G. Jacobs, M. Schubert, Y. Zhang, and M. J. Tadjer, "A review of band structure and material properties of transparent conducting and semiconducting oxides: Ga_2O_3, Al_2O_3, In_2O_3, ZnO, SnO_2, CdO, NiO, CuO, and Sc_2O_3," Appl. Phys. Rev. **9**, 011315 (2022).
[78]A. Svane, N. E. Christensen, M. Cardona, A. N. Chantis, M. van Schilfgaarde, and T. Kotani, "Quasiparticle self-consistent GW calculations for PbS, PbSe, and PbTe: Band structure and pressure coefficients," Phys. Rev. B **81**, 245120 (2010).
[79]D. Wing, G. Ohad, J. B. Haber, M. R. Filip, S. E. Gant, J. B. Neaton, and L. Kronik, "Band gaps of crystalline solids from Wannier-localization–based optimal tuning of a screened range-separated hybrid functional," Proc. Natl. Acad. Sci. **118**, e2104556118 (2021).
[80]S. E. Gant, J. B. Haber, M. R. Filip, F. Sagredo, D. Wing, G. Ohad, L. Kronik, and J. B. Neaton, "Optimally tuned starting point for single-shot GW calculations of solids," Phys. Rev. Materials **6**, 053802 (2022).
[81]J. A. Van Vechten, "Quantum dielectric theory of electronegativity in covalent systems. I. Electronic dielectric constant," Phys. Rev. **182**, 891–905 (1969).
[82]M. E. Lines, "Bond-orbital theory of linear and nonlinear electronic response in ionic crystals. I. Linear response," Phys. Rev. B **41**, 3372–3382 (1990).
[83]H. Finkenrath, N. Uhle, and W. Waidelich, "The influence of phonons and polarons on the infrared absorption of cadmium oxide," Solid State Commun. **7**, 11–14 (1969).
[84]D. F. Nelson, R. C. Miller, and D. A. Kleinman, "Band nonparabolicity effects in semiconductor quantum wells," Phys. Rev. B **35**, 7770–7773 (1987).
[85]M. Laurien and O. Rubel, "Benchmarking exchange-correlation potentials with the mstar60 dataset: Importance of the nonlocal exchange potential for effective mass calculations in semiconductors," Phys. Rev. B **106**, 045204 (2022).
[86]S. Shang, A. Saengdeejing, Z. Mei, D. Kim, H. Zhang, S. Ganeshan, Y. Wang, and Z. Liu, "First-principles calculations of pure elements: Equations of state and elastic stiffness constants," Comput. Mater. Sci. **48**, 813–826 (2010).
