# Supplementary material for: Dielectric-dependent hybrid functional based on meta-GGA
Source: Nat Commun. 2026 Jul 4;17:8323. doi: 10.1038/s41467-026-75146-x (PMC13470121; doi:10.1038/s41467-026-75146-x)
Supplement: Supplementary file 2 [file 41467_2026_75146_MOESM2_ESM.pdf]

nature portfolio

Peer Review File

Dielectric-dependent hybrid functional based on meta-GGA

Corresponding Author: Dr Stefan Riemelmoser

This file contains all reviewer reports in order by version, followed by all author rebuttals in order by version.

Attachments originally included by the reviewers as part of their assessment can be found at the end of this file.

Version 0:

Reviewer comments:

Reviewer #1

(Remarks to the Author)
In this work the authors presented a comprehensive theoretical investigation of the performances of the dielectric-dependent hybrid functional based r2SCAN meta-GGA functional. While the findings presented in the manuscript are indeed interesting, the overall novelty of the work does not warrant its publication in Nature Communications in my opinion. No new methodological developments have been presented, and the fact that combining r2SCAN and the dielectric-dependent hybrid (DDH) scheme improves the prediction of band gaps of insulating systems is not surprising somehow.

Here are a few more detailed comments.

1) The title of this work contains the statement "climbing Jacob's Ladder for the Coulomb hole" that appears to be confusing. I think the link between what is presented in the paper and the "Coulomb hole" is quite weak.

2) In the beginning of the second paragraph, the authors stated that "The Hohenberg-Kohn theorems guarantee that Kohn-Sham DFT correctly predicts the electron density and all other ground state properties (provided the exact exchange-correlation functional is used)." I don't think the statement is correct. With the exact xc functional, only the ground stated total energy, density, and other ground state properties that can be directly derived from the energy and density are guaranteed to be exactly predicted. But there are other ground state properties that cannot be derived from the total energy and density, e.g. the expectation value of the total spin square, S^2.

3) The authors mentioned that based on the consideration of computational cost, the self-consistency is not attempted in their r2SCAN-DDH approach. But one-shot treatment can be problematic if the r2SCAN itself can not open a gap. Although the latter happens much more rarely, as indicated by the authors' investigation, it can still happens (e.g. for InSb). The authors did not clearly indicate how that situation was tackled in their calculations.

4) As mentioned above, the overall improved performances achieved by r2SCAN-based DD hybrid functional are not surprising for the systems considered in this work. I think it would be of greater interest to see how the approach performs for more challenging systems like strongly correlated transition metal oxides.

Reviewer #2

(Remarks to the Author)
comments attached below

Reviewer #3

(Remarks to the Author)
HSE is the current standard hybrid density functional for materials calculations with two major shortcomings: 1) applicabilities to metals, or not recommended for metals in general, 2) empiricism of the admixing parameter that works the

best for materials with small band gaps (around 1eV if I don't make mistakes). The proposed DD-r2SCANH was demonstrated to remove the empiricism with excellent results for the band gaps, much better than the previous DD-hybrid, namely DD-PBEH. The detailed analysis on the dielectric constants obtained from r2SCAN and PBE indicates that DD-r2SCANH yields the right results with the right reasons. We consider this as a big step forward in the construction of hybrid density functionals in broad, however we don't recommend publication of this manuscript in Nat. Comm in its current form unless the authors address the following points.

1) What is the applicabilities of DD-r2SCANH (or DDH in general) on metals and, more broadly, surfaces and interfaces? How does DD-r2SCANH compare to HSE in terms of computational efficiency? It might be good to discuss any extra steps needed for DD-r2SCANH in comparison with HSE, and possible problems they could cause.

2) Does DDH respect the size consistency? For example, the admixing parameter should change in principle for reactants and products in a reaction (similar to optimally tuned range-separated hybrids). Will the reaction energies calculated by a DDH be meaningful? And if DD-r2SCANH is applied to calculate reaction energies for thermodynamic stabilities of materials, how reliable is it? Also, somewhat minor, can DD-r2SCANH relax structures? We guess so, but didn't see the description in the manuscript.

3) Fig. 1 is not clear to us after reading it carefully, and we suggest the authors to elaborate it for general readers. Basically, the first subsection of the results section should be extracted out as a Theory section, in our opinion.

4) We understand that there are many properties to be tested for a newly proposed density functional, which couldn't be tested in one paper. However, since the DD-r2SCANH is poised to be a general density functional for predicting band gaps, it would be natural to test several difficult transition metal compounds, for example, 3d monoxides (MnO, CoO, NiO, and probably the most difficult FeO).

5) We guess the Coulomb hole is a term more in the many-body perturbation method side, and in DFT it is usually called correlation hole, which might be the reason we were confused by Fig.1. However, from the whole manuscript, we didn't see a clear analysis of the Coulomb hole since it doesn't have a clear definition other than the Sigma_COH or figures for it. We sort of get the idea about the effect of r2SCAN and PBE correlation functionals on the band gap predictions in the DDH framework. However, there are too many approximations that have been involved. We suggest the authors revise their title, and/or have the theory section that clearly explains the Coulomb hole and discusses the evidence there to support their title if they would like to keep it.

Below are some other points:

- When discussing about the improved dielectric constants from r2SCAN, it might be appropriate to also cite arXiv:2411.08192, where r2SCAN has been shown to improve the dielectric constants of NiO and CoO over PBE.
- In Table IV, why HSE is not included for comparison, similar to Tables II and III?
- Given that improved band gaps correspond to improved dielectric constants, would the authors expect improved dielectric constants and an improved DD-DFTH from using meta-GGAs with higher accuracy for band gaps than SCAN, e.g., TASK or LAK?
- In line 35, the authors call the „band gap problem" of DFT the most notorious example of a failure of DFT for excited state properties. We consider this statement at least misleading. First, in a finite system, the fundamental band gap can be obtained as the difference ground-state properties (although systems, respectively). Second, it is unclear to us how the fundamental gaps would be interpreted as excited state properties. This confusion is likely connected to the next point and the related confusion of fundamental and optical gaps in the literature (the latter are indeed excited state properties).
- In the second paragraph (lines 33 to 45), the authors should make more clear the different sources of errors in band gap calculations. Although this becomes clearer throughout the manuscript, this paragraph could be made more easy to follow. The sources of errors include errors from the scheme used (Kohn-Sham, OEP, or GKS) and errors from the type of xc functional used (LDA/GGA, meta-GGA, or hybrid). In particular, it does not become clear that using OEP or GKS itself does not improve the band gaps, but only if they are used in combination with a nonlocal xc functional (meta-GGA or hybrid).
- In line 41, the authors mention generalized Kohn-Sham schemes (GKS) (intentionally used in plural here to emphasize that there is no unique GKS). We recommend to cite the back-to-back reference for band gaps with GKS, [Phys. Rev. B 53, 3764 (1996)].
- In line 42, the authors mention that meta-GGAs can achieve „this" if evaluated in a GKS. We recommend to specify „this" as, e.g., „including the (ultra)nonlocality associated with the derivative discontinuity and thus more reliable band gaps". The concept of ultranonlocality is explained nicely in [J. Chem. Phys. 159, 234107 (2023)]. How meta-GGAs can include a realistic derivative discontinuity is discussed in [Phys. Rev. Res. 1, 033082 (2019)], [Phys. Rev. Mater. 7, 093803 (2023)], and [Phys. Rev. Lett. 133, 136402 (2024)].
- In line 85, the authors suggest alpha=eps_inf^-1 as an exact constraint. We agree that this is an exact condition but only for |r-r'|→inf, which should be made more clear in the text.
- In line 91, the authors write „After decades of hybrid functionals based on GGA functionals, we now proceed to meta-GGA." This statement is misleading, as it neglects the vast amount of literature on meta-GGA hybrids. We assume that the statement should refer to DD-Hybrids only. Thus, we recommend a more conservative statement that clearly points out that meta-GGA hybrids are not new but DD-Hybrids based on meta-GGAs are underexplored.
- In line 96, please make more clear that the linear interpolation of the band gap between DFT and HF is an approximation, neglecting changes in the orbitals. Although this point is taken up later in the manuscript, readability would be improved if this was made clear from the beginning. Maybe writing „[…] interpolates the band gap approximately linearly between […]" would be sufficient.
- Lines 135-138 discuss why meta-GGAs, specifically r2SCAN, can yield more accurate band gaps than GGA functionals. The authors mention the important contributions of using GKS instead of KS and reduced self-interaction error. However, they completely miss the importance of including the derivative discontinuity in meta-GGAs, see, e.g., [Phys. Rev. Mater. 7, 093803 (2023)]
- In line 156, the authors write „It is clear that the addition of Hartree-Fock exchange is required for both PBE and SCAN/r2SCAN to obtain realistic band gaps." We agree that adding HF exchange can significantly improve the band gaps

of PBE and SCAN/r2SCAN. But we would not phrase it a requirement, since various alternatives exist, such as adding a Hubbard U, using meta-GGAs (or SCAN-parameters) tailored towards band gaps, or using one-shot G0W0@DFT.
- Lines 196 and 302: Missing „H“ in DD-r2SCANH.
- Lines 197-203 compare the performance of DD-R2SCANH for band gaps with that of range-separated hybrid (RSH) functionals. In this context, a comparison with the recent RSH with spatially dependent screened exchange [J. Chem. Theory Comput. 19, 17, 5851–5862 (2023)] and optimally-tuned RSH [PNAS 118 (34) e2104556118 (2021)] would be interesting.
- Caption of Table IV: What do the authors mean by „Band gaps […] are mostly interpolated via Eq. (7)“? We understand that Eq. (7) can provide a reasonable approximation but why are the band gaps not calculated explicitly? We would expect the computational demand for calculating DD-R2SCANH as similar to HF. Please explain more.
- Lines 293-295: (i) and (iii) are not exactly variables. Maybe „factors“ would provide a more precise description.
- Eq. (8): Please explain this approximation more: Why not use E_g^HF – E_g^DFTx directly? How is this approximation affected by orbital relaxation?
- Line 317: „both“ should be removed.
- Table S1 in the Supplemental Material: The caption contains an undefined reference („??“)

Reviewer #4

(Remarks to the Author)
I co-reviewed this manuscript with one of the reviewers who provided the listed reports. This is part of the Nature Communications initiative to facilitate training in peer review and to provide appropriate recognition for Early Career Researchers who co-review manuscripts.

Version 1:

Reviewer comments:

Reviewer #1

(Remarks to the Author)
Even with the revisions that the authors have made to their manuscript, I still maintain my previous assessment that the overall novelty of the work does not warrant its publication in Nature Communication.

Reviewer #2

(Remarks to the Author)
The authors have satisfactorily addressed most of my concerns, and I recommend the manuscript for publication in its current form.

Reviewer #3

(Remarks to the Author)
This is reviewer #3, and I have went through the responses, including those to the other reviewers. I am satisfied with their responses except for the point about the Coulomb hole, which in my opinion is a flaw of this manuscript in particular for Nature Communications. I went through the new section "Theoretical background", which is very helpful for understanding the picture the authors have. I can follow all of them now. But this raises serious doubts on the theoretical foundation for using the Coulomb hole in the title.

Basically there are two different branches that the authors would like to connect: 1. The generalized Kohn-Sham DFT which has a firm foundation for the ground state energy and electron density, but their orbitals in general don't have physical meanings except for the frontier orbitals; 2. The quasiparticle energies and associated self-energy in GW, which has clear physical meanings. Equations 2-8 are all fine on their own domains, but jumping from Eq Sigma_COH of Eq(6) to Eq (9) is not very clear to me and involves too many approximations there. The most difficult and confusing thing to me is how to connect total energy expression of GKS of Eq (9) to the self-energy expression of Eq (6). Is it just a handwaving argument. If not, the authors have to break it down into steps with supporting evidences.

To be clear, I am not saying that the authors were wrong. The connection could be right and could be kept in the "Theoretical background" (although I suggest the authors to tune down the tone), but there were no clear derivation or strong arguments, and therefore I suggest the authors to remove "Climbing Jacob's Ladder for the Coulomb hole". I think "Dielectric-dependent hybrid functional based on meta-GGA" is a good title by itself for the novelty and performance.

Version 2:

Reviewer comments:

Reviewer #3

(Remarks to the Author)

This is reviewer #3.

I am satisfied with the authors's response, and recommend for publication of this manuscript.

One more thing to add for the authors to consider. SCF DDH-r2SCAN opens a band gap for InSb which is a metal from r2SCAN. Will it be possible DDH-r2SCAN spuriously opens a band gap for real metals in some rare cases? Maybe the authors can add some discussions on it.

Open Access This Peer Review File is licensed under a Creative Commons Attribution 4.0 International License, which permits use, sharing, adaptation, distribution and reproduction in any medium or format, as long as you give appropriate credit to the original author(s) and the source, provide a link to the Creative Commons license, and indicate if changes were made.
In cases where reviewers are anonymous, credit should be given to 'Anonymous Referee' and the source.
The images or other third party material in this Peer Review File are included in the article's Creative Commons license, unless indicated otherwise in a credit line to the material. If material is not included in the article's Creative Commons license and your intended use is not permitted by statutory regulation or exceeds the permitted use, you will need to obtain permission directly from the copyright holder.
To view a copy of this license, visit https://creativecommons.org/licenses/by/4.0/

Climbing Jacob's Ladder for the Coulomb hole: Dielectric-dependent hybrid functional based on meta-GGA: Detailed responses to the Reviewers' reports.

In the following point-to-point response, we address comments by the four anonymous Reviewers. Before addressing the comments in detail, we want to thank all Reviewers for the valued time and effort dedicated to our manuscript. Their feedback has been instrumental for the revision our manuscript, substantially improving upon the original version. The Reviewers' comments are given in *blue italic text,* while our responses are given in black plain text.

Reviewer #1

In this work the authors presented a comprehensive theoretical investigation of the performances of the dielectric-dependent hybrid functional based r2SCAN meta-GGA functional. While the findings presented in the manuscript are indeed interesting, the overall novelty of the work does not warrant its publication in Nature Communications in my opinion. No new methodological developments have been presented, and the fact that combining r2SCAN and the dielectric-dependent hybrid (DDH) scheme improves the prediction of band gaps of insulating systems is not surprising somehow.

We appreciate that the Reviewer finds our work interesting and that they value its comprehensiveness. However, we respectfully disagree with the Reviewer on his opinion about the novelty of our work. The Reviewer does not find surprising that DD-r^{2}SCANH improves upon DD-PBEH, and claims that this diminishes the novelty of our work. On the contrary, it is not obvious that any meta-GGA functional should necessarily yield improvements over PBE. Indeed, the newly added comparison of r^{2}SCAN to other meta-GGA base functionals, in particular LAK,[1] (Lebeda-Aschebrock-Kümmel) demonstrates that it is not sufficient for the meta-GGA base functional to yield good band gaps on its own. Rather, the meta-GGA functional should be a good approximation to the Coulomb hole [see Fig. 1 (b) in the revised manuscript], and should yield good dielectric constants [see Fig. 3 (b) in the revised manuscript]. In the words of Reviewers 3 and 4: "DD-r^{2}SCANH yields the right results with the right reasons". Furthermore, we are convinced that it is a strength of our work that this improvement is in line with physical intuition. Namely, DD-r^{2}SCANH inherits the improvements in electronic structure from the DFT base functional. Moreover, the fact that our method is simple and effective will contribute to its widespread adaptation.

Here are a few more detailed comments.

1. *The title of this work contains the statement "climbing Jacob's Ladder for the Coulomb hole" that appears to be confusing. I think the link between what is presented in the paper and the "Coulomb hole" is quite weak.*

   We have made substantial changes to the theory section, and now link the Coulomb hole with the exchange-correlation part of the remainder potential in hybrid functional GKS.[2] To the best of our knowledge, this represents a novel theoretical insight.

2. *In the beginning of the second paragraph, the authors stated that "The Hohenberg-Kohn theorems guarantee that Kohn-Sham DFT correctly predicts the electron density and all other ground state properties (provided the exact exchange-correlation functional is used)." I don't think the statement is correct. With the exact xc functional, only the ground stated total energy, density, and other ground state properties that can be directly derived from the energy and density are guaranteed to be exactly predicted. But there are other ground state properties that cannot be derived from the total energy and density, e.g. the expectation value of the total spin square, S^2.*

   Following the suggestion of the Reviewer, we have reformulated this sentence. A more precise statement is indeed (lines 33-35 in the revised main text):

   "The Hohenberg-Kohn theorems guarantee that Kohn-Sham DFT correctly predicts important ground state properties, including the electron density and the total energy, as well as other ground state properties that can be extracted from them (provided the exact exchange-correlation functional is used)."

3. *The authors mentioned that based on the consideration of computational cost, the self-consistency is not attempted in their r2SCAN-DDH approach. But one-shot treatment can be problematic if the r2SCAN itself can not open a gap. Although the latter happens much more rarely, as indicated by the authors' investigation, it can still happens (e.g. for InSb). The authors did not clearly indicate how that situation was tackled in their calculations.*

   A one-shot treatment is indeed problematic if the DFT base functional yields a false metallic state. That is, using $\alpha = 1/\varepsilon_\infty^{\mathrm{DFT}}$ vanishes, and the DD-DFTH band gap equals the band gap of its DFT base functional. Originally, we had not applied any special measures for InSb, and consequently assigned zero band gap for the one-shot

DD-r^2SCANH($\alpha = 1/\varepsilon_\infty^{r^2SCAN}$) for the statistics in Table IV. Nonetheless, we agree with the reviewer that this is an interesting point, and we now treat the case of InSb more extensively. The newly added Supplementary Note 8 shows that self-consistent DD-r^2SCANH achieves a semiconducting ground state, and the self-consistently calculated inverse dielectric constant is in good agreement with the experimental one, see also the newly added Supplementary Fig. S8. This figure also confirms that self-consistent DD-r^2SCANH yields only minor changes for a prototypical semiconductor (GaAs), where the dielectric constant obtained by r^2SCAN is already close to the experimental one.

4. *As mentioned above, the overall improved performances achieved by r2SCAN-based DD hybrid functional are not surprising for the systems considered in this work. I think it would be of greater interest to see how the approach performs for more challenging systems like strongly correlated transition metal oxides.*

   Again, we respectfully disagree with the reviewer on the fact that the good performance of DD-r^2SCANH for conventional materials is unsurprising. On the contrary, the fact that DD-r^2SCANH rivals the accuracy of gold-standard many-body-perturbation theory methods for both band gaps and ionization potentials is unexpected and significant. We re-iterate that DD-r^2SCANH is a hybrid functional which uses only a single parameter. This parameter is determined by an exact constraint and can be calculated accurately from first principles. As such, our work represents a major theoretical advancement in the development of nonempirical hybrid functionals.
   Still, we appreciate the broad interest in strongly correlated oxides. To this point, we now discuss the application DD-r^2SCANH to the strongly correlated rock salt transition metals oxides MnO, FeO, CoO, NiO in the newly added Supplementary Note 7. In summation, we establish that: (lines 283-285 in the revised Supplementary Information)

   "... r^2SCAN clearly improves upon PBE for electronic structure properties of the rock salt transition metal monoxides. However, DD-r^2SCANH is only superior to DD-PBEH if one uses the inverse dielectric constants obtained with the respective DFT base functionals, rather than the experimental ones."

   We have also added four weakly correlated oxide semiconductors (rock salt CdO, cuprite-CuO_2, rutile-TiO_2, and rutile-SnO_2) to our benchmark. The band gap MARE for DD-r^2SCANH increases from 6% to 7%, while the MARE for DD-PBEH decreases from 29% to 27%. Tables and Figures have been updated accordingly. Altogether, we added eight interesting oxide semiconductors, increasing the number of materials studied from 35 to 43.

Reviewer #2

The authors propose a dielectric-dependent hybrid (DDH) functional, termed DD-r2SCANH, in which the PBE exchange–correlation base of conventional DDH schemes is replaced by the r2SCAN meta-GGA. This modification substantially improves the description of electronic structures, particularly for narrow-gap semiconductors where PBE fails qualitatively. The resulting accuracy is comparable to that of many-body GW methods but at a significantly reduced computational cost. Notably, the proposed framework can be extended to other GGA and meta-GGA functionals, such as TPSS- or MVS-type functionals, and the authors are encouraged to comment explicitly on this generalizability.

Following the suggestion of Reviewers 2, 3 and 4, we now examine DDH based on three more meta-GGA functionals: TPSS,[3] r^2SCAN-L,[4] and LAK.[1] To complete Jacob's Ladder, we also consider the LDA.[5] We find that DD-r^2SCANH yields the most accurate band gaps, see the newly added Figure 1 (b) in the revised main text. The performance of the various meta-GGA functionals is analyzed in more detail in the newly added Supplementary Note 5.

Apart from this, I suggest the following points be addressed before the manuscript can be considered for publication in Nature Communications:

1. *Placement of the "Climbing Jacob's Ladder for the Coulomb Hole" section: This discussion could be more appropriately placed before the Results section, for instance, under a new subsection titled Theoretical Background or similar.*

   We have created a "Theoretical background" section as suggested by the Reviewer. We have also extended the theoretical discussion by linking the Coulomb hole with the exchange-correlation part of the remainder potential in hybrid functional GKS.[2]

2. *Transition-metal oxides and Cu-chalcopyrites: While the manuscript comments on the performance of r2SCAN for transition-metal oxides and Cu-chalcopyrites, no corresponding results are presented. It would strengthen*

the paper to include at least one representative case, such as FeO in the AFM-II phase or Cu3(Sb/As)Se4, to illustrate the functional's performance for correlated systems.

We have now added eight metal-oxide systems including the strongly correlated FeO suggested by the Reviewer. For FeO in particular, we confirm the results of Ref. 6 who showed that PBE yields a false metallic ground state, while r^{2}SCAN can open a band gap, see the newly added Supplementary Note 7. The FeO band gap obtained by DD-r^{2}SCANH is 1.6 eV, falling in the middle of a rather large span of experimental references (1.1-2.2 eV, see Table S10 in the revised Supplementary Information). The magnetic moment obtained by DD-r^{2}SCANH (3.56 Bohr/atom) is in good agreement with reference results from many-body perturbation theory (3.57-3.66 Bohr/atom) (see Table S11 in the revised Supplementary Information).

3. *Behavior of ε_∞^{-1} in Fig. 3(b): In Fig. 3(b), one value of ε_∞^{-1} appears to approach zero for r2SCAN. The authors should discuss the physical reason behind this trend and its implications.*

   Indeed, r^{2}SCAN falsely predicts $\varepsilon_\infty^{-1} = 0$ for one material in Fig. 3(b), namely, InSb. This follows from the vanishing r^{2}SCAN band gap, see for instance Table I in the revised manuscript. We now show that self-consistency in the mixing parameter can solve this problem. The origins and implications are discussed in detail in the newly added Supplementary Note 8.

4. *Use of experimental dielectric constants: All band-gap calculations are performed using experimental ε_∞^{-1} . The authors should comment on what would happen if ε_∞^{-1} were computed self-consistently within DD-r2SCANH. Even if the fxc kernel contribution is neglected, this point deserves discussion.*

   Next to experimental dielectric constants, we also tested dielectric constants at the DFT level (Table IV in the main text). For two select materials (GaAs and InSb), we have now investigated a fully self-consistent determination of ε_∞^{-1} within DD-PBEH and DD-r^{2}SCANH. Supplementary Fig. S8 shows that self-consistency yields minor improvements for a prototypical semiconductor (GaAs). For InSb, both the PBE and r^{2}SCAN base functionals yield a false metallic ground state. Self-consistency correctly achieves a semiconducting ground state for DD-r^{2}SCANH, while DD-PBEH still yields a false metallic ground state. This can be understood as a consequence of the large negative PBE band gap for InSb, as discussed in detail in the newly added Supplementary Note 8. We note that InSb is the only problematic material for DD-r^{2}SCAN in this regard, see Table I in the revised main text.

5. *Ionization potentials and self-consistency: Have the authors attempted to compute ionization potentials fully self-consistently, including the evaluation of ε_∞^{-1} within DD-r2SCANH? Clarifying this would improve the methodological transparency.*

   As described in Supplementary Note 2, we used HSE lign-ups from Grüneis *et al.*, which enables a direct comparison with their GWΓ calculations. Calculating line-up potentials consistently with DDH is in principle a good idea. However, we have discussed that the differences compared to HSE lineups should be negligible for the specific materials considered here, see Supplementary Note 2. That is, even the use of PBE line-ups changes the ionization potentials by only 0.08 eV on average. The difference between PBE and HSE line-ups is significant only for three oxide materials (ZnO, MgO, CdO), where HSE can correct well-understood failures of the PBE electronic structure (see Supplementary Note 2). Concerning the role of the α parameters, we also note that the superior performance of DD-r^{2}SCANH over DD-PBEH or ionization potentials is increased, if the respective DFT inverse dielectric constants are used for the DDH functional instead of the experimental ones. This follows from the fact the PBE underestimates ε_∞^{-1} as well as the IPs, analogously to the band gap results shown in Table IV in the main text.

6. *Systems that remain metallic under r2SCAN: In Table IV, results based on ε_∞^{-1} obtained from r2SCAN are shown. How are systems treated where r2SCAN incorrectly predicts metallicity? The authors should discuss the stability and applicability of the DDH correction in such cases.*

   The newly added Supplementary Fig. S8 shows that self-consistent DD-r^{2}SCANH manages to solve the problem of false metallicity, while DD-PBEH cannot. See also the response to point 2.4.

7. *Relevance of recently proposed meta-GGAs: The authors may wish to comment on the potential use of recently developed meta-GGA functionals, such as LAK or r2SCAN-L, within the DDH framework.*

   We have now investigated the use of other meta-GGA functionals including LAK and r^{2}SCAN-L, see newly added Fig. 1 (b) in the main text, and newly added Supplementary Note S5. r^{2}SCAN-L performs slightly worse than r^{2}SCAN for both DFT and DDH band gaps. This can be attributed to the missing meta-GGA derivative discontinuity in r^{2}SCAN-L, see also Refs. 7–10. LAK yields better band gaps than r^{2}SCAN as a DFT functional, but the DD-LAKH band gaps barely improve upon LAK and are only as accurate as DD-PBEH.

In Supplementary Note S5, we discuss that this is due to an overestimation for the band gaps of narrow-gap semiconductors by DD-LAKH.

8. *Spin–orbit coupling (SOC) and zero-point renormalization (ZPR): In Table S2, it is unclear whether the error statistics include both SOC and ZPR corrections. If SOC effects are not included, the authors are encouraged to report SOC-corrected band gaps explicitly in each column.*

   The error statistics do include both SOC and ZPR correction, this has been clarified in the caption of Supplementary Table S2.

9. *Overestimation of band gaps: In some cases, DD-r2SCANH appears to overestimate the band gaps relative to experiment. The authors should highlight and discuss these deviations separately, as they may indicate limitations of the dielectric screening model or the underlying functional.*

   Indeed, we record the overestimation of band gaps for certain materials. First, a small outlier is InP, where DD-r^{2}SCANH also slightly overestimates the band gap by $\approx 10\%$ (the numerical value is 1.57 eV including ZPR and SOC, compared to 1.42 eV for expt.). However, the result is close to that of GWΓ (1.52 eV), which we find satisfactory. More systematically, the overestimation is visible for wide band-gap insulators such as Ne and NaF. In both cases, DD-PBEH and DD-r^{2}SCANH overestimate the experimental band gap by $\approx 8\%$. We also observe that DD-r^{2}SCANH overestimates the band gaps of certain oxides, which has become clearer after added eight additional oxide semiconductors to our list of materials (see also the response to point 1.4). We now show that DD-r^{2}SCANH considerably overestimates the band gap of CdO ($\approx 25\%$, see Supplementary Table S2) and NiO (≈ 15 %, see Supplementary Table S10).
   We agree with the reviewer that the observed band gap overestimations could be interpreted as a failure of the homogeneous dielectric screening model. To this point, Zhan, Govoni, and Galli[11] have shown that the spuriously overestimated DD-RSH-CAM band gap of NiO improves if one considers local variations of the dielectric constant. We now discuss the possibility of generalizing DD-r^{2}SCANH to strongly inhomogeneous materials such as NiO: (lines 264-269 in the revised Supplementary Information)

   "Moreover, the large overestimation for NiO might indicate a potential route of improvement for DD-r^{2}SCANH. Namely, Zhan, Govoni, and Galli[11] have shown that a range-separated DDH functional (DD-RSH-CAM) yields a band gap of 5.15 eV, close to our DD-r^{2}SCANH result (5.07 eV). They further showed that the band gap is reduced by 0.46 eV if local variations of the dielectric constant (and of the range-separation parameter) are considered.[12,13]"

Overall, the proposed DD-r2SCANH functional represents a valuable step forward in the development of nonempirical, dielectric-dependent hybrid functionals. Addressing the points raised above will substantially improve the manuscript's completeness and impact.

We thank the Reviewer again for relevant feedback on some key issues, and for the encouragement expressed for our work.

Reviewers #3 and #4 (co-review)

HSE is the current standard hybrid density functional for materials calculations with two major shortcomings: 1) applicabilities to metals, or not recommended for metals in general, 2) empiricism of the admixing parameter that works the best for materials with small band gaps (around 1 eV if I don't make mistakes). The proposed DD-r2SCANH was demonstrated to remove the empiricism with excellent results for the band gaps, much better than the previous DD-hybrid, namely DD-PBEH. The detailed analysis on the dielectric constants obtained from r2SCAN and PBE indicates that DD-r2SCANH yields the right results with the right reasons. We consider this as a big step forward in the construction of hybrid density functionals in broad, however we don't recommend publication of this manuscript in Nat. Comm in its current form unless the authors address the following points.

1. *What is the applicabilities of DD-r2SCANH (or DDH in general) on metals and, more broadly, surfaces and interfaces? How does DD-r2SCANH compare to HSE in terms of computational efficiency? It might be good to discuss any extra steps needed for DD-r2SCANH in comparison with HSE, and possible problems they could cause.*

   For metals, any DD-DFTH functional reduces to its DFT base functional, as the mixing parameter $\alpha = \varepsilon_\infty^{-1}$ vanishes. This is by design, as DFT is supposed to provide accurate electronic structure for (real) metals. As discussed throughout the current work, problems do certainly arise if the DFT base functional predicts a false metallic state. Regarding real metals, we find it encouraging that r^{2}SCAN can even improve upon PBE for

thermophysical properties of metals.[14] If one is interested in including exact-exchange for metals, this can be done via range-separation as for instance in DD-RSH-CAM.[15]
Surfaces and interfaces pose a conceptual challenge for DDH, since the long-range asymptotic constraint is direction dependent. More generally, this problem occurs for any strongly heterogeneous system. Galli and coworkers have proposed an extension of DDH by defining a local dielectric constant $\varepsilon_\infty(r)$,[11–13] this is now mentioned at several places in the revised manuscript (lines 62, 154 in the main text, lines 264-269 in the revised Supplementary Information). For the specific case of semiconductor interfaces, a different pragmatic approach exists.[16,17] Namely, one just takes the average screening parameter $[\alpha_{\mathrm{AB}} = (\alpha_{\mathrm{A}} + \alpha_{\mathrm{B}})/2]$, where A and B are the constituents of the interface. Experience shows that this "mixed" approach yields remarkably accurate band edge offsets, especially if α_{A} and α_{B} are similar.[16,17] We note that DD-r^{2}SCANH has a possible advantage here due to the smaller band gap slopes compared to DD-PBEH. Therefore, taking the average $\alpha_{\mathrm{AB}} = (\alpha_{\mathrm{A}} + \alpha_{\mathrm{B}})/2$ will be a better approximation for DD-r^{2}SCANH. This advantage is now mentioned in the discussion: (lines 376-378 in the revised main text)

"This is also useful for the modeling of semiconductor interfaces, where the optimal mixing parameter can be approximated by averaging the inverse dielectric constants of the two constituent semiconductors.[17]"

Regarding computational efficiency, we have argued that DD-r^{2}SCANH has essentially the same computational cost as PBE0 for bulk solids as considered in this work. HSE can provide additional speedup with respect to PBE0, as the range-separation can improve **k**-point convergence. We note however, that this comes at the cost of introducing an empirical parameter, and furthermore violates the asymptotic long-range constraint. We now mention the possibility of extending DD-r^{2}SCANH to range-seperation as in HSE as a possible pragmatic way to speedup DD-r^{2}SCANH calculations (lines 351-354 in the revised main text):

"To further reduce the computational cost of DD-r^{2}SCANH, our method could be extended towards range-separation as in HSE.[18,19] However, we do not pursue this approach here, since the range-separation comes at the cost of introducing an additional hybrid functional parameter and also violates the asymptotically exact constraint $\alpha = \varepsilon_\infty^{-1}$ for the long-range interaction."

2. *Does DDH respect the size consistency? For example, the admixing parameter should change in principle for reactants and products in a reaction (similar to optimally tuned range-separated hybrids). Will the reaction energies calculated by a DDH be meaningful? And if DD-r2SCANH is applied to calculate reaction energies for thermodynamic stabilities of materials, how reliable is it? Also, somewhat minor, can DD-r2SCANH relax structures? We guess so, but didn't see the description in the manuscript.*

DDH and, more generally, optimally tuned hybrid functionals are not size-consistent, see Refs. 20 and 21. The application of DDH to reaction energies has been very limited. Gerosa *et al.*[22] have applied DDH to calculate the structural phase energy differences for various polymorphic oxide semiconductors. They clearly demonstrate that DDH - with $\alpha = \varepsilon_\infty^{-1}$ calculated for each phase individually - can yield catastrophic results. This was demonstrated for WO_3, where high- and low-temperature phases have very different dielectric constants. In our opinion, this is in fact related to the size-consistency issue, and we do not think that DDH should be applied in this way. Rather, one has to set a single α parameter (for instance determined by the mixed scheme discussed in point 3.1). It is worth noting, however, that the applicability of DDH total energies goes beyond pristine bulk materials. For instance, DDH has been successfully used to study point defects,[17] where the long-range asymptotic constraint $\alpha = {\varepsilon_\infty}^{-1}$ remains physically applicable.
We confirm that DD-r^{2}SCANH can be used to relax structures, the standard VASP routines fully support this as of version vasp.6.4 .

To inform potential users, the following statement has been added to the Methods section (lines 421-425 in the revised main text):

"DD-r^{2}SCANH is available as of version `vasp.6.4`, including structure relaxation. Since DDH functionals and other optimally-tuned hybrid functionals are not size consistent,[20,21] comparing total energies for materials with different hybrid functional parameters is problematic. We therefore advice to select a single parameter $\alpha = \varepsilon_\infty^{-1}$ for a given material, and keep it fixed. For the study of semiconductor interfaces, we recommend the mixed scheme described in Ref. 17."

3. *Fig. 1 is not clear to us after reading it carefully, and we suggest the authors to elaborate it for general readers. Basically, the first subsection of the results section should be extracted out as a Theory section, in our opinion.*

We have created a theory section as suggested, and have added a comparison with the generalized Kohn-Sham formalism of Garrick *et al.*[2] to make this point more clear to the general reader. In essence, the Coulomb hole correspond to the exchange-correlation part of the remainder for a DDH.

4. *We understand that there are many properties to be tested for a newly proposed density functional, which couldn't be tested in one paper. However, since the DD-r2SCANH is poised to be a general density functional for predicting band gaps, it would be natural to test several difficult transition metal compounds, for example, 3d monoxides (MnO, CoO, NiO, and probably the most difficult FeO).*

   We have added eight metal-oxide systems including the four materials mentioned here, see also the response to point 1.4. The results for MnO, CoO, NiO, and FeO are discussed separately in the newly added Supplementary Note 7. In summation, we establish that: (lines 283-285 in the revised Supplementary Information)

   "... r^2SCAN clearly improves upon PBE for electronic structure properties of the rock salt transition metal monoxides. However, DD-r^2SCANH is only superior to DD-PBEH if one uses the inverse dielectric constants obtained with the respective DFT base functionals, rather than the experimental ones."

5. *We guess the Coulomb hole is a term more in the many-body perturbation method side, and in DFT it is usually called correlation hole, which might be the reason we were confused by Fig.1. However, from the whole manuscript, we didn't see a clear analysis of the Coulomb hole since it doesn't have a clear definition other than the Sigma_COH or figures for it. We sort of get the idea about the effect of r2SCAN and PBE correlation functionals on the band gap predictions in the DDH framework. However, there are too many approximations that have been involved. We suggest the authors revise their title, and/or have the theory section that clearly explains the Coulomb hole and discusses the evidence there to support their title if they would like to keep it.*

   We have reworked the theory section, and agree with the Reviewers that our definition and the physical interpretation had been too brief for general readers. We now link the Coulomb hole with the exchange-correlation part of the remainder potential in hybrid functional GKS.[2] We hope that our Coulomb hole picture now evokes the correct physical intuition, notwithstanding the approximations made.

   Below are some other points:
   - When discussing about the improved dielectric constants from r2SCAN, it might be appropriate to also cite arXiv:2411.08192, where r2SCAN has been shown to improve the dielectric constants of NiO and CoO over PBE.

   We now cite this study from Wang *et al.* in the revised main text (lines 222), as well as in the newly added Supplementary Note 7. We note that the article has meanwhile been published in PRX Energy.[23]

   - In Table IV, why HSE is not included for comparison, similar to Tables II and III?

   We had not included HSE in this table, since it is not a DDH, and the HSE statistics had been given in the Supplementary Information. On reflection, we agree with the Reviewers that HSE is important as comparison, and the numbers have been provided here as well for the ease of reference.

   - Given that improved band gaps correspond to improved dielectric constants, would the authors expect improved dielectric constants and an improved DD-DFTH from using meta-GGAs with higher accuracy for band gaps than SCAN, e.g., TASK or LAK?

   We have now investigated the use of other meta-GGA functionals including LAK, see newly added Fig. 1 (b) in the main text, and newly added Supplementary Note S5. LAK yields better band gaps than r^2SCAN as a DFT functional, but the DD-LAKH band gaps barely improves upon LAK and are only as accurate as DD-PBEH. In Supplementary Note S5, we discuss that this is due to an overestimation for the band gaps of semiconductors and especially narrow-gap semiconductors by DD-LAKH. We note that the DDH from Jana *et al.*[24] faced the same problem, and we expect that a DDH based on TASK would also overestimate band gaps.

   - In line 35, the authors call the "band gap problem" of DFT the most notorious example of a failure of DFT for excited state properties. We consider this statement at least misleading. First, in a finite system, the fundamental band gap can be obtained as the difference ground-state properties (although systems, respectively). Second, it is unclear to us how the fundamental gaps would be interpreted as excited state properties. This confusion is likely connected to the next point and the related confusion of fundamental and optical gaps in the literature (the latter are indeed excited state properties).

   Indeed, band gaps can also be obtained via total energies, although this is not pursued in this work. We further note that the fundamental gap can be understood as a single-particle excitation, while optical gaps represent two-particle excitations, see for instance Ref. 21. To avoid ambiguities, we have rephrased this passage as follows (lines 35-39 in the revised main text):

"Unfortunately, this guarantee does not apply to all electronic structure properties, and the "band gap problem" of DFT is the most notorious example.[25] In *GW* theory,[26–28] the fundamental gap can be calculated as the differences of quasi-particle excitation energies, $E_\mathrm{g} = \mathrm{CBM} - \mathrm{VBM}$. However, if the band gap is calculated in this way using the Kohn-Sham eigenvalues, PBE and other semilocal functionals like LDA drastically underestimate experimental band gaps. "

- In the second paragraph (lines 33 to 45), the authors should make more clear the different sources of errors in band gap calculations. Although this becomes clearer throughout the manuscript, this paragraph could be made more easy to follow. The sources of errors include errors from the scheme used (Kohn-Sham, OEP, or GKS) and errors from the type of xc functional used (LDA/GGA, meta-GGA, or hybrid). In particular, it does not become clear that using OEP or GKS itself does not improve the band gaps, but only if they are used in combination with a nonlocal xc functional (meta-GGA or hybrid).

Indeed, both the GKS scheme and a better xc functional are crucial to achieve better band gaps. To make this issue very clear to the general reader, we have reformulated this paragraph as follows (lines 39-52 in the revised main text):

"This failure has two components: (i) the use of the approximate Kohn-Sham DFT functional instead of the exact one, and (ii) the missing derivative discontinuity, associated with the use of a local exchange-correlation potential in Kohn-Sham DFT.[29,30] To address point (i), more accurate local exchange-correlation potentials can be constructed either via Kohn-Sham inversion[31,32] or through the optimized effective potential method.[7,33] However, the band gaps obtained with these methods barely improve upon PBE. It is therefore crucial to address point (ii), the derivative discontinuity.
In generalized Kohn-Sham schemes (GKS), one incorporates the derivative discontinuity by adopting nonlocal exchange-correlation potentials.[34–36] Meta-GGA functionals achieve this nonlocality via dependence on the kinetic energy density.[1,8–10] The band gaps obtained by SCAN and r^{2}SCAN systematically improve upon PBE band gaps, but remain underestimated with respect to experiment.[7,37–39] Hartree-Fock theory is also a GKS scheme, but drastically overestimates band gaps due to the lack of nonlocal correlation effects. Overall, this means that just the GKS scheme alone is not sufficient, and it is crucial to address both points (i) and (ii) simultaneously to achieve realistic band gaps.[21,35,40]"

- In line 41, the authors mention generalized Kohn-Sham schemes (GKS) (intentionally used in plural here to emphasize that there is no unique GKS). We recommend to cite the back-to-back reference for band gaps with GKS, [Phys. Rev. B 53, 3764 (1996)].

The work from Seidl *et al.*[35] is indeed a classic reference, and we now cite it in the Introduction and the Theory section. We also adapted the plural for GKS as suggested by the Reviewers.

- In line 42, the authors mention that meta-GGAs can achieve "this" if evaluated in a GKS. We recommend to specify "this" as, e.g., "including the (ultra)nonlocality associated with the derivative discontinuity and thus more reliable band gaps". The concept of ultranonlocality is explained nicely in [J. Chem. Phys. 159, 234107 (2023)]. How meta-GGAs can include a realistic derivative discontinuity is discussed in [Phys. Rev. Res. 1, 033082 (2019)], [Phys. Rev. Mater. 7, 093803 (2023)], and [Phys. Rev. Lett. 133, 136402 (2024)].

In line with the Reviewers' suggestion, we have rephrased this passage as follows, citing also the suggested references: (lines 46-48 in the revised main text)

"In generalized Kohn-Sham schemes (GKS), one incorporates the derivative discontinuity by adopting nonlocal exchange-correlation potentials.[34–36] Meta-GGA functionals achieve this nonlocality via dependence on the kinetic energy density.[1,8–10]"

Apart from this paragraph, we further reference these works in the newly added Supplementary Note 5.

- In line 85, the authors suggest $\alpha = \varepsilon_\infty^{-1}$ as an exact constraint. We agree that this is an exact condition but only for $|r - r'| \to \infty$, which should be made more clear in the text.

In the preceding sentence, we discuss that this constraint applies in the limit $|r - r'| \to \infty$. To be very clear about this point, we further specify: "asymptotically exact constraint".

- In line 91, the authors write "After decades of hybrid functionals based on GGA functionals, we now proceed to meta-GGA." This statement is misleading, as it neglects the vast amount of literature on meta-GGA hybrids. We assume that the statement should refer to DD-Hybrids only. Thus, we recommend a more conservative statement that clearly points out that meta-GGA hybrids are not new but DD-Hybrids based on meta-GGAs are underexplored.

Indeed, meta-GGA hybrid functionals have been used for a long time, we credit Becke[43] for his innovation in the introduction. We agree with the reviewer that a more conservative formulation is appropriate, and have revised the paragraph accordingly: (lines 130-134 in the revised main text)

"Historically, the first hybrid functional proposed by Becke was based on the LDA.[41] Becke quickly proceeded to develop hybrid functionals based on GGA and meta-GGA.[42,43] Likewise, early DDH functionals used the LDA for their DFT part,[44] and GGA was rapidly adopted into DDH functionals as well.[17] However, further improvements have remained largely unexplored. Now, we proceed with a DDH functional based on meta-GGA ..."

- In line 96, please make more clear that the linear interpolation of the band gap between DFT and HF is an approximation, neglecting changes in the orbitals. Although this point is taken up later in the manuscript, readability would be improved if this was made clear from the beginning. Maybe writing "[. . .] interpolates the band gap approximately linearly between [. . .]" would be sufficient.

We have clarified this point using the formulation "approximately linearly" as suggested.

-In line 156, the authors write "It is clear that the addition of Hartree-Fock exchange is required for both PBE and SCAN/r2SCAN to obtain realistic band gaps." We agree that adding HF exchange can significantly improve the band gaps of PBE and SCAN/r2SCAN. But we would not phrase it a requirement, since various alternatives exist, such as adding a Hubbard U, using meta-GGAs (or SCAN-parameters) tailored towards band gaps, or using one-shot G0W0@DFT.

We agree with the Reviewers that this statement was too strong. Indeed, any of the methods mentioned can also increase band gaps. We have reformulated this sentence as follows (lines 208-209 in the revised main text):

"As we demonstrate below, the addition of Fock exchange enables both PBE and r^{2}SCAN to obtain realistic band gaps."

- Lines 196 and 302: Missing "H" in DD-r2SCANH.

We have corrected this typographical error.

- Lines 197-203 compare the performance of DD-R2SCANH for band gaps with that of range-separated hybrid (RSH) functionals. In this context, a comparison with the recent RSH with spatially dependent screened exchange [J. Chem. Theory Comput. 19, 17, 5851–5862 (2023)] and optimally-tuned RSH [PNAS 118 (34) e2104556118 (2021)] would be interesting.

We added a comparison to the optimally-tuned RSH [PNAS 118 (34) e2104556118 (2021)], showing similar performance of DD-r^{2}SCANH and WOT-RSH. We note that WOT-SRH has a conceptual problem in cases where the amount of short-rang Fock exchange $\alpha^{\mathrm{SR}} = 1/4$ is numerically similar to the inverse dielectric constant. The following comment was added to the discussion (lines 256-260 in the revised main text):

"Recently, accurate band gaps were also obtained with the WOT-SRSH range-separated hybrid functional from Wing *et al.*[45,46]. WOT-SRSH yields a of MARE 7% for a subset of 15 materials, compared to 9% MARE achieved by DD-r^{2}SCANH for this subset. However, we note that the optimal tuning procedure for WOT-SRSH is fairly involved, and the range-separation parameter γ is ill-defined for materials such as AlN that have $\varepsilon_\infty^{-1} \approx 1/4$ ("γ collapse problem")."

The SE-RSH functional [J. Chem. Theory Comput. 19, 17, 5851–5862 (2023)] has not been tested extensively for bulk solids (only 9 materials were studied in this reference). However, we have already discussed the DSH and DD-RSH-CAM functionals, which are very similar to SE-RSH for bulk materials (this is in fact a construction principle of SE-RSH). We added the following comment to the discussion (lines 253-255 in the revised main text):

"Moreover, the range-separated hybrid of Zhan, Govoni, and Galli[11,13] is essentially equivalent to DD-RSH-CAM for conventional semiconductors and insulators as considered here (see however Supplementary Note 7 for a discussion of the strongly correlated material NiO)."

- Caption of Table IV: What do the authors mean by "Band gaps [. . .] are mostly interpolated via Eq. (7)"? We understand that Eq. (7) can provide a reasonable approximation but why are the band gaps not calculated explicitly? We would expect the computational demand for calculating DD-R2SCANH as similar to HF. Please explain more.

As shown recently by Yang, Falletta, and Pasquarello,[47] the hybrid functional band gaps are extremely linear as a function of mixing parameter. This is also seen in Fig. 2 (a) for the example of GaAs. We have checked that the interpolation is accurate up to 0.05 eV or better for all four interpolated band gaps for GaAs (PBE0, r^2SCAN0, and the two DD-DFTH functionals with DFT dielectric constants). However, we have treated all narrow-gap semiconductors explicitly, as here deviations from linear behavior can occur. As such, the trends and average statistics should not be significantly affected by the interpolation.

- Lines 293-295: (i) and (iii) are not exactly variables. Maybe "factors" would provide a more precise description.

We have rephrased this as “factors” following the reviewer’s suggestion.

- Eq. (8): Please explain this approximation more: Why not use $E_g^{HF}-E_g^{DFTx}$ directly? How is this approximation affected by orbital relaxation?

Apart from orbital relaxation, the quantities $E_g^{HF}-E_g^{DFTx}$ is indeed equivalent to the quantities $E_g^{HF+DFTc}-E_g^{DFT}$ given in this equation. If orbital relaxation actually matters and these expressions differ, we prefer $E_g^{HF+DFTc}-E_g^{DFT}$, since it corresponds to the actual hybrid functional band gap slopes shown Fig. 2 in the main text.

- Line 317: "both" should be removed.
- Table S1 in the Supplemental Material: The caption contains an undefined reference ("??")

These errors have been corrected. We take this opportunity to sincerely thank the Reviewers again for a very careful reading of our manuscript, and for many helpful suggestions.

Other changes

1. We have noticed that Supplementary Fig. S2 erroneously depicted the band gaps of r^2SCAN0 instead of the DFT functional made up of r^2SCAN exchange and PBE correlation. This has been corrected. The overall conclusion of nonlocal correlation reducing the band gaps is still valid. We have rephrased the sentence in the discussion referring to this Figure as follows (lines 383-384 in the revised main text):
“Supplementary Fig. S2 shows that the more nonlocal correlation functional in r^2SCAN compared to PBE generally reduces the band gaps, counteracting the effect of the more nonlocal exchange functional.”

2. We have noticed that the reference MAREs from Ref. 37 stated for the band gaps obtained by PBE and SCAN pertained erroneously to a subset of the materials studied in Ref. 37, rather than to their entire benchmark. This has been corrected. (lines 207-208 in the revised main text).

3. We have also corrected a few minor formatting and language errors throughout the manuscript and its Supplementary Information.

REFERENCES

[1] T. Lebeda, T. Aschebrock, and S. Kümmel, “Balancing the contributions to the gradient expansion: Accurate binding and band gaps with a nonempirical meta-GGA,” Phys. Rev. Lett. **133**, 136402 (2024).

[2] R. Garrick, A. Natan, T. Gould, and L. Kronik, “Exact generalized Kohn-Sham theory for hybrid functionals,” Phys. Rev. X **10**, 021040 (2020).

[3] J. Tao, J. P. Perdew, V. N. Staroverov, and G. E. Scuseria, “Climbing the density functional ladder: Nonempirical meta–generalized gradient approximation designed for molecules and solids,” Phys. Rev. Lett. **91**, 146401 (2003).

[4] D. Mejia-Rodriguez, A. A. Kunitsa, J. L. Fulton, E. Aprà, and N. Govind, “G0W0 ionization potentials of first-row transition metal aqua ions,” J. Phys. Chem. A **127**, 9684–9694 (2023).

[5] J. P. Perdew and Y. Wang, “Accurate and simple analytic representation of the electron-gas correlation energy,” Phys. Rev. B **45**, 13244–13249 (1992).

[6] Y. Zhang, J. Furness, R. Zhang, Z. Wang, A. Zunger, and J. Sun, “Symmetry-breaking polymorphous descriptions for correlated materials without interelectronic U,” Phys. Rev. B **102**, 045112 (2020).

[7] Z.-h. Yang, H. Peng, J. Sun, and J. P. Perdew, “More realistic band gaps from meta-generalized gradient approximations: Only in a generalized Kohn-Sham scheme,” Phys. Rev. B **93**, 205205 (2016).

[8] T. Aschebrock and S. Kümmel, “Ultranonlocality and accurate band gaps from a meta-generalized gradient approximation,” Phys. Rev. Research **1**, 033082 (2019).

[9] T. Aschebrock, T. Lebeda, M. Brütting, R. Richter, I. Schelter, and S. Kümmel, “Exact exchange-like electric response from a meta-generalized gradient approximation: A semilocal realization of ultranonlocality,” J. Chem. Phys. **159**, 234107 (2023).

[10]T. Lebeda, T. Aschebrock, J. Sun, L. Leppert, and S. Kümmel, "Right band gaps for the right reason at low computational cost with a meta-GGA," Phys. Rev. Materials **7**, 093803 (2023).
[11]J. Zhan, M. Govoni, and G. Galli, "Dielectric-dependent range-separated hybrid functional calculations for metal oxides," Phys. Rev. Materials **9**, 053808 (2025).
[12]H. Zheng, M. Govoni, and G. Galli, "Dielectric-dependent hybrid functionals for heterogeneous materials," Phys. Rev. Materials **3**, 073803 (2019).
[13]J. Zhan, M. Govoni, and G. Galli, "Nonempirical range-separated hybrid functional with spatially dependent screened exchange," J. Chem. Theory Comput. **19**, 5851–5862 (2023).
[14]H. Liu, X. Bai, J. Ning, Y. Hou, Z. Song, A. Ramasamy, R. Zhang, Y. Li, J. Sun, and B. Xiao, "Assessing r^{2}SCAN meta-GGA functional for structural parameters, cohesive energy, mechanical modulus, and thermophysical properties of 3d, 4d, and 5d transition metals," J. Chem. Phys. **160** (2024), 10.1063/5.0176415.
[15]W. Chen, G. Miceli, G.-M. Rignanese, and A. Pasquarello, "Nonempirical dielectric-dependent hybrid functional with range separation for semiconductors and insulators," Phys. Rev. Mater. **2**, 073803 (2018).
[16]A. Alkauskas, P. Broqvist, F. Devynck, and A. Pasquarello, "Band offsets at semiconductor-oxide interfaces from hybrid density-functional calculations," Phys. Rev. Lett. **101**, 106802 (2008).
[17]A. Alkauskas, P. Broqvist, and A. Pasquarello, "Defect levels through hybrid density functionals: Insights and applications," Phys. Status Solidi B **248**, 775–789 (2011).
[18]J. Heyd, G. E. Scuseria, and M. Ernzerhof, "Hybrid functionals based on a screened Coulomb potential," J. Chem. Phys. **118**, 8207–8215 (2003); "Erratum: "Hybrid functionals based on a screened coulomb potential" [J. Chem. Phys. 118, 8207 (2003)]," J. Chem. Phys. **124**, 219906 (2006).
[19]J. Paier, M. Marsman, K. Hummer, G. Kresse, I. C. Gerber, and J. G. Ángyán, "Screened hybrid density functionals applied to solids," J. Chem. Phys. **124**, 154709 (2006); "Erratum: "Screened hybrid density functionals applied to solids" [j. chem. phys. 124, 154709 (2006)]," J. Chem. Phys. **125**, 249901 (2006).
[20]J. H. Skone, M. Govoni, and G. Galli, "Nonempirical range-separated hybrid functionals for solids and molecules," Phys. Rev. B **93**, 235106 (2016).
[21]L. Kronik, T. Stein, S. Refaely-Abramson, and R. Baer, "Excitation gaps of finite-sized systems from optimally tuned range-separated hybrid functionals," J. Chem. Theory Comput. **8**, 1515–1531 (2012).
[22]M. Gerosa, C. E. Bottani, L. Caramella, G. Onida, C. Di Valentin, and G. Pacchioni, "Electronic structure and phase stability of oxide semiconductors: Performance of dielectric-dependent hybrid functional DFT, benchmarked against *GW* band structure calculations and experiments," Phys. Rev. B **91**, 155201 (2015).
[23]Y. Wang, M. Engel, C. Lane, Y. Zhang, H. Miranda, L. Hou, B. Barbiellini, R. S. Markiewicz, J.-X. Zhu, G. Kresse, A. Bansil, J. Sun, and R. Zhang, "Accurate electron-phonon interactions from advanced density functional theory," PRX Energy **5**, 013002 (2026).
[24]S. Jana, A. Bhattacharjee, S. Mahakal, S. Śmiga, and P. Samal, "Meta-GGA dielectric-dependent and range-separated screened hybrid functional for reliable prediction of material properties," Phys. Rev. B **111**, 115125 (2025).
[25]S. Kümmel and L. Kronik, "Orbital-dependent density functionals: Theory and applications," Rev. Mod. Phys. **80**, 3–60 (2008).
[26]L. Hedin, "New method for calculating the one-particle Green's function with application to the electron-gas problem," Phys. Rev. **139**, A796–A823 (1965).
[27]M. S. Hybertsen and S. G. Louie, "Electron correlation in semiconductors and insulators: Band gaps and quasiparticle energies," Phys. Rev. B **34**, 5390–5413 (1986).
[28]F. Aryasetiawan and O. Gunnarsson, "The *GW* method," Rep. Prog. Phys. **61**, 237–312 (1998).
[29]J. P. Perdew, R. G. Parr, M. Levy, and J. L. Balduz, "Density-functional theory for fractional particle number: Derivative discontinuities of the energy," Phys. Rev. Lett. **49**, 1691–1694 (1982).
[30]J. P. Perdew and M. Levy, "Physical content of the exact Kohn-Sham orbital energies: Band gaps and derivative discontinuities," Phys. Rev. Lett. **51**, 1884–1887 (1983).
[31]E. J. Baerends, O. V. Gritsenko, and R. van Meer, "The Kohn–Sham gap, the fundamental gap and the optical gap: the physical meaning of occupied and virtual Kohn–Sham orbital energies," Phys. Chem. Chem. Phys. **15**, 16408–16425 (2013).
[32]A. Aouina, M. Gatti, S. Chen, S. Zhang, and L. Reining, "Accurate Kohn-Sham auxiliary system from the ground-state density of solids," Phys. Rev. B **107**, 195123 (2023).
[33]S. Riemelmoser, M. Kaltak, and G. Kresse, "Optimized effective potentials from the random-phase approximation: Accuracy of the quasiparticle approximation," J. Chem. Phys. **154**, 154103 (2021).
[34]W. Kohn and L. J. Sham, "Self-consistent equations including exchange and correlation effects," Phys. Rev. **140**, A1133–A1138 (1965).
[35]A. Seidl, A. Görling, P. Vogl, J. A. Majewski, and M. Levy, "Generalized Kohn-Sham schemes and the band-gap problem," Phys. Rev. B **53**, 3764–3774 (1996).
[36]J. P. Perdew, W. Yang, K. Burke, Z. Yang, E. K. U. Gross, M. Scheffler, G. E. Scuseria, T. M. Henderson, I. Y. Zhang, A. Ruzsinszky, H. Peng, J. Sun, E. Trushin, and A. Görling, "Understanding band gaps of solids in generalized Kohn–Sham theory," Proc. Natl. Acad. Sci. U.S.A **114**, 2801–2806 (2017).
[37]P. Borlido, T. Aull, A. W. Huran, F. Tran, M. A. L. Marques, and S. Botti, "Large-scale benchmark of exchange–correlation functionals for the determination of electronic band gaps of solids," J. Chem. Theory Comput. **15**, 5069–5079 (2019).
[38]P. Borlido, J. Schmidt, A. W. Huran, F. Tran, M. A. L. Marques, and S. Botti, "Exchange-correlation functionals for band gaps of solids: benchmark, reparametrization and machine learning," npj Comput. Mater. **6**, 96 (2020).
[39]M. Kothakonda, A. D. Kaplan, E. B. Isaacs, C. J. Bartel, J. W. Furness, J. Ning, C. Wolverton, J. P. Perdew, and J. Sun, "Testing the r^{2}SCAN density functional for the thermodynamic stability of solids with and without a van der Waals correction," ACS Materials Au **3**, 102–111 (2022).
[40]L. Kronik and S. Kümmel, "Piecewise linearity, freedom from self-interaction, and a Coulomb asymptotic potential: three related yet inequivalent properties of the exact density functional," Phys. Chem. Chem. Phys. **22**, 16467–16481 (2020).
[41]A. D. Becke, "A new mixing of Hartree–Fock and local density-functional theories," J. Chem. Phys. **98**, 1372–1377 (1993).
[42]A. D. Becke, "Density-functional thermochemistry. III. The role of exact exchange," J. Chem. Phys. **98**, 5648–5652 (1993).
[43]A. D. Becke, "Density-functional thermochemistry. IV. A new dynamical correlation functional and implications for exact-exchange mixing," J. Chem. Phys. **104**, 1040–1046 (1996).
[44]T. Shimazaki and Y. Asai, "Band structure calculations based on screened Fock exchange method," Chem. Phys. Lett. **466**, 91–94

(2008).
[45]D. Wing, G. Ohad, J. B. Haber, M. R. Filip, S. E. Gant, J. B. Neaton, and L. Kronik, "Band gaps of crystalline solids from Wannier-localization–based optimal tuning of a screened range-separated hybrid functional," Proc. Natl. Acad. Sci. **118**, e2104556118 (2021).
[46]S. E. Gant, J. B. Haber, M. R. Filip, F. Sagredo, D. Wing, G. Ohad, L. Kronik, and J. B. Neaton, "Optimally tuned starting point for single-shot *GW* calculations of solids," Phys. Rev. Materials **6**, 053802 (2022).
[47]J. Yang, S. Falletta, and A. Pasquarello, "Range-separated hybrid functionals for accurate prediction of band gaps of extended systems," npj Comput. Mater. **9**, 108 (2023).

Detailed responses to the Reviewers' reports.

In the following point-to-point response, we address comments by the three anonymous Reviewers. Before addressing the comments in detail, we want to thank all Reviewers for the valued time and effort dedicated to our manuscript. The Reviewers' comments are given in *blue italic text,* while our responses are given in black plain text.

Reviewer #1

Even with the revisions that the authors have made to their manuscript, I still maintain my previous assessment that the overall novelty of the work does not warrant its publication in Nature Communication.

We acknowledge the opinion of the Reviewer, and thank them for previous feedback which had allowed us to improve our manuscript regarding the connection of COHSEX and GKS theory, the self-consistent DDH for the interesting case of InSb, and the application of DD-r^{2}SCANH to transition metal oxides.

Reviewer #2

The authors have satisfactorily addressed most of my concerns, and I recommend the manuscript for publication in its current form.

We appreciate the support of the Reviewer for publication in *Nature Communications* and thank them for many helpful suggestions in the previous round of review.

Reviewer #3

This is reviewer #3, and I have went through the responses, including those to the other reviewers. I am satisfied with their responses except for the point about the Coulomb hole, which in my opinion is a flaw of this manuscript in particular for Nature Communications. I went through the new section "Theoretical background", which is very helpful for understanding the picture the authors have. I can follow all of them now. But this raises serious doubts on the theoretical foundation for using the Coulomb hole in the title.

Basically there are two different branches that the authors would like to connect: 1. The generalized Kohn-Sham DFT which has a firm foundation for the ground state energy and electron density, but their orbitals in general don't have physical meanings except for the frontier orbitals; 2. The quasiparticle energies and associated self-energy in GW, which has clear physical meanings. Equations 2-8 are all fine on their own domains, but jumping from Eq Sigma_COH of Eq(6) to Eq (9) is not very clear to me and involves too many approximations there. The most difficult and confusing thing to me is how to connect total energy expression of GKS of Eq (9) to the self-energy expression of Eq (6). Is it just a handwaving argument. If not, the authors have to break it down into steps with supporting evidences.

To be clear, I am not saying that the authors were wrong. The connection could be right and could be kept in the "Theoretical background" (although I suggest the authors to tune down the tone), but there were no clear derivation or strong arguments, and therefore I suggest the authors to remove "Climbing Jacob's Ladder for the Coulomb hole". I think "Dielectric-dependent hybrid functional based on meta-GGA" is a good title by itself for the novelty and performance.

We thank the Reviewer for the interest and their careful consideration of this point. We are glad that the Reviewer now finds our theoretical discussion helpful, and acknowledge that some points were still not immediately obvious and deserve further attention. Indeed, there a few intermediate steps connecting the quasiparticle equation *GW* theory to GKS, and not all of them had been clearly lined out. Briefly, (i) one makes the COHSEX approximation, (ii) one replaces the dielectric function by the constant ε_∞^{-1}, and (iii) the COH potential is approximated by a semilocal DFT potential, i.e. $\Sigma_{\mathrm{COH}} \approx (1 - 1/\varepsilon_\infty v_{\mathrm{x}}^{\mathrm{DFT}}) + v_{\mathrm{c}}^{\mathrm{DFT}}$ [Eq. (12) in the revised manuscript]. This means the quasiparticle equation is reduced to a GKS equation for a global hybrid functional with $\alpha = 1/\varepsilon_\infty$. The corresponding energy functional is the usual expression for the DDH.

We have reworked the Theory section to better outline these intermediate steps. Furthermore, we have carefully reconsidered the title of our manuscript and adapted the more concise version: "Dielectric-dependent hybrid functional based on meta-GGA" as suggested by the Reviewer. We have also made changes to the abstract and to the discussion to better reflect the current title.

Other changes

Fig. 1(b): We have changed the ordinate label from "band gap accuracy" to "band gap MARE". Now, it is immediately clear that smaller bars signify better accuracy, thus avoiding ambiguity for the reader.

Detailed responses to the Reviewers' reports.

In the following point-to-point response, we address remaining comments by the anonymous Reviewer #3. The Reviewers' comments are given in *blue italic text,* while our responses are given in black plain text.

Reviewer #3

This is reviewer #3. I am satisfied with the authors's response, and recommend for publication of this manuscript. One more thing to add for the authors to consider. SCF DDH-r2SCAN opens a band gap for InSb which is a metal from r2SCAN. Will it be possible DDH-r2SCAN spuriously opens a band gap for real metals in some rare cases? Maybe the authors can add some discussions on it.

We are glad that the Reviewer is satisfied with our response to their previous comments, and appreciate their recommendation for publication in *Nature Communications*.
For some rare cases it has been reported that hybrid functionals with fixed mixing parameters falsely open the band gap for a real metal.[1] Indeed, the Reviewer is correct that this implies that there is a theoretical possibility that self-consistent DDH would also yield a false semiconducting phase here. To the best of our knowledge, this has not been demonstrated thus far, however. The problem is less severe than the opposite case of a false metallic ground state for a real semiconductor, since the existence of the fixed point $\alpha = 0$ guarantees that there is always some basin of attraction for a real metallic phase (assuming the DFT base functional correctly yields a metallic phase).

We added the following comment (lines 344-348 in the revised Supplementary Note 8):

"We also note that in rare cases the opposite problem can occur, namely standard hybrid functionals yielding a false semiconducting ground state for a real metal.[1] This might also imply that self-consistent DDH could have some basin of attraction for the false semiconducting solution. However, in this opposite case the fixed point $\alpha = 0$ guarantees a correct metallic solution for self-consistent DDH provided that the DFT base functional also yields a metallic ground state."

We thank the Reviewer for their valued contributions throughout the entire review process.

References

[1] J. W. Furness, Y. Zhang, C. Lane, I. G. Buda, B. Barbiellini, R. S. Markiewicz, A. Bansil, and J. Sun, "An accurate first-principles treatment of doping-dependent electronic structure of high-temperature cuprate superconductors," Commun. Phys. **1**, 11 (2018).

REFEREE COMMENTS ON THE MANUSCRIPT: CLIMBING JACOB'S LADDER FOR THE COULOMB HOLE: DIELECTRIC-DEPENDENT HYBRID FUNCTIONAL BASED ON META-GGA

The authors propose a dielectric-dependent hybrid (DDH) functional, termed DD-r^2SCANH, in which the PBE exchange–correlation base of conventional DDH schemes is replaced by the r^2SCAN meta-GGA. This modification substantially improves the description of electronic structures, particularly for narrow-gap semiconductors where PBE fails qualitatively. The resulting accuracy is comparable to that of many-body GW methods but at a significantly reduced computational cost.

Notably, the proposed framework can be extended to other GGA and meta-GGA functionals, such as TPSS- or MVS-type functionals, and the authors are encouraged to comment explicitly on this generalizability.

Apart from this, I suggest the following points be addressed before the manuscript can be considered for publication in *Nature Communications*:

1. **Placement of the "Climbing Jacob's Ladder for the Coulomb Hole" section:** This discussion could be more appropriately placed before the *Results* section, for instance, under a new subsection titled *Theoretical Background* or similar.

2. **Transition-metal oxides and Cu-chalcopyrites:** While the manuscript comments on the performance of r^2SCAN for transition-metal oxides and Cu-chalcopyrites, no corresponding results are presented. It would strengthen the paper to include at least one representative case, such as FeO in the AFM-II phase or $Cu_3(Sb/As)Se_4$, to illustrate the functional's performance for correlated systems.

3. **Behavior of ϵ_∞^{-1} in Fig. 3(b):** In Fig. 3(b), one value of ϵ_∞^{-1} appears to approach zero for r^2SCAN. The authors should discuss the physical reason behind this trend and its implications.

4. **Use of experimental dielectric constants:** All band-gap calculations are performed using experimental ϵ_∞^{-1}. The authors should comment on what would happen if ϵ_∞^{-1} were computed self-consistently within DD-r^2SCANH. Even if the f_{xc} kernel contribution is neglected, this point deserves discussion.

5. **Ionization potentials and self-consistency:** Have the authors attempted to compute ionization potentials fully self-consistently, including the evaluation of ϵ_∞^{-1} within DD-r^2SCANH? Clarifying this would improve the methodological transparency.

6. **Systems that remain metallic under r^2SCAN:** In Table IV, results based on ϵ_∞^{-1} obtained from r^2SCAN are shown. How are systems treated where r^2SCAN incorrectly predicts metallicity? The authors should discuss the stability and applicability of the DDH correction in such cases.

7. **Relevance of recently proposed meta-GGAs:** The authors may wish to comment on the potential use of recently developed meta-GGA functionals, such as LAK or r^2SCAN-L, within the DDH framework.

8. **Spin–orbit coupling (SOC) and zero-point renormalization (ZPR):** In Table S2, it is unclear whether the error statistics include both SOC and ZPR corrections. If SOC effects are not included, the authors are encouraged to report SOC-corrected band gaps explicitly in each column.

9. **Overestimation of band gaps:** In some cases, DD-r^2SCANH appears to overestimate the band gaps relative to experiment. The authors should highlight and discuss these deviations separately, as they may indicate limitations of the dielectric screening model or the underlying functional.

Overall, the proposed DD-r^2SCANH functional represents a valuable step forward in the development of non-empirical, dielectric-dependent hybrid functionals. Addressing the points raised above will substantially improve the manuscript's completeness and impact.
